# Engineering covalent small molecule–RNA complexes in living cells

**DOI:** 10.1038/s41589-024-01801-3

**Published:** 2025-01-06

**Authors:** Raphael Bereiter, Laurin Flemmich, Kamila Nykiel, Sarah Heel, Stephan Geley, Malou Hanisch, Clemens Eichler, Kathrin Breuker, Alexandra Lusser, Ronald Micura

**Affiliations:** 1 University of Innsbruck, Institute of Organic Chemistry and Center for Molecular Biosciences (CMBI), Innsbruck, Austria; 2 Institute of Molecular Biology, Biocenter, Medical University of Innsbruck, Innsbruck, Austria; 3 Institute of Pathophysiology, Biocenter, Medical University of Innsbruck, Innsbruck, Austria

**Keywords:** RNA, Organic chemistry

## Abstract

Covalent labeling of RNA in living cells poses many challenges. Here we describe a structure-guided approach to engineer covalent RNA aptamer–ligand complexes. The key is to modify the cognate ligand with an electrophilic handle that allows it to react with a guanine at the RNA binding site. We illustrate this for the preQ_1_-I riboswitch, in vitro and in vivo. Further, we demonstrate the versatility of the approach with a covalent fluorescent light-up aptamer. The coPepper system maintains strong fluorescence in live-cell imaging even after washing, can be used for super-resolution microscopy and, most notably, is uniquely suited for fluorescence recovery after photobleaching to monitor intracellular RNA dynamics. In addition, we have generated a Pepper ligand with a second handle for bioorthogonal chemistry to allow easily traceable pull-down of the covalently linked target RNA. Finally, we provide evidence for the suitability of this tethering strategy for drug targeting.

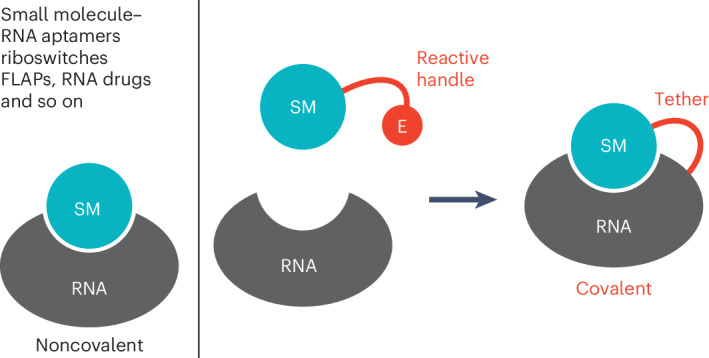

## Abstract (web summary)

Small-molecule ligands have been developed that covalently attach to their cognate RNA aptamers in vitro and in living cells. This strategy opens up new avenues for RNA imaging applications (for example, FRAP), for direct fluorescence detection of RNA pull-downs or for covalent RNA-targeted drug design.

## Main

Progress in RNA research hinges on our capacity to manipulate and thoroughly investigate RNA using precise chemical methodologies, both within controlled laboratory settings and within living cells^1–4^. Current tools for studying RNA function rely predominantly on noncovalent binding between an RNA and its corresponding ligand or receptor^5–7^. Unfortunately, the realm of covalent RNA labeling techniques^8–14^, which could potentially rival the breadth and adaptability seen with contemporary protein labeling strategies, remains insufficiently explored at present.

Several activity-based probes have been designed and used to identify naturally occurring unusually reactive RNAs in the transcriptome^15–22^. Moreover, in vitro selection approaches challenging combinatorial RNA libraries have been used to identify RNAs with reactivity toward specific electrophiles^8,9,20,23–25^. Despite these efforts, progress to selectively and covalently label RNA with small chemical entities and without the need for protein enzymes has been slow. The current examples are largely limited to RNA probes with reactive epoxide, halo-carbonyl, diaziridine and chlorambucil moieties^9,17^. However, these probes suffer from rather low efficacy and slow reaction rates or require light for activation. If in addition to such a crosslinking module, the compounds carry an affinity tag (such as alkyne or biotin), they have been used for Chem-CLIP (chemical crosslinking and isolation by pull-down) experiments to screen RNA pools (of both natural or synthetic origin) for potential targets^17,26^.

**Fig. 1:**
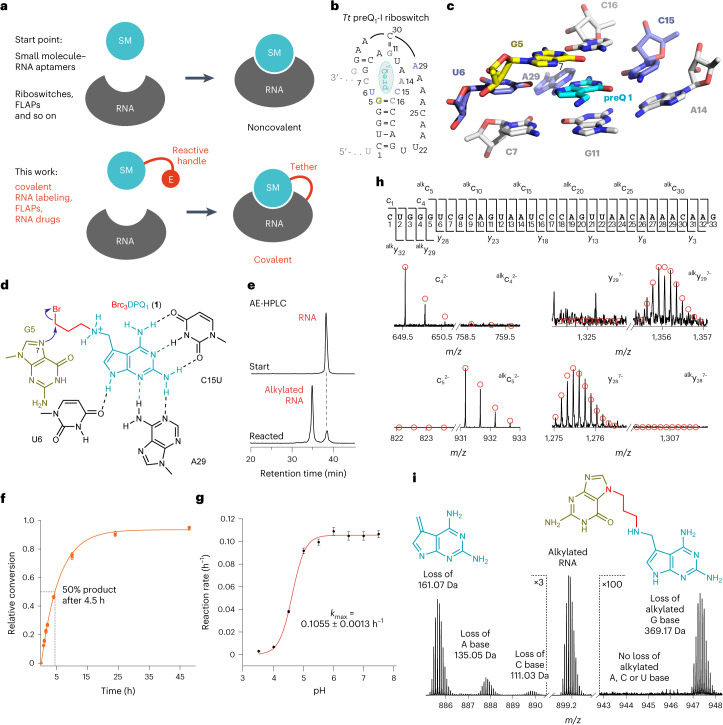
Covalent tethering of ligand–RNA complexes. **a**, Key is the ligand modification with a short handle and an electrophile (E) retaining initial ligand–RNA contacts. **b**, Secondary structure of *Tt* preQ_1_-I riboswitch (minimal aptamer motif, black; reactive guanosine, yellow; ligand, cyan). **c**, Stick representation of preQ_1_ binding pocket (Protein Data Bank (PDB) 3Q50). The ligand (cyan) is in close proximity to the N7 nucleophile of G5 (yellow). **d**, Structure-based design for ligand derivatization suggests 3-bromopropyl as reactive handle (Brc_3_DPQ_1_). **e**, Incubation of Brc_3_DPQ_1_ and 33 nt *Tt* C15U preQ_1_ RNA aptamer analyzed by AE-HPLC indicates a major alkylation product. **f**, Time course of the reaction (2.5 μM RNA, 125 μM Brc_3_DPQ_1_, 100 mM KCl, 2.0 mM MgCl_2_, 50 mM MES pH 6.0, 37 °C). Individual data points are shown as open circles. Mean values (filled circles) ± s.e.m. are shown. Measurements were performed in three independent experiments. **g**, pH dependence of the reaction rate (conditions as in **f**, except 60 μM Brc_3_DPQ_1_; pH values as indicated; for a relative conversion–time plot at different pH values see Extended Data Fig. 1a; for HPLC traces see Extended Data Fig. 1b. **h**, FT-ICR mass spectrometric characterization of the covalent c_3_DPQ_1_–RNA complex. CAD of (M–nH)^*n*–^ ions of RNA produces c and y fragment ions from RNA backbone cleavage. Fragment-ion map illustrating sequence coverage from CAD (top). MS signals of c_4_, c_5_ and complementary y_29_, y_28_ fragments from CAD of (M–9H)^9−^ and (M–8H)^8−^ ions reveal the site of alkylation (G5); calculated isotopic profiles (red open circles). **i**, Loss of c_3_DPQ_1_ alkylated guanine (right) in spectra from CAD of (M–12H)^12−^ ions of RNA is direct evidence for G nucleobase alkylation. Alkylated guanine is lost as a deprotonated species such that the product carries only 11 charges and appears at a higher *m/z* (~947.5). SM, small molecule. Source data

Here we explored new strategies to specifically and covalently link small molecule ligands to their cognate RNA targets (Fig. 1a). We chose well-characterized ligand–RNA aptamer systems that either occur naturally, such as in messenger RNA (mRNA) riboswitches^27^, or were selected in vitro, such as fluorescent light-up aptamers (FLAPs)^28–34^. We developed new chemistry to engineer these noncovalent into covalent ligand–RNA complexes by attaching simple reactive handles to the ligand that would not interfere with the original recognition pattern but enable covalent bonding with a guanosine nucleobase at the binding site. We demonstrate the functionality and versatility of this approach by engineering a covalent preQ_1_ riboswitch system and by introducing a covalent FLAP (coFLAP) system. Furthermore, we show the in vivo applicability of the modified ligands and provide evidence for their potential use in RNA-targeted drug design^15^.

## Results

### Covalent tethering of the preQ_1_ RNA aptamer

For engineering a noncovalent ligand–RNA aptamer system into its covalent counterpart, we first focused on the well-characterized preQ_1_ class I riboswitches (preQ_1_-I) (Fig. 1b)^35^. Notably, targeting this class of riboswitches with covalent ligands including a number of electrophiles, was successful only when carbene-generating photoactivatable diaziridine-modified ligands were applied^17^. Since the reaction was site-specific, it attracted our interest and encouraged us to revisit the preQ_1_-I aptamer with new ligands containing thus far disregarded electrophiles.

Our assessment of the preQ_1_ RNA binding pocket in the *Thermoanaerobacter tengcongensis* preQ_1_ riboswitch suggested that the aminomethyl group of preQ_1_ (7-aminomethyl-7-deazaguanine) is suitable for derivatization with a short (three carbons long) handle providing a mild electrophile for reaction with the N7 atom of guanine-5 (G5) (Fig. 1c). More generally, our three key guidelines for the intended ‘tethering approach’ were (1) bromide as electrophile, (2) guanine N7 as nucleophile and (3) the replacement of the ligand’s Watson–Crick face by an alternative recognition pattern. These features were considered advantageous for the following reasons: first, primary alkyl halides have been used to great success as covalent handles for protein labeling^36,37^, crosslinking^38,39^ and drugging^40^. With regard to nucleic acids, alkyl halides are, however, relatively underexplored; only halides of increased electrophilic potency, such as alpha-halo carbonyls^11,14,25,41^ and nitrogen (half-)mustards^17,26^ have been applied. Yet, we envisioned that positioning of a short 3-bromopropyl handle in the tight preQ_1_ binding pocket is ideal for an S_N_2 reaction with the N7-G5 nucleophile and should compensate for the inherently less reactive alkyl bromide, harnessing the merits of reduced side-reactivity^42^, increased stability and decreased cellular toxicity^43^. Second, the choice of the N7 atom of a guanine as the privileged nucleophile relies on theoretical and experimental studies that report N7 nucleophilicity enhancements caused by stacking in continuous purine runs (here G3-G4-G5)^44,45^. Third, aiming at in-cell applications, we sought to include an additional layer of orthogonality with respect to the natural riboswitch system. Following earlier work^46^, we decided to make use of a single mutation of a key cytidine residue within the preQ_1_-I aptamer that shifts the affinity away from guanine-faced preQ_1_ ligands in favor of artificial 2,6-diaminopurine-faced (DPQ_1_) ligands, creating an orthogonal riboswitch–ligand pair.

**Extended Data Fig. 1:**
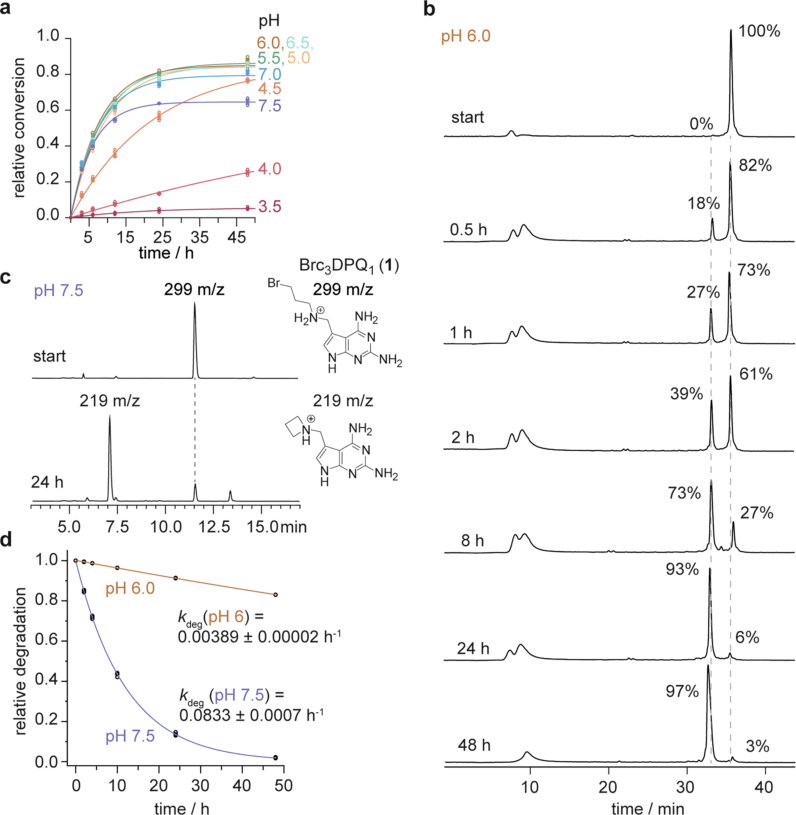
Further evaluation data for Brc_3_DPQ_1_ covalent tethering with the preQ_1_ riboswitch. **a**, Relative conversion–time plot at different pH for the reaction between Brc_3_DPQ_1_ and C15U preQ_1_ RNA (conditions: 2.5 μM RNA, 60 μM Brc_3_DPQ_1_, 100 mM KCl, 2.0 mM MgCl_2_, 50 mM buffer, 37 °C, pH values as indicated). Mean values (filled circles) ± s.e.m. are shown (n = 3). **b**, Time course of the reaction between Brc_3_DPQ_1_ and *Tt* preQ_1_ RNA (conditions as for **a**) followed by AE HPLC analysis at the time points indicated; conditions as in **a** but 125 μM Brc_3_DPQ_1_. **c**, Brc_3_DPQ_1_ stability at pH 7.5 (50 mM MOPS) at time points 0 and 24 h analyzed by LC-MS experiments. The major degradation product is consistent with intramolecular cyclization; Rp-HPLC (XBridge BEH C18 Column): 0-15% acetonitrile in aqueous TFA (0.1%), in 15 min. **d**, Brc_3_DPQ_1_ stability at pH 6.0 and pH 7.5, respectively. Mean values (filled circles) ± s.e.m. are shown (n = 3). Source data

Thus, we synthesized the ligand Brc_3_DPQ_1_ (Fig. 1d) based on previous experience with preQ_1_ synthesis and derivatization^47^. Notably, when Brc_3_DPQ_1_ was incubated with the *T. tengcongensis* preQ_1_ RNA aptamer containing the C15U replacement under near-physiological conditions, substantial amounts of an RNA adduct were obtained (Fig. 1e–g and Extended Data Fig. 1), and yields were further increased to >90% under optimized conditions by lowering the pH of the reaction buffer to 6.0 (Extended Data Fig. 1a). The pronounced shift of the product toward a shorter retention time on anion-exchange high-performance liquid chromatography (AE-HPLC) indicated the introduction of a positive charge. We isolated the product and analyzed it by high-resolution Fourier-transform ion cyclotron resonance (FT-ICR) mass spectrometry (MS), revealing the expected signal with a 218.12 Da mass increase consistent with c_3_DPQ_1_-alkylated RNA. Backbone cleavage through collisionally activated dissociation (CAD) generated a complete set of c and y fragments enabling sequence determination (Fig. 1h). The fragment mass values unequivocally revealed the site of alkylation at the G5 nucleoside (Fig. 1h). Furthermore, the loss of 2,6-diamino-7-methylene-7-deazapurine was obvious (Fig. 1i, left), and, importantly, the loss of c_3_DPQ_1_-alkylated guanine from RNA ions (Fig. 1i, right) provided direct evidence that the c_3_DPQ_1_ group was located at the nucleobase.

**Fig. 2:**
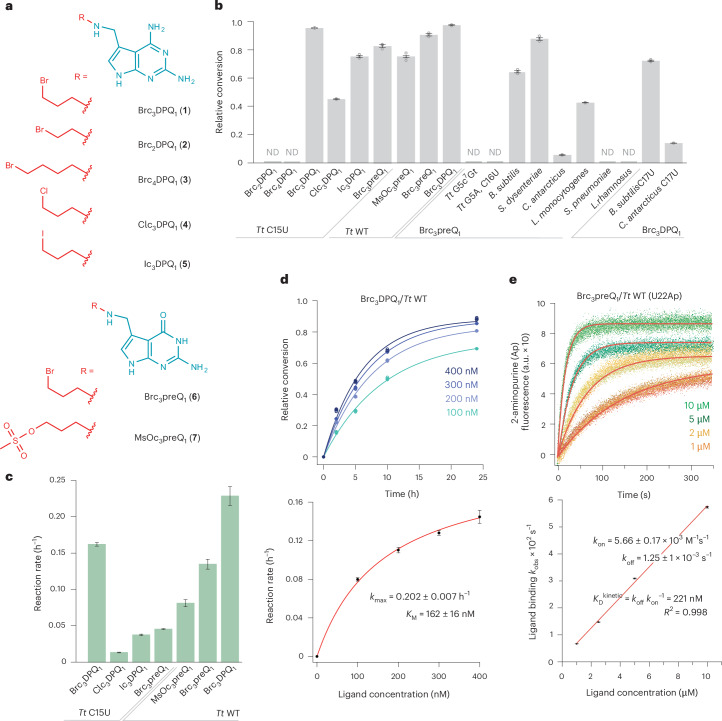
Characterization of *Tt* preQ_1_ aptamer tethering. **a**, Chemical structures of ligands with reactive handles tested in this study. **b**, preQ_1_ aptamer reactivity analysis using diverse ligands, nucleobase mutagenesis and atomic mutagenesis; conditions were: 2.5 μM RNA, 125 μM ligand, 100 mM KCl, 2.0 mM MgCl_2_, 50 mM MES pH 6.0, 37 °C, end point (48 h); bars (gray) show mean ± s.e.m. (open circles show individual data points, *n* = 3 independent experiments); ND, not detected. **c**, Reaction rates of the different ligands versus C15U and/or WT *Tt* RNAs; conditions as in **b**; reaction rate constants are reported as fit value ± fit error and were extracted from the individual conversion versus time plots shown in Extended Data Fig. 2a. **d**, The reaction rate is dependent on ligand concentration (depicted for Brc_3_DPQ_1_ versus WT *Tt* RNA). Relative conversion versus time (top plot) and reaction rate versus ligand concentration (bottom plot). The observed rate constants *k* were determined based on HPLC trace analysis at four concentrations of Brc_3_preQ_1_, ranging from 100 to 400 nM. For detailed conditions, see Methods. The red line represents a curve fit to *k* = *k*_max_[Brc_3_DPQ_1_]/(*K*_M,app_ + [Brc_3_DPQ_1_]) where *k*_max_ is the maximal rate. Individual data points (open circles) (*n* = 3 independent experiments), mean ± s.e.m. (black circles); see Extended Data Fig. 2b for Brc_3_preQ_1_ versus WT *Tt* riboswitch and Extended Data Fig. 2c for Brc_3_DPQ_1_ versus C15U *Tt* riboswitch. **e**, Ligand recognition (initial noncovalent binding) monitored by fluorescence emission of *Tt* RNA with U22Ap mutation (Ap, 2-aminopurine). Ap fluorescence versus time (top plot) and *k*_obs_ versus ligand concentration (bottom plot). Individual data points (open circles) (*n* = 3 independent experiments), mean ± s.e.m. (black circles). *K*_D_ is estimated from *k*_on_ and *k*_off_ (rate of dissociation) (see equation in **e**). *K*_D_^kinetic^ is the equilibrium dissociation constant, calculated as *k*_off_/*k*_on_. Source data

Next, we set out to more systematically study the effects of linker length, leaving group properties and potential alternative nucleophiles. Shortening or lengthening of the handle by only one CH_2_ group (Brc_2_DPQ_1_, Brc_4_DPQ_1_) abolished N7-G5 alkylation (Fig. 2a,b) while changing the leaving group from bromide to chloride or iodide (Clc_3_DPQ_1_, Ic_3_DPQ_1_) led to lower reaction yields. Furthermore, we synthesized and tested guanine-faced Brc_3_preQ_1_ on both wild-type (WT) and the C15U-mutated *Tt* RNA aptamer and found only slightly lower alkylation compared to Brc_3_DPQ_1_ (Fig. 2a,b). Similarly, for the mesylated congener (MsOc_3_preQ_1_), alkylation yields still amounted to about 80% of those obtained with Brc_3_DPQ_1_ (Fig. 2b).

Next, we tested whether the N7 atom of G5 was indeed the site of alkylation by using atomic mutagenesis. As expected, when G5c^7^G preQ_1_ RNA was incubated with Brc_3_preQ_1_, no reaction was observed (Fig. 2b). A G5A C16U mutated RNA also gave no reaction, demonstrating that the N7 of adenine is of insufficient nucleophilicity to react with the 3-bromopropyl handle of the ligand (Fig. 2b).

**Extended Data Fig. 2:**
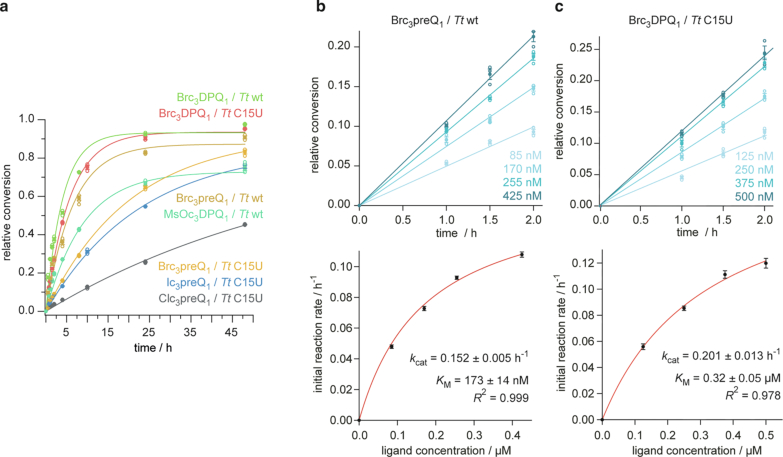
Reactivity scope of *Tt* preQ_1_ aptamer tethering. **a**, Relative conversion–time plot for the different ligands *vs* wt and C15U *Tt* RNAs. Individual data points (open circles) (n = 3 independent experiments), mean ± s.e.m. (black circles); conditions: 2.5 μM RNA, 125 μM ligand, 100 mM KCl, 2.0 mM MgCl_2_, 50 mM MES buffer pH 6.0, 37 °C). **b**, The reaction rate is dependent on ligand concentration (depicted for Brc_3_preQ_1_ vs wt *Tt* RNA). Relative conversion vs time (*top plot*) and initial reaction rate vs ligand concentration (*bottom plot*). The observed rate constants *k*_obs_ were determined based on HPLC trace analysis at four concentrations of Brc_3_preQ_1_, ranging from 85 to 435 μM (conditions: 17 nM RNA, 100 mM KCl, 2.0 mM MgCl_2_, 50 mM MES buffer pH 6.0, 37 °C. The red line represents a curve fit to *k*_obs_ = *k*_max_[Brc_3_preQ_1_]/(*K*_M,app_ + [Brc_3_preQ_1_]). Individual data points (open circles) (n = 3 independent experiments), mean ± s.e.m. (black circles). **c**, Same as **b** but for Brc_3_DPQ_1_ *vs* C15U *Tt* RNA; Brc_3_DPQ_1_ ranging from 125 to 625 μM (conditions: 25 nM RNA, 100 mM KCl, 2.0 mM MgCl_2_, 50 mM MES buffer pH 6.0, 37 °C. Source data

The combination of Brc_3_DPQ_1_ with WT *Tt* preQ_1_ RNA (‘mismatched’ to C15) gave the same yields as for its ‘cognate’ C15U mutant (Fig. 2b). Therefore, we determined reaction kinetics and found the fastest rate of all systems tested for Brc_3_DPQ_1_ alkylating WT *Tt* preQ_1_ RNA (Fig. 2c and Extended Data Fig. 2a). The reaction rate was dependent on Brc_3_DPQ_1_ concentration, with an apparent Michaelis constant *K*_M_ of about 162 nM (Fig. 2d and Extended Data Fig. 2b,c).

To estimate the rate of ligand binding, we applied a 2-aminopurine fluorescence spectroscopic approach termed 2ApFold (ref. ^48^) that allows for real-time monitoring of ligand-induced structural rearrangements of specific nucleobases. The U22Ap mutant^49^ of the preQ_1_ aptamer exhibited an estimated rate *k*_on_ of 5.66 ± 0.17 × 10^3^ M^−1^ s^−1^ for Brc_3_preQ_1_ binding (Fig. 2e), which is about half the binding rate of preQ_1_ (ref. ^49^). Note that because of the slight autofluorescence of Brc_3_DPQ_1_ that interfered with 2Ap fluorescence, it was not used in this assay.

To evaluate how stringent the sequence requirements of the binding pocket are for the alkylation reaction, we investigated several other preQ_1_ riboswitch scaffolds. Three highly related types of preQ_1_ class I aptamer are known, all of which containing the characteristic stem P1 terminal base pair G5-C as part of the base quartet that forms the floor of the binding pocket^50^. The preQ_1_-I type 3 aptamer from *Shigella dysenteriae* (class I_III_) was almost as reactive as the parent preQ_1_-I type 2 RNA from *T. tengcongensis* (class I_II_), followed by the preQ_1_-I type 1 RNA from *Listeria monocytogenes* (class I_I_) and the preQ_1_-I type 2 RNA from *Bacillus subtilis* (class I_II_) (Fig. 2b). Only the preQ_1_-I type 1 RNA from *Carnobacterium antarcticus* was less reactive; this specific RNA differs from the other class I riboswitches by the fact that it binds two ligands, one stacked on top of the other, in a single binding pocket^51^. Together, these observations suggest that the distinct nucleosides in the nonconserved sequence regions of the preQ_1_ loop L1 as well as in the 3′ tail give rise to subtle structural differences that may account for the variation in the G5 alkylation yields^52^.

Not unexpectedly, the preQ_1_ class II aptamers of *Streptococcus pneumoniae* and *Lactobacillus rhamnosus* were not reactive, consistent with the distinct architecture of their binding pockets and the different recognition mode of the ligand by a *trans*-Watson–Crick–Watson–Crick pair with cytosine^53^.

### In vivo evaluation of covalent preQ_1_ ligands

**Extended Data Fig. 3:**
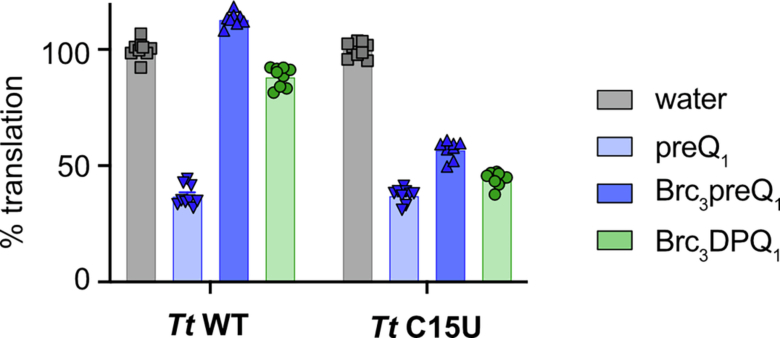
Translation regulation activity of covalent preQ_1_ ligands. *E. coli* bacteria transformed with reporter constructs expressing GFP under the control of either the *Tt* WT preQ_1_ riboswitch or the C15U mutant riboswitch were tested for GFP fluorescence after 6 h of growth in the presence of the indicated ligands. Translational inhibition is expressed relative to the mock (water) control after correction for cell number and background fluorescence. Mean ± SD of three independent experiments with three technical replicates each is shown. Source data

To investigate how a reactive preQ_1_ ligand might affect the in vivo regulatory properties of a preQ_1_ riboswitch (*T. tengcongensis)*, we generated two reporter constructs fusing either the wild-type (C15) *Tt* preQ_1_-I aptamer or the C15U-mutated aptamer sequence to the green fluorescence protein (GFP) coding sequence and monitored protein production in response to preQ_1_, Brc_3_preQ_1_ or Brc_3_DPQ_1_ in *Escherichia coli*. To avoid potential interference of endogenous preQ_1_ with the assay, we used the *E. coli* strain JW0434, which is incapable of queuosine synthesis^54^. The *Tt* preQ_1_ riboswitch acts as a negative regulator of translation by sequestering the Shine–Dalgarno sequence via ligand-triggered alternative RNA folding^47^. Comparison of fluorescence at 6 h after ligand addition revealed that the cognate preQ_1_ ligand repressed the WT construct by about 60% compared to vehicle control (H_2_O), while Brc_3_DPQ_1_ and Brc_3_preQ_1_ showed hardly any repression (Extended Data Fig. 3). Although the lack of inhibition by Brc_3_DPQ_1_ is consistent with the switch of WT Watson–Crick ligand recognition (C-preQ_1_) to mismatch recognition (C-Brc_3_DPQ_1_), the results with Brc_3_preQ_1_ indicate that the bromopropyl group interferes with riboswitch regulation in vivo potentially by reducing the *k*_on_ rate (Fig. 2e). In stark contrast, we observed that both modified ligands were active in combination with the C15U riboswitch, with Brc_3_DPQ_1_ being slightly more inhibitory than Brc_3_preQ_1_ (Extended Data Fig. 3). Taken together, the C15U mutation generated a riboswitch with relaxed ligand specificity that, unlike the WT riboswitch, can be regulated by preQ_1_ as well as the bromopropyl-modified 7-deazapurine ligands with efficiencies that are comparable to the natural preQ_1_ system. Detailed investigation about the exact role of the reactive handle in regulating riboswitch function will be a subject for future studies.

### A coFLAP

Over the past decade, the realm of fluorogen-activating aptamers (FLAPs) has emerged as a prominent class of synthetic functional nucleic acids predominantly for the purpose of tracking and visualizing RNA in cellular and molecular biology^32^. Researchers have identified numerous 30 to 100-nucleotide (nt)-long RNAs that can activate the fluorescence of various conditional fluorophores^29–33^.

Common to all known FLAPs is that their fluorophores are noncovalently bound to the aptamer. This can give rise to sensitivity issues in fixed and live-cell imaging simply because of washout of the ligand and loss of fluorescence signal. Encouraged by our successful tether development for preQ_1_ aptamers, we created a new class of FLAP systems that relies on covalently attached fluorophores.

**Fig. 3:**
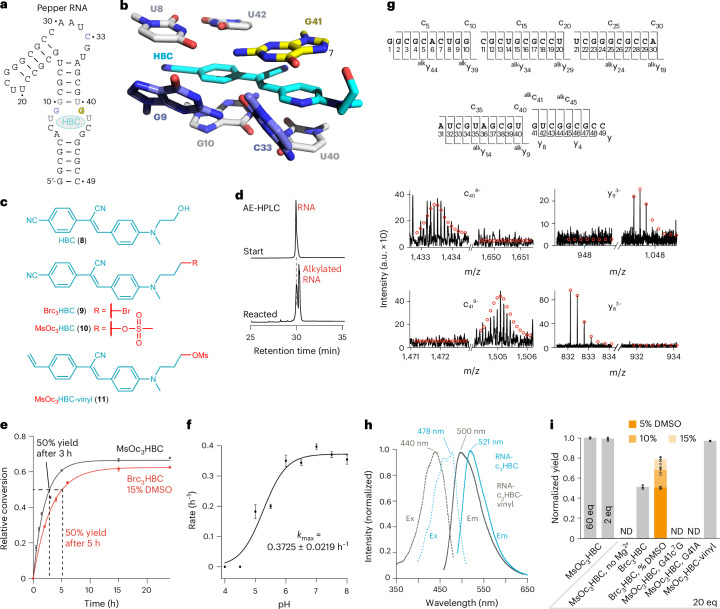
A coFLAP of Pepper. **a**, Sequence and secondary structure of the Pepper aptamer (nucleosides in gray and blue form the binding pocket; ligand (HBC) in cyan; reactive guanosine in yellow; same color code is used in **b**). **b**, Stick representation of the fluorophore binding pocket (PDB 7EOM). The HBC ligand (cyan) is in close proximity to the N7 nucleophile of G41 (yellow). **c**, Tested HBC ligands, with and without reactive handles (Brc_3_HBC, MsOc_3_HBC and MsOc_3_HBC-vinyl). **d**, Incubation of Brc_3_HBC and 49-nt Pepper RNA results in clean reaction to a major alkylation product as analyzed by AE-HPLC. **e**, Time course of the reaction (2.5 μM RNA, 50 μM MsOc_3_HBC or Brc_3_HBC, 150 mM KCl, 2.0 mM MgCl_2_, 50 mM MES buffer pH 6.0 (15% DMSO in case of Brc_3_HBC), 37 °C). Mean values (filled circles) ± s.e.m. are shown (*n* = 3). **f**, pH dependence of the MsOc_3_HBC reaction rate (conditions as in **e**, pH values as indicated; for relative conversion–time plot at different pH values see Extended Data Fig. 3a). **g**, FT-ICR mass spectrometric characterization of the covalent c_3_HBC–RNA complex. CAD of (M–nH)^n–^ ions of RNA produces *c* and *y* fragment ions from RNA backbone cleavage. Fragment-ion map illustrating sequence coverage from CAD of the alkylated Pepper RNA (top). MS signals of unmodified and alkylated c_40_, c_41_ and complementary y_9_, y_8_ fragments from CAD (M–11H)^11−^ and (M–12H)^12−^ ions reveal site of alkylation (G41); calculated isotopic profiles for unmodified and singly alkylated RNA are indicated by red open circles. **h**, Fluorescence absorption and emission (em) spectra of coPepper with c_3_HBC (cyan) and c_3_HBC-vinyl (dark gray). Ex, excitation. **i**, Pepper aptamer reactivity analysis using diverse conditions; bars show mean ± s.e.m. (*n* = 3). Source data

**Extended Data Fig. 4:**
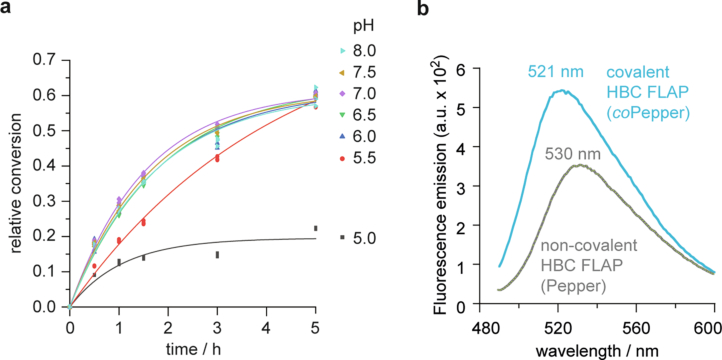
A covalent fluorescent light-up aptamer (*co*FLAP) examplified for Pepper. **a**, Relative conversion–time plot at different pH for the reaction between MsOc_3_HBC and Pepper RNA (conditions: 2.5 μM RNA, 50 μM with MsOc_3_DPQ_1_ or Brc_3_DPQ_1_, 150 mM KCl, 2.0 mM MgCl_2_, 50 mM buffer, 37 °C, pH values as indicated). Individual data points are shown as open circles. Mean values (filled circles) are reported ± s.e.m. Measurements were performed in triplicate. **b**, Comparison of fluorescence emission spectra of Pepper with non-covalently bound HBC (conditions: 0.5 µM RNA, 0.5 µM HBC, 100 mM KCl, 2.0 mM MgCl_2_, 50 mM MES buffer, pH 6.0) and c_3_HBC modified Pepper (conditions: 0.5 µM RNA-c_3_HBC, buffer conditions as above). Source data

To demonstrate this concept we chose the Pepper aptamer that comprises 49 nt (Fig. 3a) and recognizes a GFP fluorophore mimic, called HBC (Fig. 3b,c)^55,56^. We examined the three-dimensional structure of the Pepper binding pocket with respect to a possible chemical reaction between the N7 atom of a guanine and a reactive handle attached to HBC, and conceived the 3-bromopropyl-modified derivative Brc_3_HBC (Fig. 3c). Indeed, Brc_3_HBC promoted efficient covalent bond formation (Fig. 3d). Further optimization included the exchange of bromide to mesylate, which increased solubility of the modified HBC (MsOc_3_HBC) in aqueous solvents and further enhanced rates and yields (Fig. 3e). The reaction was robust over a wide pH range (Fig. 3f and Extended Data Fig. 4a). FT-ICR-MS analysis of the isolated product confirmed the anticipated 299.14-Da mass increase consistent with c_3_HBC-alkylated RNA. Further, CAD experiments producing a complete set of *c* and *y* fragments revealed the site of alkylation at the G41 nucleoside (Fig. 3g). Moreover, because atomic mutagenesis of Pepper G41 to c^7^G41 gave no detectable product, the N7 atom of 41 must be the site of RNA tethering.

The HBC-RNA complex displays a well-defined fluorescent profile with excitation and emission maxima at 485 and 530 nm, respectively. It binds noncovalently to Pepper RNA with a dissociation constant *K*_d_ of about 3.5 nM, a fast on-rate of 6.6 × 10^5^ M^−1^ s^−1^ and a slow dissociation rate of 0.0023 s^−1^ (ref. ^55^). With the covalent tether, the excitation and emission maxima of MsOc_3_HBC shifted to 478 and 521 nm, respectively (Fig. 3h), and fluorescence emission intensity increased modestly by about 1.5-fold, consistent with the additional rigidification of the fluorophore obtained by the tether (Extended Data Fig. 4b).

Notably, covalent bond formation of the MsOc_3_HBC ligand worked efficiently even with only onefold excess of ligand over RNA, providing almost the same yields as for a 60-fold excess of ligand (Fig. 3i). Crosslinking, however, was not observed in the absence of Mg^2+^, which can be rationalized by the requirement of low Mg^2+^ concentrations for folding of Pepper into an HBC530 binding-competent structure (Fig. 3i). Notably, with the bromo-modified HBC530 ligand, high reaction yields were only achieved when the dimethylsulfoxide (DMSO) concentration in the buffer was raised to 15% (Fig. 3i). This is not required for the mesylated HBC, which makes it the preferred fluorophore derivative for cellular applications.

Finally, we generated a bifunctional HBC ligand containing an additional vinyl group (MsOc_3_HBC-vinyl) that is available for fast bioorthogonal reactions with tetrazines (Fig. 3c). We demonstrated that also this ligand can attach covalently to the aptamer (Fig. 3h,i). The fluorescence absorption and emission spectra were slightly blue-shifted, in accordance with the replacement of the cyano by a vinyl group (Fig. 3h). The MsOc_3_HBC-vinyl ligand can be used for affinity purification of crosslinked RNA targets with simultaneous monitoring of pull-down success by the inherent fluorescence signal as described below.

### Comparison of different ribozyme alkylation chemistries

**Extended Data Fig. 5:**
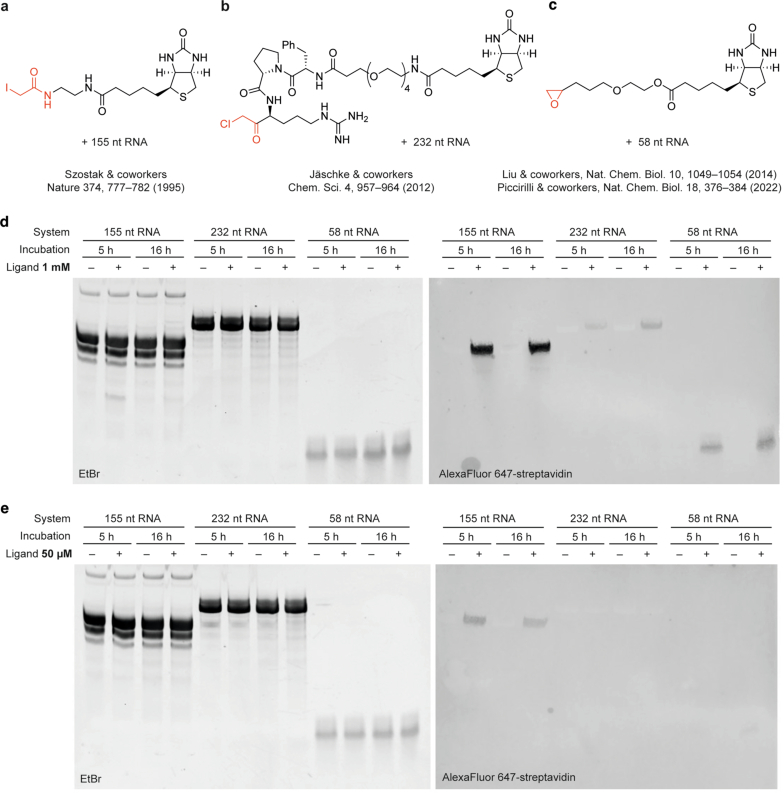
Comparison of self-alkylating ribozymes. **a-c**, Chemical structures of ligands for *in vitro* selected self-biotinylating ribozymes (with key references). **d**,**e** Reacted RNA was subjected to PAGE and stained with EtBr (*left panels*) followed by blotting to nylon membrane and visualization of biotin by AlexaFluor™647-streptavidin (*right panels)*. Reaction conditions: 2.5 µM RNA, 1 mM (**d**) or 50 µM (**e**) ligand in DMSO and water to 10 µl; 50 mM HEPES, pH 7.4, 100 mM KCl, 5 mM MgCl_2_, 5 h or 16 h at 37 °C. Representative images of two independent experiments per concentration are shown. Source data

**Extended Data Fig. 6:**
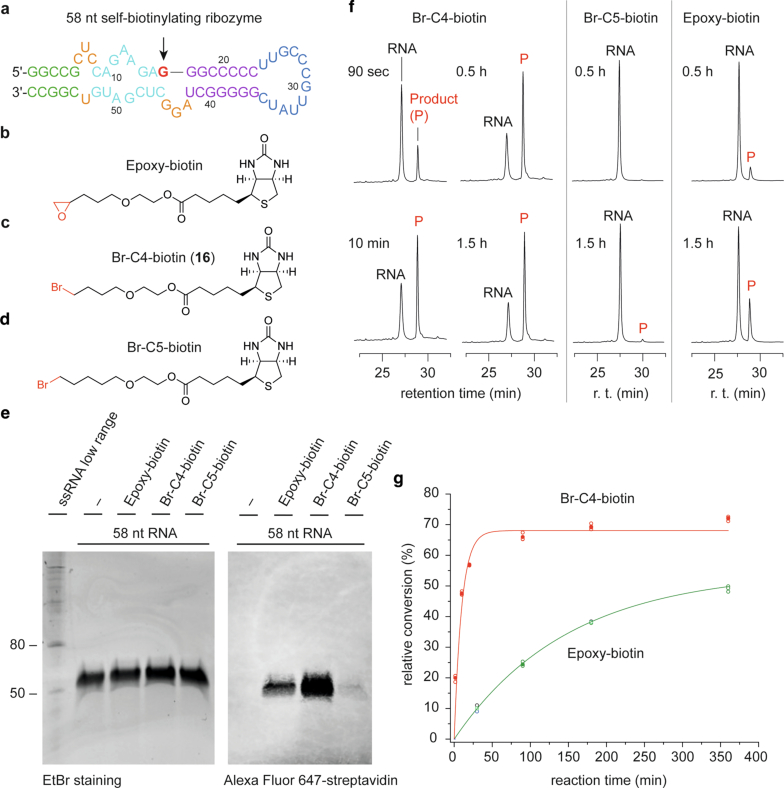
Comparison of the original epoxide-biotin ligand and bromoalkyl-biotin counterparts reacting with a self-biotinylating ribozyme. **a**, Secondary structure of the RNA sequence used. Site of alkylation is indicated by red color. Other colors indicate secondary structure elements. **b**-**d**, Chemical structures of ligands used for the comparison of their alkylation efficiencies. **e**, The RNA alkylation reactions were analyzed by PAGE, blotting to a nylon membrane, and visualization with EtBr staining (before blotting, *left*) and AlexaFluor™ 647-streptavidin (after blotting, *right*). Br-C4-biotin resulted in higher yields compared to epoxy-biotin; hardly any RNA alkylation was observed for Br-C5-biotin. Results from one experiment are shown. **f**, RNA alkylation reactions analyzed by RP-HPLC (Supelcosil LC-18-S, gradient: 0-20% B in 40 min; buffer A: 50 mM triethylammonium acetate (TEAA) pH 7.0, buffer B: acetonitrile (ACN). HPLC traces for individual time points of the reaction are depicted. **g**, Graph shows relative conversion vs reaction time to determine reaction kinetics (see Supplementary Table 1). Source data

One of the first alkylating RNAs described in the literature was a self-biotinylating ribozyme^8^. Then, RNA catalysts reacting with an inhibitor of serine proteases^25^ or with a fluorescein dye followed^41^. All of these rather large RNAs (155 to 232 nt) were in vitro selected for ligands providing either chloro- or iodoacetamide as the electrophile. More recently, a short self-biotinylating RNA (58 nt) was introduced using biotin with 2,3-disubstituted^9^ or monosubstituted^57^ epoxide handles. To compare the alkylation capabilities of the different systems under the same conditions, we synthesized three of these RNAs and reacted them with their respective ligands (that all contained a biotin moiety) (Extended Data Fig. 5). Detection of covalent products with AlexaFluor 647-labeled streptavidin after denaturing polyacrylamide gelelectrophoresis (PAGE) and northern blotting revealed that the 155-nt RNA with *N*-biotinoyl-*N*-iodoacetyl-ethylenediamine was the most reactive among the three alkylation systems (Extended Data Fig. 5). Next, we focused on the 58-nt long self-biotinylating ribozyme^57^ and replaced the original epoxide-biotin by the corresponding bromoalkyl-biotin substrate (Extended Data Fig. 6) enabling a direct comparison to our new alkylation chemistry. The rate of self-alkylation was more than 120-fold increased (Br-C4-EG-biotin), resulting in near quantitative yields after 30 min of reaction time (Extended Data Fig. 6). This example underlines the superiority and the broad applicability of the approach.

Finally, a comprehensive comparison of kinetics data found in the literature for other alkylating ribozymes to our reactive preQ_1_ and Pepper systems showed that the latter two exhibited by far the highest catalytic efficiencies (*k*_cat_/*K*_m_) (Supplementary Table 1). The superior catalytic properties, together with the small size of these aptamers and their activity at low Mg^2+^ concentrations, render the covalent ligand–aptamer systems generated in this study highly suitable for in vivo applications.

### In-cell ligand tethering of Pepper and preQ_1_ RNA aptamers

**Fig. 4:**
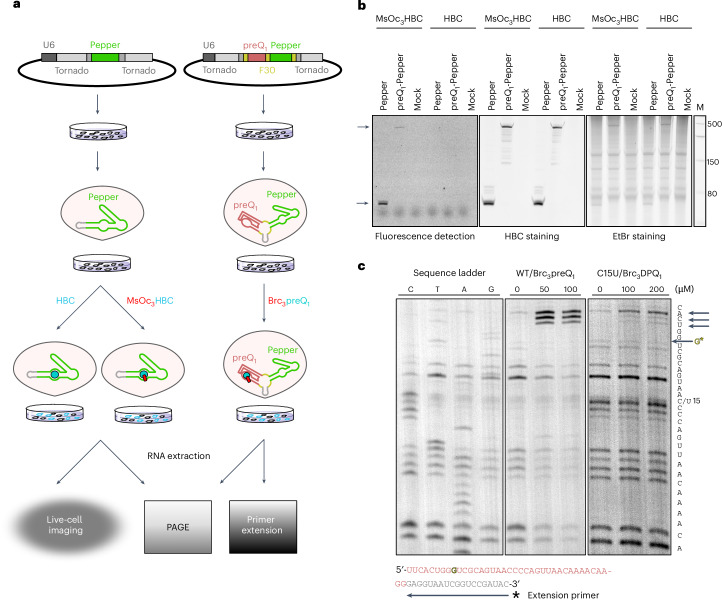
Intracellular tethering of Pepper and preQ_1_ RNA. **a**, Schematic depiction of the experimental setup. HEK293T cells were transfected with pTornado-Pepper (left) or pTornado-preQ_1_-Pepper (right), grown to enable expression of the circular pepper aptamer and then incubated with either HBC or MsOc_3_HBC (left) or Brc_3_preQ_1_ (right), followed by total RNA extraction, PAGE and fluorescence detection or primer extension. In other experiments, live-cell imaging with or without washing with PBS was performed. **b**, PAGE analysis shows that MsOc_3_HBC forms a covalent tether intracellularly and remains stably attached to the RNA during RNA extraction under denaturing conditions. Left panel, coPepper aptamers allow for direct fluorescence detection by a Typhoon imager: the fluorescent bands (first and second lane) correspond to the circular Pepper and preQ_1_-Pepper RNAs, respectively. Middle panel, gel was stained with HBC and imaged again: circular RNAs without covalently attached fluorophores become visible (fourth and fifth lane). Right panel, gel was stained with ethidium bromide (EtBr) to visualize total RNA. M, Low Range single-stranded RNA ladder (NEB). Representative image of three independent experiments is shown. HBC was tested once. **c**, Brc_3_preQ_1_ and Brc_3_DPQ_1_ cause premature abortion of primer extension adjacent to the alkylated G5 (highlighted in yellow) when crosslinked to their cognate aptamers. Transfected cells were incubated with different concentrations of Brc_3_preQ_1_ and Brc_3_DPQ_1_ as indicated, and primer extension was performed with isolated total RNA (primer sequence in gray). Sequencing ladders (C,T,A,G) were generated by reverse transcription of total RNA (not incubated with ligand) and addition of the corresponding dideoxynucleotides. Representative images of four (Brc_3_preQ_1_) or two (Brc_3_DPQ_1_) independent experiments are shown. Source data

The successful in vitro engineering of site-specifically crosslinked preQ_1_ and Pepper aptamers to their ligands encouraged the investigation of covalent ligand attachment in living cells. Thus, we designed two plasmids for the expression of preQ_1_ and Pepper in human cells based on the Tornado system for the production of stable circular RNA^58^. On the one hand, we inserted the Pepper aptamer sequence to generate pTornado-Pepper (Fig. 4a, left). On the other hand, we introduced both Pepper as well as the preQ_1_ aptamer separated by the F30 scaffold sequence^58^ (pTornado-preqQ_1_-Pepper; Fig. 4a, right) for tandem expression of preQ_1_ and Pepper. In this way, experiments performed to study preQ_1_ engagement (below) could be easily traced by observing Pepper fluorescence.

The plasmids were used to transfect human embryonic kidney 293T (HEK293T) cells followed by incubation with HBC530 or MsOc_3_HBC. Total RNA was extracted and subjected to 10% PAGE (Fig. 4b). For MsOc_3_HBC-treated cells expressing either Pepper or preQ_1_-Pepper, direct fluorescence detection of the gel at 530 nm revealed clear signals at the size of the circular RNAs consistent with stably attached fluorophores to the Pepper RNA that were not dislodged during RNA extraction under denaturing conditions. By contrast, no bands were visible for RNA from cells that were treated with the noncovalent HBC ligand indicating that those ligands were lost during RNA extraction (Fig. 4b, left panel). To ensure that the HBC-treated cells indeed express the circular aptamers, the gel was stained with HBC530 (Fig. 4b, middle panel). Eventually, the gel was stained with ethidium bromide to visualize total RNA (Fig. 4b, right panel).

**Extended Data Fig. 7:**
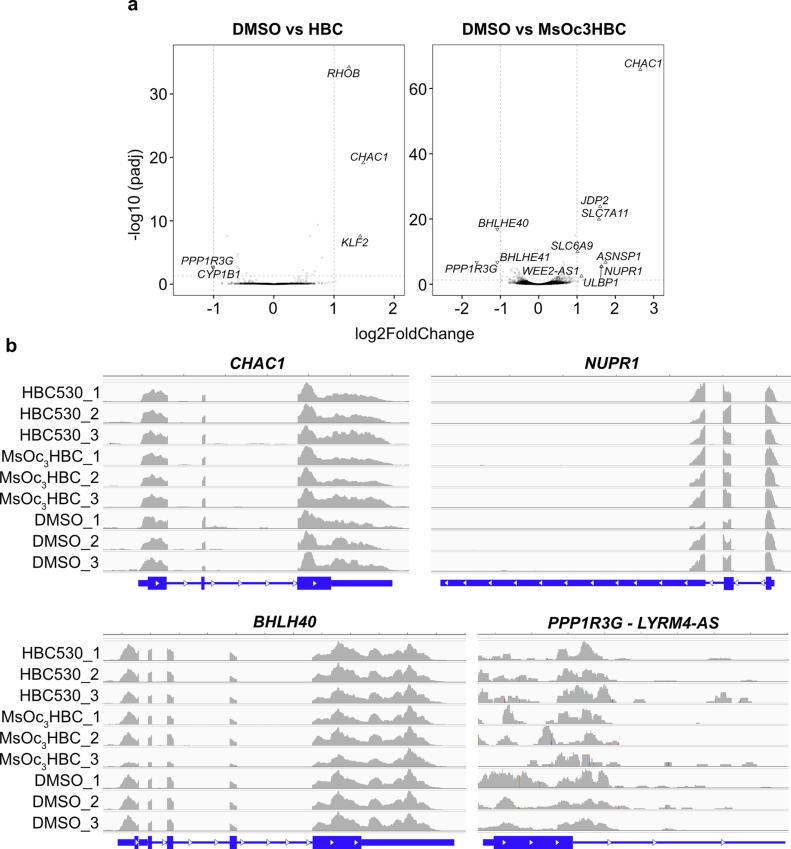
RNA-seq analysis of HEK293T cells after Pepper ligand treatment. Cells were incubated with 10 µM HBC530, MsOc_3_HBC or DMSO (control) for 5 h before RNA extraction and RNA-seq. **a**, Differential gene expression analysis indicates dysregulation (log2 fold change ≥ 1, p_adj_ ≤ 0.05; two-sided Wald test with Benjamini-Hochberg correction for multiple testing) of only five genes in HBC530 and 11 genes in MsOc_3_HBC-treated cells. **b**, Integrative Genomics Viewer (IGV) tracks of four example genes do not show premature termination of reverse transcription.

Although the absence of signal in the mock-transfected cells indicates that MsOc_3_HBC selectively crosslinks to its cognate aptamer, it is formally possible that endogenous mRNAs were also covalently modified. To investigate this possibility, we performed gene expression profiling of RNA from cells that were treated with HBC530, MsOc_3_HBC or vehicle (DMSO) reasoning that substantial alkylation should affect transcript levels in the cell. RNA sequencing (RNA-seq) data revealed only minimal perturbance of the transcriptome with 5 and 11 transcripts showing (slight) dysregulation in HBC530- and MsOc_3_HBC-treated cells, respectively (Extended Data Fig. 7a). Inspection of the corresponding IGV tracks did not show any unusual coverage bias indicative of premature termination of reverse transcription (Extended Data Fig. 7b). Thus, because most of those genes are linked to cellular stress response, their altered expression is likely due to a reaction of the cells to the treatment with the ligands rather than to their alkylation. These data indicate that our coFLAP system can be successfully used in living cells with no major adverse effects of ligand treatment on cell physiology.

We also sought to provide independent direct evidence for intracellular tethering of preQ_1_ to its aptamer. Reasoning that a covalently attached ligand should impede reverse transcription, we performed primer extension analysis on RNA extracted from HEK293T cells transfected with the preQ_1_-Pepper-Tornado construct and incubated with Brc_3_preQ_1_. Indeed, we observed premature abortion of reverse transcription in the immediate vicinity of the N7-c_3_preQ_1_ alkylated G5 of the preQ_1_ aptamer (Fig. 4c). The fact that abortion did not happen directly at G5 but 3–5 nt downstream is consistent with the still decodable Watson–Crick face of the modified nucleotide and a delayed steric clash of the bulky N7(G5)-c_3_preQ_1_ moiety in the active site pocket of reverse transcriptase. Similarly, the band pattern changed in a concentration-dependent manner when the C15U aptamer was tested with Brc_3_DPQ_1_ (Fig. 4c).

Together the results indicate that covalent tethering of both functionalized ligands to their respective aptamers occurs in a highly efficient way in the physiological environment of mammalian cells.

### Applications of the coFLAP system

**Fig. 5:**
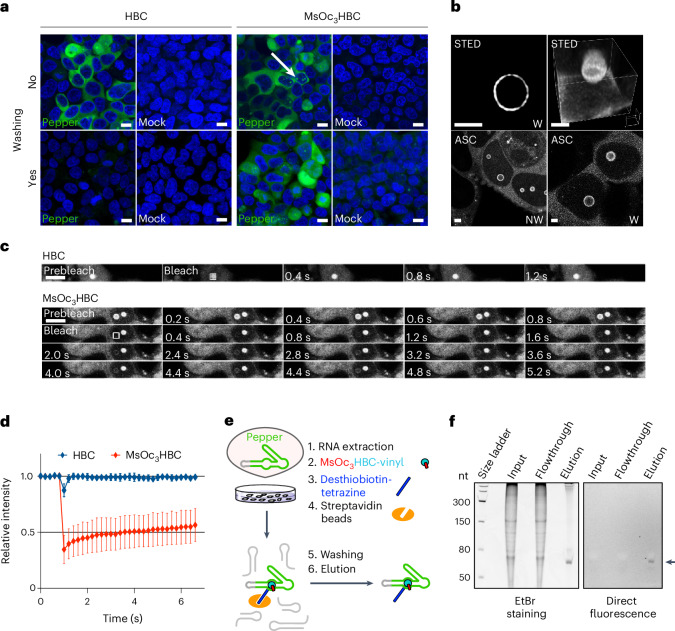
Application of coPepper for imaging and biochemical enrichment of RNA. **a**, MsOc_3_HBC elicits washout-resistant Pepper fluorescence in live-cell imaging. Top panels show cells transfected or not (Mock) with pTornado-Pepper and incubated with either HBC5 (left) or MsOc_3_HBC (right). Bottom panels, cells were washed before imaging. Green, FLAP fluorescence; blue, Hoechst staining of DNA. Arrow designates nuclear speckles. Scale bars, 10 µm. Representative images of five independent experiments. **b**, MsOc_3_HBC is suitable for super-resolution microscopy by STED (top) and Airyscan (ASC; bottom). Circular Pepper appears at the surface of nuclear spherical structures. Panels show projections of one (top left), three (bottom left) or ten (bottom right) stacks or a maximum intensity projection rendering of a complete set of stacks (top right) generated using Huygens Professional Software. Scale bars, 2 µm. Representative images for MsOc_3_HBC with (W) or without washing (NW) from two (STED) or four (Airyscan) independent experiments. **c**, coPepper is uniquely suited for FRAP analysis. FRAP was performed on cells incubated with HBC or MsOc_3_HBC. Images of nuclear speckles were acquired at 0.2-s intervals before (prebleach), during (bleach) and after photobleaching. The bleached area is designated by a square. Selected frames are shown. Three independent experiments were performed. **d**, Quantification of FRAP measurements. Mean ± s.d. of 20 different speckles is shown. Scale bar, 5 µm. **e**, The bifunctional MsOc_3_HBC-vinyl ligand enables affinity pull-down of Pepper-containing RNA. Total RNA from HEK293T cells transfected with pTornado-Pepper was incubated with MsOc_3_HBC-vinyl followed by tetrazine-mediated attachment of desthiobiotin and incubation with streptavidin beads. **f**, Aliquots (2%) of input and flowthrough and the elution fraction (50%) were separated on a 10% TBE-urea-PA gel. Pepper RNA was visualized by direct fluorescence detection (filter for *λ* 450 to 495 nm) (right panel). Total RNA was stained with EtBr (left panel). Representative images of three independent experiments are shown. Source data

Given that MsOc_3_HBC could be readily crosslinked to the Pepper aptamer (*co*Pepper) in cellulo (Fig. 4b), we reasoned that it should be particularly useful for live-cell imaging, because washing steps to reduce background should not affect the specific fluorescence signal. To test this, HEK293T cells were transfected with pTornado-Pepper and incubated with either HBC530 or MsOc_3_HBC. Both ligands caused strong predominantly cytoplasmic fluorescence in living cells. When the cells were washed, however, the signal was lost from HBC530-treated cells, while MsOc_3_HBC-treated cells robustly retained FLAP fluorescence (Fig. 5a) further demonstrating that mesylated HBC is able to attach covalently to its cognate aptamer in living cells and can provide advantages to improve signal-to-noise ratio.

Next, we examined the suitability of the coFLAP system for super-resolution microscopy. Expression of circular Pepper occasionally resulted in the appearance of distinct nuclear speckles (arrow in Fig. 5a) that we examined by super-resolution microscopy. We first used stimulated emission depletion (STED) microscopy with MsOc_3_HBC-stained live cells and obtained robust signals revealing that the circular RNA molecules are arranged around the surface of the nuclear speckles (Fig. 5b). Likewise, imaging with a ZEISS-LSM980 Airyscan 2 microscope allowed for the resolution of the ring-like structure of circular Pepper RNA in the nucleus of cells before and after washing (Fig. 5b). Moreover, we observed that fluorescent molecules joined and left these bodies (Supplementary Video 1). To assess the concentration and dynamics of Pepper RNA in these bodies, we tested whether our labeling strategy is also suitable for fluorescence correlation spectroscopy (FCS). Quantification of signals obtained in living cells indicated twofold higher fluorescence intensity in nuclear speckles compared to the signal in the cytoplasm with a mean expression of ~13 µM. We found clearly distinct dynamic behavior of the signals measured within the nuclear speckles compared to the cytoplasm. A slower moving component was enriched in speckles (35% compared to 22% in cytoplasm) displaying longer diffusion time (14 ms) and a reduced diffusion coefficient (0.7 µm^2^ s^−1^) compared to a faster moving component with a diffusion time and coefficient of 2 ms and 9.4 µm^2^ s^−1^, respectively. These data support the observation that the circular RNA molecules accumulate in defined but dynamic structures within the nucleus.

To explore further applications of the coPepper system, we hypothesized that it might be ideal for monitoring dynamic RNA localization by fluorescence recovery after photobleaching (FRAP). With a covalently linked ligand, fluorescence recovery would be exclusively attributable to the movement of the target RNA, rather than the exchange of the ligand. To test this idea, we performed FRAP measurements of Pepper RNA movement to the observed nuclear speckles. When the cells were incubated with the HBC530 ligand, photobleaching resulted in only a small decrease in signal intensity with almost instant recovery supporting the notion of fast exchange kinetics of the noncovalent ligand (Fig. 5c,d). By contrast, photobleaching was achieved in speckles of MsOc_3_HBC-treated cells, and fluorescence recovery was much slower (Fig. 5c,d) indicating that it is indeed the RNA that is monitored and not the exchange of the ligand. These results clearly show that the covalent ligand system is uniquely suited for FRAP applications to study cellular kinetics of RNA. Taken together, the experiments demonstrate that the coFLAP system shows excellent performance in a variety of imaging techniques including diffraction-limited confocal live-cell imaging, super-resolution microscopy (Airyscan and STED) as well as FCS and FRAP measurements.

Besides imaging, a covalent ligand–aptamer system might also be exploited advantageously for biochemical pull-down experiments since stringent washing protocols can be applied. At the same time, the target RNA can be followed via the inherent turn-on fluorescence. To test this, we incubated total RNA extracted from pTornado-Pepper expressing cells with the bifunctional HBC derivative MsOc_3_HBC-vinyl shown in Fig. 3c. This ligand covalently crosslinked to Pepper RNA by its MsOc_3_ handle and offered a vinyl group as second functionality that efficiently reacted with desthiobiotin-tetrazine by inverse electron demand Diels–Alder conjugation (Fig. 5e). The resulting RNA reaction mixture was incubated with streptavidin-coated magnetic beads, washed extensively and subsequently eluted with biotin. To monitor pull-down of the coFLAP, we subjected the samples to 10% PAGE and direct fluorescence detection as well as EtBr staining. Indeed, Pepper RNA was easily visualized as a single band in the eluate in both fluorescence and EtBr detection (Fig. 5f), while faint or no signals were detected in input and flowthrough fractions. Quantification of the fluorescence signals suggested a pull-down efficiency of about 22% of total Pepper RNA, while relative quantification of the bands in the EtBr-stained gel suggests about 45-fold enrichment relative to the strong band at 150 nt. However, these values are likely underestimates as quantification of faint signals such as in the input is difficult. Thus, the results clearly show that the bifunctional HBC ligand allows for substantial enrichment of the target RNA relative to total RNA on streptavidin pull-down.

### Implications for RNA drugging

**Fig. 6:**
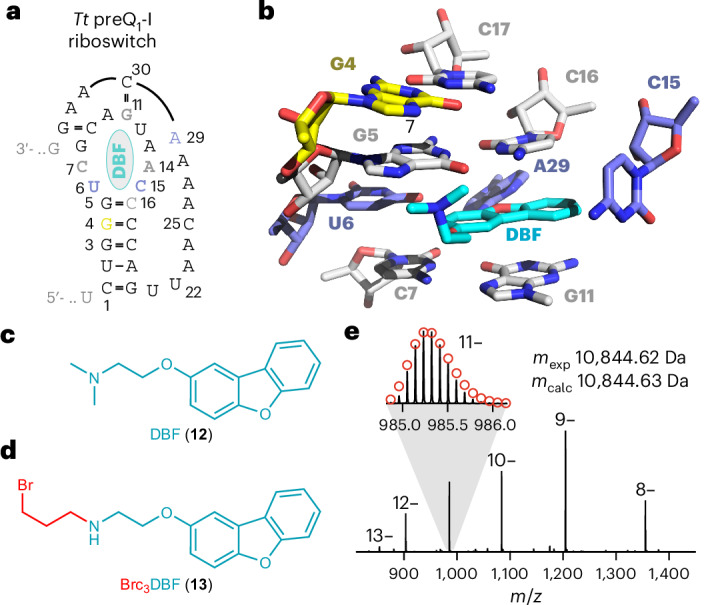
Impact on covalent RNA drug design. Drug-like small molecules that noncovalently bind to the preQ_1_ riboswitch were identified by screening strategies^17^. For one of these compounds (DBF), covalent tethering is demonstrated here. **a**, Secondary structure of the preQ_1_-I (nucleosides in gray and blue form the binding pocket; ligand (DBF) in cyan; reactive guanosine in yellow; same color code is used in **b**). **b**, Stick representation of the preQ_1_ aptamer pocket in complex with a micromolar dibenzofuran binder (DBF) (PDB 6E1U). **c**, Chemical structure of DBF. **d**, Structure-guided design of a DBF derivative with the reactive handle developed in this study (Brc_3_DBF). **e**, Covalent attachment of Brc_3_DBF incubated with the *Tt* preQ_1_ aptamer was confirmed by FT-ICR-MS analysis, and MS sequencing identified G4 as the primary site of alkylation (Extended Data Fig. 8).

**Extended Data Fig. 8:**
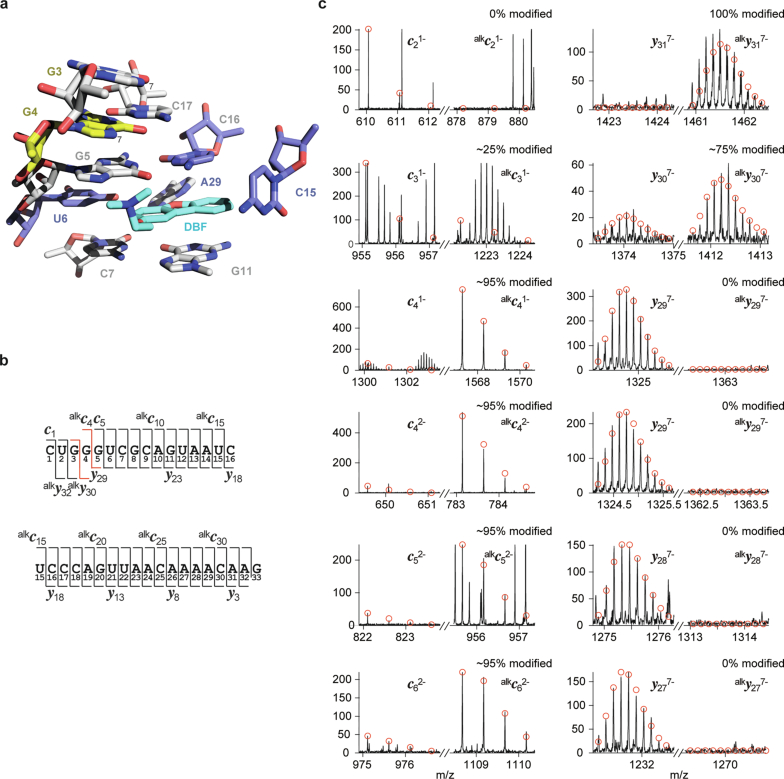
Covalent tethering of a drug-like small molecule and the preQ_1_ riboswitch. **a**, Stick representation of the preQ_1_ binding pocket (pdb code 6E1U). The ligand preQ_1_ (cyan) is in close proximity to the N7 nucleophile of G4 (yellow). **b**, FT-ICR mass spectrometric characterization of the covalent c_3_DBF–RNA complex. Collisionally activated dissociation (CAD) of (M–nH)^n–^ ions of RNA produces *c* and *y* fragment ions from RNA backbone cleavage. Fragment-ion map illustrating sequence coverage from CAD of the alkylated *Tt* preQ_1_ RNA (top). The numbering of *c* and *y* fragments starts from the 5′ and 3′ terminus, respectively. **c**, MS signals of unmodified and alkylated c_4_, c_5_ and complementary y_29_, y_28_ fragments from CAD of (M − 7H)^7−^ ions reveal G4 as the major and G3 as the minor site of alkylation (in a ratio of about 75:25); the calculated isotopic profiles for unmodified ( ~ 5% according to *c* fragments) and singly alkylated RNA are indicated by red open circles.

In light of the fundamental roles of RNAs for the regulation of gene expression and genome architecture, it is not surprising that RNAs are linked with a variety of human diseases. Therefore, the targeting of RNAs holds immense potential for the development of therapeutics^1,2,59–61^. To illustrate the possibilities of the reactive handles for covalent drug design^62^, we chose a drug-like compound that was recently found to interact with the preQ_1_ riboswitch^60^. More precisely, a dibenzofuran derivative (DBF) with no obvious structural similarity to preQ_1_ not only bound specifically and reversibly to the preQ_1_ aptamer with micromolar affinity (Fig. 6a–c)^60^, but it also modulated riboswitch activity via transcriptional termination^60^. To demonstrate the applicability of our tethering approach, we synthesized the corresponding bromopropyl-modified dibenzofuran Brc_3_DBF depicted in Fig. 6d and incubated it with the preQ_1_ RNA aptamer under near-physiological conditions. We observed that the expected c_3_DBF-linked RNA conjugate was formed, albeit at lower yield and requiring longer reaction time compared to Brc_3_DPQ_1_ (Extended Data Fig. 8a,b). FT-ICR-MS of the isolated products showed the expected signal with a 267.12-Da mass increase consistent with c_3_DBF-alkylated RNA (Fig. 6e), and top-down sequencing using CAD revealed G4 as the main site of alkylation (75%) and G3 as a minor site (25%) (Extended Data Fig. 8c). This is consistent with the expectations from structural analysis where the N7-G4 is in close proximity while N7-G3 is further away and N7-G5 is involved in hydrogen-bonding with the (protonated) secondary amine of the DBF ligand (Extended Data Fig. 8a).

The DBF-preQ_1_–RNA proof-of-principle experiment demonstrates the applicability of our tethering approach for small drug-like RNA binders, providing support for the progression of low-to-medium affinity binders from small molecule library or fragment-based screening to functional RNA inhibitors^19^. Our findings raise expectations for the development of covalent RNA drugs and contribute to the evolving field of RNA-based therapeutics.

## Discussion

In this study, a new concept for covalent tethering of small molecules with RNA aptamers has been developed through rational, structure-guided design. The synthetic hallmark of the approach is the bromo- or mesyloxy-propyl modification of the parent ligand at a site that does not interfere with the original (noncovalent) ligand–RNA recognition pattern. The short chain of only three carbon atoms is ideal for conformational integration into the binding pocket to properly position the electrophilic group for in-line attack of an RNA N7-guanine atom. This simple S_N_2-type reaction scheme is high-yielding in aqueous buffers and provides strict site specificity for the target RNA. Both bromo- and mesylate-propyl ligands allow for highly efficient covalent attachment to their cognate aptamers in cellulo. These new covalent ligand–RNA pairs are prone to improve the sensitivity and selectivity of a wide range of cellular applications. For instance, the MsOc_3_HBC-Pepper system results in robust and stable fluorescence in mammalian cells that resists washout thereby improving signal-to-background ratios. Moreover, it can be used in super-resolution microscopy applications, such as Airyscan and STED. Using both techniques with the coPepper system, we investigated the morphology and dynamics of nuclear speckles generated by the expression of circular Pepper and found that the RNA was arranged at the surface of a dynamic body rather than forming a solid speckle. This finding is highly reminiscent of a similar structure found with a circular RhoBAST aptamer that was shown to colocalize with paraspeckle components NONO and PSPC1 and suggested the induction of phase separation due to high expression levels^63^. Notably, we observed that the Pepper-induced bodies tended to disappear on prolonged microscopy, which may be caused by an increase in temperature^64^. Together with the high local concentration and low diffusion dynamics of associated circular Pepper, this supports the idea that the observed bodies represent liquid–liquid phase separated structures^63^. Most notably, we demonstrate that a FLAP system can be used to study RNA movement dynamics in the cell by FRAP. Unlike a noncovalent ligand, which shows high exchange rates and therefore makes FRAP measurements impossible, covalent tethering of the ligand to the aptamer enables the attribution of fluorescence recovery to the RNA movement rather than to ligand exchange. The ease of converting the Pepper FLAP into a coFLAP is promising for rapid dissemination of the approach for in vivo RNA imaging.

Another application of the covalent ligands is their use for the enrichment of specific RNAs, either to study the RNA itself or to investigate RNA–protein interactions. To date, the most common approaches to enrich specific RNAs include biotinylated antisense oligonucleotides or the exploitation of natural RNA–protein interaction pairs, such as MS2 coat protein and its partner RNA^65^. Recently, ultraviolet (UV) irradiation of a preQ_1_ derivative or enzymatic labeling by *E. coli* Tgt (transfer RNA guanine transglycosylase) recognizing a short hairpin motif, respectively, have been used to covalently attach biotin to the target RNA for affinity purification^17,66^. The coFLAP system combines the advantages of robust covalent linkage (that is, allowing stringent purification conditions) with easy traceability of the workflow by monitoring fluorescence. Moreover, neither in vivo nor in vitro expression of proteins, such as MS2 or Tgt, is needed. We have also demonstrated the applicability of the tethering chemistry to small molecule components targeting specific RNA structures, such as the preQ_1_ riboswitch. In light of the increasing interest in ‘drugging’ RNA^67^, covalent self-attachment of drug-like molecules might contribute to improve specificity and selectivity.

## Methods

### RNA synthesis

RNA oligonucleotides were prepared by solid-phase synthesis using phosphoramidite chemistry (2′-*O*-TOM or 2′-*O*-TBDMS protected) on controlled-pore glass solid supports^68^. RNA sequences are given in Supplementary Table 2. The c^7^G phosphoramidite for atomic mutagenesis was purchased (ChemGenes). RNA oligonucleotides were deprotected with ammonia and methylamine, followed by 1 M tetrabutylammonium fluoride in tetrahydrofuran. They were desalted (Sephadex G25) and purified by denaturing anion-exchange chromatography (Dionex DNAPac PA100, 9 × 250 mm, at 80 °C; solvent A was 25 mM Tris-HCl (pH 8.0), 20 mM NaClO_4_ in 20% aqueous acetonitrile; solvent B was 25 mM Tris-HCl (pH 8.0), 0.6 M NaClO_4_ in 20% aqueous acetonitrile). A linear gradient of 25–40% (25–45% for longer sequences) with a slope of 5% solvent B per column volume was used. Purified oligonucleotides were desalted using Sep-Pak C18 cartridges. The quality (purity and identity) of the RNAs was analyzed by AE-HPLC (Dionex DNAPac PA100, 2 × 250 mm, eluents as above) using a linear gradient of 22–35% solvent B (slope of 0.87% solvent B per column volume), followed by high-resolution electrospray ionization MS (HR-ESI-MS) (Thermo Fisher Orbitrap, negative-mode) or FT-ICR-MS (below). Measured and calculated masses are listed in Supplementary Table 2.

### Synthesis and characterization of Brc_3_DPQ_1_, Brc_3_preQ_1_, Brc_3_HBC, MsOc_3_HBC, MsOc_3_HBC-vinyl, Brc_3_DBF and Br-C4-biotin

For synthesis and analytical data, see Supplementary Note 1. NMR spectra of Brc_3_DPQ_1_, Brc_3_preQ_1_, Brc_3_HBC, MsOc_3_HBC, MsOc_3_HBC-vinyl, Brc_3_DBF and Br-C4-biotin are shown in Supplementary Figs. 1–14.

### In vitro reaction of Brc_3_ or MsOc_3_ ligands with RNA

#### preQ_1_ RNA tethering

A typical alkylation reaction was carried out in a volume of 200 μl. PreQ_1_ RNA (0.5 nmol) was dissolved in water (176 µl) and 20 µl of buffer (1 M KCl, 500 mM 2-(*N*-morpholino)ethanesulfonic acid (MES), pH 6.0). After addition of 2 µl of a 200 mM MgCl_2_ stock solution, RNA was folded by heating to 90 °C for 2 min and cooling on ice for further 2 min. The samples were incubated at 37 °C for 15 min. Then, 2 µl of ligand stock solution (12.5 mM, in H_2_O) were added. The final concentrations of the reaction mixture were: 2.5 µM RNA, 125 µM ligand, 50 mM MES, 100 mM KCl and 2 mM MgCl_2_. Samples were incubated at 37 °C for up to 48 h. After desalting of the reaction mixture using a Sep-Pak C18 cartridge, the alkylated RNA product was directly analyzed in the mixture or isolated by AE-HPLC, desalted again (Sep-Pak C18) and subjected to FT-ICR-ESI-MS (which included a further desalting step using centrifugal concentrators; below). Conversion was determined by comparing the relative ratios of the peak areas of the product and substrate.

#### Pepper RNA tethering

A typical alkylation reaction was carried out in a volume of 60 µl. Pepper RNA (0.15 nmol) was dissolved in 40 µl of water, followed by the addition of 12 µl of buffer (250 mM HEPES, 500 mM KCl, pH 7) and 6 µl of MgCl_2_ solution (20 mM). The aptamer was annealed by heating at 90 °C for 2 min and cooling on ice. Then, 2 µl of ligand stock solution (3 mM, in DMSO) was added. The final concentrations of the reaction mixture were: 2.5 µM RNA, 50 µM ligand, 50 mM HEPES, 100 mM KCl and 2 mM MgCl_2_. After incubation (37 °C, 5 h), the reaction was quenched by adding 40 µl of a Na_2_H_2_EDTA solution (200 mM) to reach a final concentration of 80 mM Na_2_H_2_EDTA in a volume of 100 µl. Each sample was analyzed by AE chromatography (Dionex DNAPac PA100 column; 4 × 250 mm) at 80 °C with a flow rate of 1 ml min^−1^. A gradient of 25–37.5% B in A in 25 min was used; Eluent A: 25 mM Tris∙HCl, 0.01 M NaClO_4_, 20% acetonitrile, pH 8.0; Eluent B: 25 mM Tris∙HCl, 0.6 M NaClO_4_, 20% acetonitrile, pH 8.0. HPLC UV traces were recorded at 260 nm.

#### Ribozyme self-biotinylation

A typical alkylation reaction was carried out in a volume of 200 µl. A 5.9 µl equivalent of 58 nt Liu ribozyme RNA stock (85.2 µM) was diluted with 142 µl of water and mixed with 20 µl of buffer (500 mM HEPES, 1000 mM KCl, pH 7.4) and 5 µl of MgCl_2_ solution (200 mM). The aptamer was annealed by heating to 90 °C for 2 min and cooling to room temperature. The mixture was adjusted to 37 °C before adding 6.7 µl of Br-C4-EG-biotin ligand stock solution (75 mM, in DMSO). The final concentrations of the reaction mixture were: 2.5 µM RNA, 2.5 mM ligand, 50 mM HEPES, 100 mM KCl and 5 mM MgCl_2_. After a desired interval at 37 °C the reaction was quenched by adding 200 µl of a aqueous urea solution (12 M). A 100-µl portion of each sample was analyzed by reversed phase chromatography (Supelcosil LC-18-S column; 4.6 × 250 mm, 5 µm) at 40 °C with a flow rate of 1 ml min^−1^. A gradient of 0–20% B in A in 40 min was used; Eluent A: 50 mM triethylammonium acetate pH 7.0; B: acetonitrile. HPLC UV traces were recorded at 260 nm.

### FT-ICR mass spectrometric analysis of RNA alkylation products

Methanol was HPLC grade (Acros), ammonium acetate (≥99.0%, Na ≤5 mg kg^−1^, K ≤5 mg kg^−1^), piperidine (≥99.5%) and imidazole (≥99.5%, Na ≤50 mg kg^−1^, K ≤50 mg kg^−1^) were from Sigma-Aldrich, and H_2_O was purified to 18 MΩ cm^−1^ at room temperature using a Milli-Q system (Millipore). For desalting before ESI, 400 µl of an ammonium acetate solution (100 mM in H_2_O) was added to 100 µl RNA solution (~1 nmol in H_2_O) and concentrated to 100 µl using Vivaspin 500 centrifugal concentrators. The process was repeated 5–7 times, followed by 6–7 cycles of concentration and dilution with H_2_O. RNA concentration was determined by UV absorption at 260 nm using a NanoPhotometer (Implen). Experiments were conducted using a 7-Tesla FT-ICR mass spectrometer (Bruker APEX Ultra). With broadband detection (2 M data points for a roughly 2-s transient), the mass resolving power of this instrument is routinely roughly 220,000, 120,000 and 80,000 at *m/z* 500, 1,000 and 1,500, respectively, and the mass accuracy is around 1 ppm with internal calibration and roughly 20 ppm with external calibration. RNA (M–nH)^n−^ ions were generated by ESI (flow rate 1.5 µl min^−1^) from 1–2 µM solutions in 1:1 or 9:1 H_2_O/CH_3_OH vol/vol with piperidine (2–10 mM) and imidazole (0–10 mM) as additives, isolated in a linear quadrupole, and dissociated by CAD in a collision cell through which a flow of Ar gas was maintained; for a more detailed description of the experimental setup, see ref. ^69^. Between 25 and 500 scans were added for each spectrum (20–50 for ESI, 100–500 for CAD), and data reduction used the SNAP2 algorithm (Compass Apex Control v.3.0.0, Bruker Daltonics).

### Kinetics assays of tethering reactions

RNA was alkylated as described above (on a 1.5-nmol scale; 600-µl reaction volume, 2.5 µM RNA concentration). At indicated time points, 50-μl aliquots were removed and quenched immediately by addition of 50 μl of stop solution (12 M urea). The samples were analyzed by AE-HPLC and UV-detection. Using OriginPro (2020) software, conversion versus time data were fitted to (fraction reacted) =*k*_1_*(k*_1_ + *k*_2_)^−1^(1 − e^−(*k*1+*k2*)*t*^) in which *k*_1_ corresponds to the alkylation rate. Three independent replicates were generated for all kinetic assays.

To evaluate the pH dependence of the alkylation reaction, the following buffers were used: 50 mM sodium acetate (pH 3.5, 4.0, 4.5, 5.0 and 5.5); 50 mM MES (pH 6.0 and 6.5) and 50 mM MOPS (pH 7.0 and 7.5) all of which contained 1 M KCl. The *k*_1_ versus pH data were fitted to *k*_1_ = *k*_max_(1 + 10^*n*(*p*K*a-*pH)^)^−1^.

A typical Michaelis–Menten analysis was carried out in a volume of 12.5 ml. preQ_1_ RNA (0.25 nmol) was dissolved in water (11.1 ml) and 1.25 ml buffer (100 mM KCl, 500 mM MES, pH 6.0). Samples were refolded by heating to 90 °C for 2 min and cooling on ice for further 2 min. The samples were incubated at 37 °C for 1 h. Then, 1–5 µl of the ligand stock solution (12.5 or 1.25 mM) were added to achieve the desired concentrations. On mixing, the samples were incubated at 37 °C. Samples were generally too dilute for direct HPLC analysis. Thus, the reactions were quenched at indicated time points by freezing in liquid nitrogen. After lyophilization the residue was dissolved in 8 M urea solution (500 µl) and concentrated by Vivaspin centrifugal concentrators (molecular weight cutoff of 3,000) to a volume of approximately 100 µl. Then, 500 µl of 8 M urea solution was added and the solution was spun down to 100 µl again. The process was repeated twice. The remaining RNA solution was adjusted to 100 µl by addition of 8 M urea and subjected to HPLC analysis.

### Kinetics assay of ligand recognition (2-aminopurine fluorescence)

2-Aminopurine-labeled preQ_1_ RNA was dissolved in 107 µl of water. Then, 12.5 µl of buffer (100 mM KCl, 50 mM MOPS, pH 7.5) and 1.2 µl of MgCl_2_ solution (2 mM) were added. Samples were refolded by heating to 90 °C for 2 min and cooling on ice for further 2 min. Four such samples were prepared and subsequently transferred to Quartz cuvettes (1-cm pathway). A dilution series of four different ligand concentrations was generated (100, 200, 500 and 1,000 µM in H_2_O). Fluorescence measurements were performed at 25 °C on a Cary Eclipse fluorescence spectrometer equipped with a Peltier element. The RNA samples were allowed to pre-equilibrate at 25 °C for 15 min before the measurement. The change in fluorescent signal on addition of one of the ligand dilutions (1.2 µl, manual addition and mixing) was monitored over the course of 350 s. Entry and exit slit widths were 10 nm; 308 and 372 nm were chosen as the excitation and emission wavelengths, respectively. Detector voltage was 600 V. Measurements were performed in triplicate. The change in fluorescence signal was plotted versus time and fitted to *k*_obs_ = *A*(1 − e^−*kt*^). The *k*_obs_ values were plotted against ligand concentration and linear regression to *k*_obs_ = *k*_on_ *×* *c*_*L*_ + *k*_off_ provided on and off rates. The dissociation constant of the RNA–ligand complex was estimated by the quotient of *k*_off_ and *k*_on_. Data analysis was performed in Origin 2020 (OriginLab).

### Comparison of ribozyme alkylation chemistries

To examine self-biotinylation properties of different systems, the respective RNAs (Supplementary Table 2) were generated by in vitro transcription using the HiScribe T7 High Yield RNA Synthesis Kit according to the manufacturer’s instructions followed by phenol-chloroform extraction (ROTI phenol/chloroform/isoamylalkohol) and EtOH/NaOAc (pH 5.2) precipitation. The purified RNA was incubated at 2.5 µM (final concentration) with 1 mM or 50 µM of the respective ligands for 5 or 16 h at 37 °C in reaction buffer (50 mM HEPES-KOH pH 7.4, 100 mM KCl, 5 mM MgCl_2_) in a volume of 10 µl. Comparison of different ligands with the 58-nt ribozyme^57^ was carried out for 5 h with 1 mM ligand. The reactions were stopped by addition of 80 mM EDTA, mixed with 12 µl of 2× RNA Loading Dye and incubated for 2 min at 90 °C. After cool-down on ice, the samples were loaded onto a 10% Tris-borate-EDTA (TBE)-polyacrylamide (19:1 polyacrylamide:bisacrylamide) gel containing 7 M urea and electrophoresed at 180 V. The gel was stained with ethidium bromide and imaged under UV light in a ChemiDoc Imaging System (Bio-Rad). Then the RNA was blotted onto a Hybond-N+ membrane (Amersham) using a semidry blotting system (Bio-Rad). Transferred RNAs were UV crosslinked to the membrane, followed by blocking in High-SDS solution (50 mM Na-phosphate buffer pH 7.0, 0.75 M NaCl, 75 mM Na_3_-citrate, 0.1% lauroylsarkosin, 50% deionized formamide, 7% sodium dodecylsulfate, 2% Blocking Reagent) for 30 min at room temperature followed by incubation for 30 min at room temperature in the dark with 1.67 µg ml^−1^ AlexaFluor 647-streptavidin in Imaging Buffer (40 mM HEPES-KOH pH 7.5, 100 mM KCl, 5 mM MgCl_2_). The membrane was washed with Imaging Buffer and the signal was detected using a Typhoon FLA 9500 instrument (Cytiva).

### Riboswitch activity assay in *E. coli*

Translational repression activity of Brc_3_preQ_1_ and Brc_3_DPQ_1_ was tested in bacteria in comparison with the WT preQ_1_ ligand^47^. Briefly, a pQE70 construct containing the WT *Tt* preQ_1_ aptamer upstream of an eGFP reporter gene (pQE70-RS-I-C15) was subjected to site-directed mutagenesis using the Q5 Site-Directed Mutagenesis Kit to introduce the C15U mutation (pQE70-RS-1-C15U). *E. coli* JW0434 (ref. ^54^) containing a deletion of the *queC* gene, which abolishes endogenous preQ_1_ synthesis, were transformed with the WT or mutated aptamer constructs. Bacteria were grown at 37 °C in Luria-Bertani (LB) media (1% tryptone, 0.5% yeast extract, 10 mM MgSO_4_, 0.5% NaCl, pH 7) until an optical density at 600 nm of ~0.5 and reporter transcription was induced by addition of 0.8 mM isopropyl-β-d-thiogalactopyranoside. Concomitantly, 1 mM of the respective modified ligands was added and after 6 h, GFP fluorescence was measured in a CLARIOstar Plus (BMG Labtech) plate reader^47^. Intensity values were corrected for background fluorescence of LB medium and normalized to the cell density determined at 600 nm (ref. ^47^). Translation inhibition was calculated relative to mock-treated (water) control cultures. Three experiments with three technical replicates each were performed.

### In vivo tethering with Brc_3_ and MsOc_3_ ligands

#### DNA constructs

To generate expression constructs for circular Pepper RNA, a 49-nt Pepper sequence (Supplementary Table 2) with *Not*I/*Sac*II overhangs was synthesized (Eurofins) and cloned into *Not*I/*Sac*II: digested pAV-U6+27-Tornado-Broccoli (Addgene no. 124362)^58^ to replace Broccoli resulting in pTornado-Pepper. Likewise, pTornado-preQ_1_-Pepper was generated by synthesizing a double-stranded DNA (dsDNA) fragment comprising the sequence between *Not*I/*Sac*II restrictions sites in pAV-U6+27-Tornado-F30-Broccoli-aptNFkB#5 (Addgene no. 124362)^58^, except that Broccoli was replaced by the preQ_1_ sequence from *T. tencongensis* (Supplementary Table 2) and aptNFkB#5 was replaced by the Pepper sequence followed by cloning into the *Not*I/*Sac*II: digested pAV-U6+27-Tornado-F30-Broccoli-aptNFkB#5 vector. Additionally, a mutated version of pTornado-preQ_1_-Pepper was generated using the Q5 Site-Directed Mutagenesis Kit to introduce the C15U mutation (pTornado-preQ_1_C15U-Pepper).

#### Cell culture and transfection

HEK293T cells were maintained in DMEM-12 growth medium supplemented with 1× Glutamax and 10% fetal bovine serum at 37 °C and 5% CO_2_. Transfection of pTornado-Pepper and pTornado-preQ_1_-Pepper was performed using Lipofectamine2000 according to the manufacturer’s instructions using 2.5 µg of DNA and 7.5 µl of transfection reagent in 150 µl of OptiMEM medium.

#### Gel-based fluorescence detection

Transfected cells were incubated with 10 µM MsOc_3_HBC or HBC530 for 5 h in cell growth medium, and total RNA was isolated using TRI Reagent, DNA was removed by DNase I digestion. RNA was reextracted by acid ROTI aqua-phenol-chloroform, precipitated and dissolved in water. The samples (5–10 µg) were mixed with 2× RNA loading dye, denatured for 2 min at 90 °C and electrophoresed at 180 V for 1 h on an 8% TBE-polyacrylamide (19:1 polyacrylamide:bisacrylamide) gel containing 7 M urea. HBC530 or MsOc_3_HBC fluorescence was detected with Alexa488 settings in a Typhoon FLA 9500 instrument (Cytiva). Subsequently, the gel was stained with 10 µM HBC530 in Imaging Buffer (40 mM HEPES pH 7.5, 100 mM KCl, 5 mM MgCl_2_) for 10–15 min and again subjected to Typhoon detection. Finally, the gel was stained with ethidium bromide and imaged in a ChemiDoc Imaging System (Bio-Rad).

#### Primer extension analysis

HEK293T cells were transfected with pTornado-preQ_1_-Pepper or pTornado-preQ_1_C15U-Pepper for 3 days and incubated with 50 and 100 µM Brc_3_preQ_1_ or 100 and 200 µM Brc_3_DPQ_1_ ligand for 5 h. Total RNA was isolated and 20 µg of RNA was mixed with 16 pmol Alexa647-labeled PE DNA primer in a volume of 12 µl, heated to 90 °C for 2 min, 65 °C for 5 min and 50 °C for 5 min, and cooled on ice for 1 min. The sample was mixed with 4 µl of 5× SuperScript IV RT buffer, 1 µl of 100 mM 1,4-dithiothreitol, 1 µl of 5 mM dNTPs (1.25 mM of each dNTP), 2 µl of dimethyl sulfoxide and 0.4 µl of SuperScript IV reverse transcriptase (200 U µl^−1^, Invitrogen), incubated for 25 min at 55 °C followed by reaction stop with 1 µl of 4 M NaOH and incubation at 90 °C for 5 min. After cooling on ice, complementary DNA was precipitated with 90 µl of precipitation mix (650 µl of water, 150 µl of 1 M NaOAc pH 5.2, 250 µl of ethanol, 10 µl of 20 mg ml^−1^ glycogen) for 30 min on ice. The pellet was dissolved in 8 µl of loading dye (97% formamide, 10 mM EDTA) and electrophoresed in a 10% TBE-urea-PAGE at 35 W (ref. ^70^). The dried gel was scanned in a Typhoon FLA 9500 instrument (Cytiva). A sequencing ladder was generated from the same RNA (not incubated with ligand) by addition of 1.5 µl of 10 mM dideoxynucleotide (G, A, T or C) to the RNA–primer mix.

#### Live-cell imaging

Cells were seeded onto eight-well chamber slides and transfected with pTornado-Pepper. After 3 days, cells were incubated with 10 µM MsOc_3_HBC or HBC530 for 5 h in cell growth medium. Then, 1 µg ml^−1^ Hoechst33342 was added to stain DNA. Cells were either imaged directly or subjected to three washes with PBS before imaging. Live-cell images were obtained using a Leica SP8 microscope (Leica Microsystems) equipped for live-cell imaging (37 °C, 5% CO_2_, humidified atmosphere) with HC PL APO CS2 ×93/1.30 GLYC objective. Pepper fluorescence was imaged using pulsed white light laser (WLL) with excitation at 485 nm and emission filter at 495–585 nm. Hoechst-stained nuclei were imaged using Diode 405 laser with excitation at 405 nm and emission filter at 415–475 nm. Images were recorded using *Z*-step stack and sequential scan. Images were processed by ImageJ (v.1.53c) software. For stack analysis, the ‘Sum Slices’ projection type was used.

#### Airyscan confocal microscopy

Living cells prepared as described above were imaged on a Zeiss LSM 980 Airyscan 2 microscope (Carl Zeiss Microscopy) equipped for live-cell imaging (37 °C, 5% CO_2_, humidified atmosphere) using a C-Apochromat ×40/1.2 W autocorr M27 water immersion objective using 0.4% 488-nm laser intensity with an effective pixel size of 50 nm and *z*-stacks obtained with a spacing of 200 nm. The raw data files containing the signal of all 32 detectors were then used for deconvolution using Huygens Professional v.24.04.0p2 software (Scientific Volume Imaging). Time series were acquired using the definite focus system at 0.56-s intervals.

#### STED

STED microscopy was performed on a Leica SP8 gSTED microscope (Leica Microsystems) equipped for live-cell imaging (37 °C, 5%CO, humidified atmosphere) using a HC PL APO ×93/1,30 GLYC motCORR STED WHITE immersion objective. Images were acquired using the WLL (set at 70% intensity) line at 478 nm set at 60% and the 592-nm depletion laser (set at 83% intensity) and used with 22% for imaging using an effective pixel size of 20 nm in two dimensions or 40 nm in three dimensions. Signals were recorded with a scan speed of 400 Hz, and a time gate of 10 ns from 483 to 582 nm using a HyD camera. Image stacks obtained with a *z* axis resolution of 60 nm were deconvolved using Huygens Professional Software.

#### FCS

Cells were prepared as above and imaged on a Zeiss-LSM980 Airyscan 2 microscope using a C-Apochromat ×40/1.2 W autocorr M27 water immersion objective with a correction collar. For FCS measurements, the Dynamics Profiler (Zeiss) module was used and up to nine spots measured for 10 s at a 488-nm laser intensity of 4% and a camera gain of 875 V. Due to initial bleaching, the signals of the first frames (~1 s) were excluded and the remaining signal corrected for bleaching using detrending set at 500 ms. The autocorrelation curves were then fitted using a two-component 3D model to calculate concentration, diffusion time and the diffusion coefficient (Zeiss ZEN v.3.8).

#### FRAP experiments

Cells were prepared as described above and imaged on a Leica SP8 confocal laser scanning microscope using the WLL line at 478 nm and the PMT camera set at 875 V to detect signals from 498 to 567 nm. For FRAP measurements, 512 × 75 pixel images were recorded at an effective pixel size of 81.54 nm and a pixel dwell time of 3.16 µs. Because of strong bleaching during the first five to ten frames, 15 prebleach frames were recorded at a time interval of 0.19 to 0.2 s followed by a single bleach frame of an area encompassing the circular structure in the nucleus (~250 pixel^2^) with a 488-nm argon laser intensity set to 100% in zoom-in mode. After bleaching, signals were recorded for 30 consecutive frames over a period of 5.85 s. Quantification of signal intensities was performed using Metamorph v.7.8 software (Molecular Devices) in the bleaching area, the area of the cell around the bleaching area (‘whole cell’), as well as a background area. After background subtraction, signals were corrected for bleaching and loss of fluorescence with respect to signals outside the target area and normalized to the mean signal intensity obtained from the five frames immediately preceding the bleaching pulse.

### RNA-seq and data analysis

HEK293T cells were incubated with 10 µM HBC530, 10 µM MsOc_3_HBC or DMSO for 5 h before total RNA was extracted using the innuPREP RNA Mini Kit 2.0. RNA was incubated with DNAseI and purified by phenol and chloroform extraction and isopropanol precipitation. Poly(A) RNA selection, library preparation and sequencing of three biological replicates was performed by Azenta Life Sciences with 30 million paired-end reads on an Illumina NovaSeq XPlus instrument. Sequencing reads were trimmed using Trimmomatic v.0.36 and mapped to the *Homo sapiens* GRCh38/hg38 reference genome using STAR aligner v.2.5.2b. Unique gene hit counts were calculated using featureCounts from the Subread package v.1.5.2. Differential gene expression analysis was performed by DESeq2, and genes with adjusted *P* value < 0.5 (Wald test with Benjamini–Hochberg correction for multiple testing) and log_2_ fold change >1 were considered differentially expressed. Volcano plots were generated in RStudio (R v.4.2.2), and manual inspection of read coverage was done using the IGV tool (v.2.17.4).

### Streptavidin pull-down experiments

Total RNA was isolated from HEK293T cells after 3 days of transfection with pTornado-Pepper using TRI Reagent according to the manufacturer’s instructions, dissolved in 50 mM HEPES, 100 mM KCl, 2 mM MgCl_2_, pH 7, heated to 90 °C for 2 min and subsequently transferred to room temperature for 10 min to allow refolding. Then, 100 µg total RNA were incubated with 200 µM Msc_3_HBC-vinyl ligand in a total volume of 50 µl and incubated at 37 °C for 5 h. To remove unreacted ligand, RNA was precipitated by the addition of 30 µl of 3 M NaOAc (pH 5.2), 2.5 µl of 5 mg ml^−1^ glycogen and 260 µl of ethanol. The pellet was washed with 70% ethanol, air dried and dissolved in 10 µl of water. For biotinylation, 15 µl of 5 mg ml^−1^ desthiobiotin-tetrazine (DTB-TET) and 25 µl of 12 M urea were added and incubated overnight at room temperature in a total volume of 50 µl. RNA was precipitated as above, dissolved in 250 µl of water and subjected to Vivaspin centrifugal concentrators (molecular weight cutoff of 3,000) to remove excess DTB-TET. Then, 250 µl purified RNA were added to 250 µl of Streptavidin Magnetic Beads (slurry (200 µl volume of beads) equilibrated in 2× B&W buffer (10 mM Tris-HCl, pH 7.5, 1 mM EDTA, 2 M NaCl) according to the manufacturer’s instructions and incubated at room temperature for 30 min with gentle agitation followed by two washes with 1× B&W buffer. Elution was carried out by incubation of the beads for 30 min at 37 °C with 200 µl of elution buffer (20 mM Tris-HCl, pH 7.5, 50 mM NaCl, 4 mM biotin) and subsequent RNA precipitation as above. Input, flowthrough (2.5% each) and eluate (50%) were separated on a 10% TBE-urea polyacrylamide gel. Direct fluorescence was detected in a ChemiDoc Imaging System (Bio-Rad) using a Blue Sample Tray (Bio-Rad) (filter for *λ* 450 to 495 nm). Subsequently, the gel was stained with ethidium bromide and imaged under UV light.

Source information of key reagents and kits is provided in Supplementary Table 3.

### Reporting summary

Further information on research design is available in the Nature Portfolio Reporting Summary linked to this article.

## Online content

Any methods, additional references, Nature Portfolio reporting summaries, source data, extended data, supplementary information, acknowledgements, peer review information; details of author contributions and competing interests; and statements of data and code availability are available at 10.1038/s41589-024-01801-3.

## Supplementary information

Supplementary Information Supplementary Note 1, Tables 1 and 2 and Figs. 1–14.

Reporting Summary

Supplementary Video 1 Movement of pepper signals to and from spherical structures in the nucleus. HEK293T cells transiently transfected with pTornado-Pepper and incubated with MsOc_3_HBC for 5 h were imaged on a Zeiss LSM Airyscan 2 microscope using a C-Apochromat ×40/1.2 W autocorr M27 water immersion objective with a correction collar. Pixel size, 50 nm; time interval between frames, 0.5 s. Images were cropped and subjected to deconvolution using Huygens software.

Supplementary Table 3 Source information for key reagents and kits.

## Source data

Source Data Figs. 4 and 5 and Extended Data Figs. 5 and 6 Source Data for Figs. 4 and 5 and Extended Data Figs. 5 and 6.

Source Data Fig. 1 Source data for Fig. 1.

Source Data Fig. 2 Source data for Fig. 2.

Source Data Fig. 3 Source data for Fig. 3.

Source Data Extended Data Fig. 1 Source data for Extended Data Fig. 1.

Source Data Extended Data Fig. 2 Source data for Extended Data Fig. 2.

Source Data Extended Data Fig. 3 Source data for Extended Data Fig. 3.

Source Data Extended Data Fig. 4 Source data for Extended Data Fig. 4.

Source Data Extended Data Fig. 6 Source data for Extended Data Fig. 6.

## Data Availability

RNA-seq data are available at the Gene Expression Omnibus repository under accession number GSE271728. Source data are provided with this paper.
