# Supplementary material for: Engineering covalent small molecule–RNA complexes in living cells
Source: Nat Chem Biol. 2025 Jan 6;21(6):843–54. doi: 10.1038/s41589-024-01801-3 (PMC12122380; doi:10.1038/s41589-024-01801-3)
Supplement: Supplementary file 1 — Supplementary Note 1, Tables 1 and 2 and Figs. 1–14. [file 41589_2024_1801_MOESM1_ESM.pdf]

nature chemical biology

Supplementary information https://doi.org/10.1038/s41589-024-01801-3

Engineering covalent small molecule–RNA complexes in living cells

In the format provided by the authors and unedited

Contents

Supplementary Note 1 2

General 2
Synthesis of Brc_3DPQ_1 2
Synthesis of Brc_3HBC 3
Synthesis of $MsOc_3HBC$ 3
Synthesis of $MsOc_3HBC$-vinyl 3
Synthesis of Brc_3DBF 4
Synthesis of Br-C4-EG-biotin 5

Supplementary Tables

Supplementary Table 1 6
Supplementary Table 2 7

Supplementary Figures

Supplementary Figure 1 9
Supplementary Figure 2 10
Supplementary Figure 3 11
Supplementary Figure 4 12
Supplementary Figure 5 13
Supplementary Figure 6 14
Supplementary Figure 7 15
Supplementary Figure 8 16
Supplementary Figure 9 17
Supplementary Figure 10 18
Supplementary Figure 11 19
Supplementary Figure 12 20
Supplementary Figure 13 21
Supplementary Figure 14 22

Supplementary Note

General

Chemical reagents and solvents were purchased from commercial suppliers (Sigma-Aldrich, Biosynth, abcr) and used without further purification. Dry solvents were used for all non-aqueous reactions, which were carried out under argon atmosphere. Analytical thin-layer chromatography (TLC) was performed on Marchery-Nagel Polygram SIL G/UV254 plates. Silica gel 60 (70–230 mesh) was used for flash column chromatography. ^{1}H, and ^{13}C NMR spectra were recorded on *Bruker DRX 300 MHz*, *Bruker Avance 4 Neo 400 MHz*, and *Bruker Avance 4 Neo 700 MHz* instruments. Chemical shifts (δ) are reported relative to tetramethylsilane (TMS) and referenced to the residual proton or carbon signal of the deuterated solvent: DMSO-d_6 (2.50 ppm), Methanol-d_4 (3.31) for ^{1}H NMR; DMSO-d_6 (39.52 ppm), Methanol-d_4 (49.00) for ^{13}C NMR spectra. ^{1}H and ^{13}C assignments are based on COSY, HSQC, and HMBC experiments. ESI-MS experiments were performed on a Thermo Fisher QExactive Classic. Samples were analyzed in the positive-ion mode."

Synthesis of Brc$_3$DPQ$_1$ (as dihydrobromide salt)

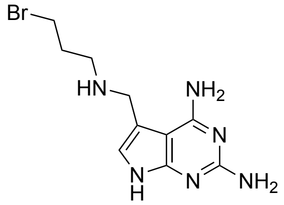

Brc$_3$DPQ$_1$ (**1**)

7-(*N*-((2'-Hydroxyethyl)aminomethyl)-7-deaza-2,6-diaminopurine (trifluoroacetate salt) (100 mg, 285 µmol; prepared according to ref. [1]) was dissolved aqueous hydrobromic acid (65 %, 1.5 mL) and heated at 80 °C for 48 h. The volatiles were removed under reduced pressure to give Brc^{3}DPQ$_1$ (dihydrobromide salt). Yield: 130 mg of Brc$_3$DPQ$_1$ as a light-brown solid (98%). TLC: *n*-butanol / acetic acid / water 2:1:1, R_f : 0.60. HR-ESI-MS (m/z): $[M+H]^+$ found: 285.0457; $[M+H]^+$ calculated: 285.0458. ^{1}H-NMR (400 MHz, DMSO-d_6, 25 °C): δ 11.97 (d, J_{HH} = 2.2 Hz, 1H, ***H***N(9)), 8.95 (bs, 2H, ***H*₂**N$^+$), 8.19 (s, 2H, ***H*₂**N(6)), 7.30 (bs, 2H, ***H*₂**N(2)), (d, J_{HH} = 2.3 Hz, 1H, ***H***C(8)), 4.41 (m, 2H, ***H*₂**CC(7')), 3.73 (t, 2H, J_{HH} = 7.0 Hz, ***H*₂**C(2')), 3.43 (m, 2H, ***H*₂**C(1')) ppm. ^{13}C-NMR (100 MHz, DMSO-d_6): δ 153.0 & 151.2 ***C***(2) & ***C***(4) & ***C***(6), 124.2 ***C***(7), 106.2 ***C***(8), 93.5 ***C***(5), 47.0 ***C***(1'), 41.5 H_2***C***C(7), 26.4 ***C***(2') ppm. For NMR spectra see Supplementary Figures 1-3.

Synthesis of Brc$_3$HBC

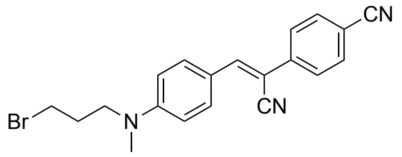

Brc$_3$HBC (**9**)

[4-((3-Hydroxypropyl)(methyl)amino)-benzylidene]-4-cyanophenylacetonitrile (105 mg, 331 µmol; prepared according to ref. [2]) was dissolved in dichloromethane (2.0 mL) and cooled to 0 °C under argon atmosphere. Then, triphenylphosphine (PPh_3, 130 mg, 496 µmol) and carbon tetrabromide (CBr_4, 165 mg, 496 µmol) were added and stirred at room temperature. After two hours reaction time, the entire mixture was loaded on a silica gel column and eluted with 100% dichloromethane. Yield: 104 mg of Brc$_3$HBC as an orange solid (83%). HR-ESI-MS (m/z): $[M+H]^+$ calcd.: 380.08, found: 380.08. ^{1}H-NMR:

(400 MHz, DMSO-d_6, 25 °C): 2.08 (2H, p, *J* = 6.88 Hz, $BrCH_2C\boldsymbol{H_2}CH_2N$), 3.04 (3H, s, $C\boldsymbol{H_3}$), 3.57 (4H, m, $BrC\boldsymbol{H_2}CH_2C\boldsymbol{H_2}N$), 6.85 (2H, d, *J* = 9.15 Hz, C***H*** (11&13)), 7.84 – 7.93 (6H, m, C***H*** (2&6, 3&5, 10&14)), 8.01 (1H, s, C***H*** (8)). ^{13}C-NMR: (400 MHz, DMSO-d_6, 25 °C): δ = 29.66 ($BrCH_2\boldsymbol{C}H_2CH_2N$), 32.17 ($Br\boldsymbol{C}H_2CH_2CH_2N$), 38.10 ($\boldsymbol{C}H_3$), 49.86 ($BrCH_2CH_2\boldsymbol{C}H_2N$), 100.16 ***C***(7), 109.71 ***C***(1), 111.52 ***C***(11&13), 118.69 & 118.78 (2x***C***N), 120.38 ***C***(9), 125.47 ***C***(3&5), 131.99 ***C***(10&14), 132.89 ***C***(2&6), 139.56 ***C***(4), 145.38 ***C***(8), 151.11 ***C***(12). For NMR spectra see Supplementary Figures 4,5.

Synthesis of MsOc$_3$HBC

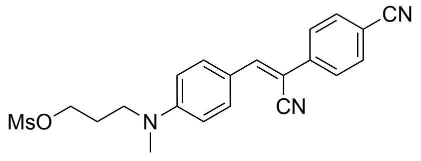

MsOc$_3$HBC (**10**)

[4-((3-Hydroxypropyl)(methyl)amino)-benzylidene]-4-cyanophenylacetonitrile (51.0 mg, 161 µmol; prepared according to ref. [2]), triethylamine (NEt_3, 48.8 mg, 67.2 µL, 482 µmol) and methanesulfonylchloride (MsCl, 27.6 mg, 18.7 µL, 241 µmol) were dissolved in dichloromethane (4.0 mL) and stirred overnight at room temperature. After reaction control and 100% consumption of the starting material, the entire mixture was poured on a silica gel column and the product was eluted using 0 - 1% methanol in dichloromethane. Yield: 56.0 mg of MsOc$_3$HBC as an orange solid (88%). HR-ESI-MS (m/z): $[M+H]^+$ calcd.: 396.14, found: 396.14. ^{1}H-NMR: (400 MHz, $CDCl_3$, 25 °C): 2.09 (2H, p, *J* = 6.40, ($MsOCH_2C\boldsymbol{H_2}CH_2NR_2$), 3.03 (3H, s, S-C***H₃***), 3.09 (3H, s, N-C***H₃***), 3.60 (2H, t, *J* = 6.96, $MsOCH_2CH_2C\boldsymbol{H_2}NR_2$), 4.30 (2H, t, *J* = 5.84, $MsOC\boldsymbol{H_2}CH_2CH_2NR_2$), 6.75 (2H, d, *J* = 8.94 Hz, C***H*** (11&13)), 7.48 (1H, s, C***H*** (8)). 7.70 (4H, q, *J* = 8.94 Hz, C***H*** (2&6, 3&5), 7.88 (2H, d, *J* = 8.97 Hz, C***H*** (10&14)). ^{13}C-NMR: (100 MHz, $CDCl_3$, 25 °C): δ = 27.06 ($MsOCH_2\boldsymbol{C}H_2CH_2NR_2$), 37.62 (S-$\boldsymbol{C}H_3$), 38.80 (N-$\boldsymbol{C}H_3$), 48.56 ($MsOCH_2CH_2\boldsymbol{C}H_2NR_2$), 67.32 ($MsO\boldsymbol{C}H_2CH_2CH_2NR_2$), 102.72 ***C***(7), 111.22 ***C***(1), 111.74 ***C***(11&13), 118.75 (2x***C***N), 121.31 ***C***(9), 125.86 ***C***(3&5), 132.31 ***C***(10&14), 132.79 ***C***(2&6), 140.10 ***C***(4), 144.71 ***C***(8), 151.15 ***C***(12). For NMR spectra see Supplementary Figures 6,7.

Synthesis of MsOc$_3$HBC-vinyl

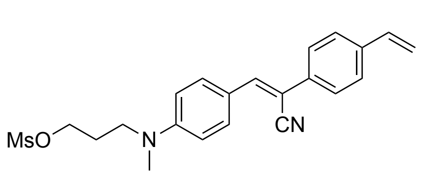

MsOc$_3$HBC-vinyl (**10**)

[4-((3-Hydroxypropyl)(methyl)amino)-benzylidene]-4-vinyl-phenylacetonitrile (274 mg, 860 µmol; prepared according to ref. [2]), triethylamine (NEt_3, 261 mg, 360 µL, 2.60 mmol) and methanesulfonylchloride (MsCl, 148 mg, 99.9 µL, 1.30 mmol) were dissolved in in 20 mL dichloromethane and stirred for 16 hours overnight. Then, the entire mixture was poured onto a silica gel column and the product was eluted by using 0-1% methanol in dichloromethane. Yield: 316 mg of MsOc$_3$HBC-vinyl as an orange solid (93%). HR-ESI-MS (m/z): $[M+H]^+$ calcd.: 397.16, found: 397.16. ^{1}H-NMR: (400 MHz, $CDCl_3$, 25 °C): δ = 2.08 (2H, p, *J* = 6.42 Hz, $MsOCH_2C\boldsymbol{H_2}CH_2N$), 3.02 (3H, s, S-C***H₃***), 3.06 (3H, s, N-C***H₃***), 3.58 (2H, t, *J* = 6.94 Hz, $MsOCH_2CH_2C\boldsymbol{H_2}N$), 4.30 (2H, t, *J* = 5.92 Hz, $MsOC\boldsymbol{H_2}CH_2CH_2N$), 5.29 & 5.79 (2H, dxd, *J* = 30.68 Hz, CH=C***H₂*** (vinyl)), 6.73 (3H, m, C***H*** (11&13) & C***H***=CH_2 (vinyl)), 7.41 (1H, s, C***H*** (8), 7.44 (2H, d, *J* = 8.35 Hz, C***H*** (3&5)), 7.59 (2H, d, *J* = 8.45 Hz, C***H*** (2&6)), 7.85 (2H, d, *J* = 8.88 Hz, C***H*** (10&14)). ^{13}C-NMR: (100 MHz, $CDCl_3$, 25 °C): δ = 27.05 ($MsOCH_2\boldsymbol{C}H_2CH_2N$), 37.59 (S-$\boldsymbol{C}H_3$), 38.76 (N-$\boldsymbol{C}H_3$), 48.54 ($MsOCH_2CH_2\boldsymbol{C}H_2N$), 67.45 ($MsO\boldsymbol{C}H_2CH_2CH_2N$), 104.92 ***C***(7), 111.74 ***C***(11&13), 114.64 (CH=$\boldsymbol{C}H_2$ (vinyl)), 119.38 (***C***N), 122.14

C(9), 125.68 ***C***(2&6), 126.84 ***C***(3&5), 131.59 ***C***(10&14), 134.88 ***C***(4), 136.18 (***C***H=CH_2 (vinyl)), 137.52 ***C***(1), 141.93 ***C***(8), 150.42 ***C***(12). For NMR spectra see Supplementary Figures 8,9.

Synthesis of Brc$_3$DBF

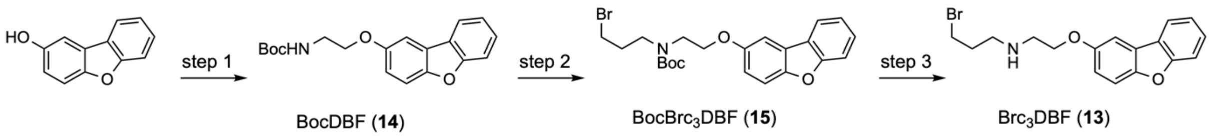

Step 1: 2-(*N-tert*-Butyloxycarbonyl)aminoethyloxy)dibenzo[*b,d*]furan (BocDFB): To a solution of 2-hydroxydibenzo[*b,d*]furan (600 mg, 3.26 mmol) and triphenylphosphine (1.28 g, 4.96 mmol, 1.5 eq) in tetrahydrofuran (25 mL) was added *tert*-butyl(2-hydroxyethyl)carbamate (756 µL, 4.89 mmol, 1.5 eq). Diethyl azodicarboxylate (960 µL, 2.44 mmol, 1.5 eq 40% solution in toluene) was introduced over the course of 10 minutes. After 3 h at room temperature, two drops of methanol were added and the reaction mixture was evaporated to dryness. The crude mixture was purified by column chromatography on silica gel (5-10% ethyl acetate in cyclohexane). Yield: 1.07 g of BocDFB as a white crystalline solid (91%). TLC: 25% ethyl acetate in cyclohexane, R_f : 0.42. ESI-MS (m/z): $[M+Na]^+$ found: 350.1358; $[M+H]^+$ calculated: 350.1363. ^{1}H-NMR (400 MHz, DMSO-d_6, 25 °C): δ 8.12 (m, 1H, ***H***C(9)), 7.74 (d, J_{HH} = 2.5 Hz, 1H, ***H***C(1)), 7.68-7.56 (m, 2H, ***H***C(4), ***H***C(6)) 7.50 (m, 1H, ***H***C(7)), 7.38 (m, 1H, ***H***C(8)), 7.13-7.01 (m, 2H, ***H***C(3) & ***H***N)), 4.06 (t, J_{HH} = 5.9 Hz, 2H, ***H₂***C(1')), 3.35 (q, J_{HH} = 5.8 Hz, 2H, $\boldsymbol{H_2}$C(2')), 1.39 (s, 9H, $\boldsymbol{H_3}$C(Boc)). ^{13}C-NMR (400 MHz, $CDCl_3$): δ 157.0 ***C***(6a), 156.1 ***C***(carbonyl, Boc), 154.9 ***C***(2), 151.2 ***C***(4a), 127.3 ***C***(7), 124.9 & 124.5 ***C***(1a) & ***C***(9a), ***C***(8)122.6, 120.7 ***C***(9), 115.7 C(3), 112.3 ***C***(4), 111.9 ***C***(6), 104.9 ***C***(1), 79.7 ***C***(tertiary, Boc), 68.2 ***C***(1'), 40.4 ***C***(2'), 28.5 ***C***(CH_3, Boc) ppm.

Step 2: 2-(*N-(tert*-Butyloxycarbonyl)-*N*-(3''-bromopropyl))-2'-aminoethoxy)dibenzo[*b,d*]furan (BocBrc3DFB)**:** To a solution of BocDFB (115 mg, 351 µmol) in *N,N*-dimethylformamide (0.5 mL) was added sodium hydride (17 mg, 0.42 mmol, 1.2 eq, 60% dispersion in mineral oil). After stirring vigorously for 15 minutes 15-crown-5 (83 µL, 0.42 mmol, 1.2 eq) was introduced and the reaction continued for 15 minutes. 1,3-dibromopropane (107 µL, 1.05 mmol, 3 eq) was added. After 16 h, the reaction was quenched upon the addition of saturated aqueous ammonium chloride. The turbid mixture was extracted with ethyl acetate three times, the combined organic extracts were washed with brine, dried over magnesium sulfate and evaporated. The compound was purified by flash column chromatography on silica gel provided. Yield: 24 mg, as a colorless solid (15%). TLC: 25% ethyl acetate in cyclohexane, R_f : 0.64. ESI-MS (m/z): $[M+Na]^+$ found: 470.0913; $[M+Na]^+$ calculated: 470.0937. ^{1}H-NMR (400 MHz, $CDCl_3$, 25 °C): δ 7.91 (d, J_{HH} = 7.6 Hz, 1H, ***H***C(9)), 7.54 (d, J_{HH} = 8.2 Hz, 1H, ***H***C(6)), 7.50-7.39 (m, 3H, ***H***C(1), ***H***C(4), ***H***C(7)), 7.32 (m, 1H, ***H***C(8)), 7.04 (dd, J_{HH} = 2.6, 9.0 Hz, 1H, ***H***C(3)), 4.31-4.10 (m, 2H, $\boldsymbol{H_2}$C(1'), 3.73-3.60 (m, 2H, $\boldsymbol{H_2}$C(2')), 3.51 (t, J_{HH} = 6.9 Hz, 2H, $\boldsymbol{H_2}$C(1'')), 3.44 (t, J_{HH} = 6.2 Hz, 2H, $\boldsymbol{H_2}$C(3'')), 2.26-2.12 (m, 2H, $\boldsymbol{H_2}$C(2'')), 1.48 (s, 9H, $\boldsymbol{H_3}$C(Boc)) ppm. ^{13}C-NMR (400 MHz, $CDCl_3$, 25 °C): δ 157.1 ***C***(6a), 155.7 ***C***(carbonyl, Boc), 155.0 ***C***(2), 151.1 ***C***(4a), 127.3 ***C***(7), 124.9 & 124.5 ***C***(1a) & ***C***(9a), 122.6 ***C***(8), 120.7 ***C***(9), 115.6 ***C***(3), 112.3 ***C***(4), 111.9 ***C***(6), 104.8 ***C***(1), 80.2 ***C***(tertiary, Boc), 67.7 ***C***(1'), 47.8 & 47.5 ***C***(2') & ***C***(1''), 32.1 ***C***(2''), 30.9 ***C***(3''), 28.6 ***C***(CH_3, Boc) ppm.

Step 3: 2-*(N*-(3''-Bromopropyl)2'-aminoethyloxy)dibenzo[*b*,*d*]furan hydrotrifluoroacetate (Brc$_3$DFB)**:** To a solution of BocBrc$_3$DFB (24 mg, 0.35 mmol) in chloroform (400 µL) was added trifluoroacetic acid (40 µL). After 2 h at room temperature the volatiles were removed *in vacuo* and the residual oil was coevaporated three times with chloroform. Yield: 24 mg of Brc$_3$DFB as a white crystalline solid (95%). ESI-MS (m/z): $[M+H]^+$ found: 348.0576; $[M+H]^+$ calculated: 348.0594. ^{1}H-NMR (400 MHz, DMSO-d_6*, 25 °C*): δ 8.86 (bs, 2H, $\boldsymbol{H_2}N^+$), 8.14 (d, J_{HH} = 7.4 Hz, 1H, ***H***C(9)), 7.79 (d, J_{HH} = 2.6 Hz, 1H, ***H***C(1)), 7.70-7.64 (m, 2H, ***H***C(4) & ***H***C(6)), 7.53 (m, 1H, ***H***C(7)), 7.40 (m, 1H, ***H***C(8)), 7.18 (dd, J_{HH} = 2.6 & 8.9 Hz, 1H, ***H***C(3)), 4.35 (t, J_{HH} = 5.0 Hz, 2H, $\boldsymbol{H_2}$C(1')), 3.64 (t, J_{HH} = 6.4 Hz, 2H, $\boldsymbol{H_2}$C(3'')), 3.50-3.43 (m, 2H, $\boldsymbol{H_2}$C(2')), 3.22-3.12 (m, 2H, $\boldsymbol{H_2}$C(1'')), 2.26-2.17 (m, 2H, $\boldsymbol{H_2}$C(2'')) ppm. ^{13}C-NMR (400 MHz, $CDCl_3$, 25 °C): δ 157.99 (CF_3***C***OO^-, q, J_{HH} = 34.1 Hz), 156.2 ***C***(6a), 154.1 C(2), 150.4 ***C***(4a), 127.7 ***C***(7), 124.2 &

123.7 ***C***(1a), & ***C***(9a), 122.9 ***C***(8), 121.2 ***C***(9), 116.1 ***C***(3), 112.4 ***C***(4), 111.8 ***C***(6), 105.5 ***C***(1), 64.4 ***C***(1’), 46.2 & 46.0 ***C***(2’) & ***C***(1’’), 31.1 ***C***(3’’), 28.6 ***C***(2’’) ppm. For NMR spectra see Supplementary Figures 10-12.

Synthesis of Br-C4-EG-Biotin

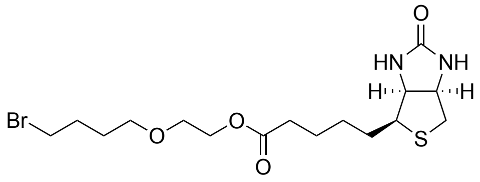

Br-C4-EG-Biotin (**16**)

To a suspension of biotin (90 mg, 369 µmol) in 1.8 mL dry DMF was added 2-(4-bromobutoxy)ethan-1-ol [3] (93 mg, 470 µmol) dissolved in 0.5 mL dry DMF. Then, *N*,*N*’-dicyclohexylcarbodiimide (84 mg, 405 µmol) and *N*,*N*-dimethylaminopyridine (DMAP, 2.5 mg) were added and stirred at room temperature overnight. Afterwards, the slightly cloudy solution was heated to 60 °C for 30 minutes, until a clear yellowish solution remained. All volatiles were removed under vacuo and the crude compound was purified using silica gel chromatography with 0 to 10% methanol in dichloromethane as gradient. Yield: 62 mg of Br-C4-EG-Biotin as a colorless solid (40%). TLC: (10% methanol in dichloromethane): R_f = 0.94 spots were visualized with dimethylaminocinnamaldehyde staining (pink spots). HR-ESI-MS (m/z): $[M+H]^+$ calcd.: 423.09 & 425.09, found: 423.09 & 425.09. ^{1}H-NMR (400 MHz, DMSO-d_6, 25 °C): δ 5.82 (s, 1H, ***H***N(1‘‘‘)), 5.28 (s, 1H, ***H***N(3‘‘‘)), 4.52-4.47 (m, 1H, ***H***C(6‘‘‘a)), 4.33-4.28 (m, 1H, ***H***C(3’’’a), 4.23-4.18 (m, 2H, $\boldsymbol{H_2}$C(1’)), 3.61 (t, J_{HH} = 4.7 Hz, 2H, $\boldsymbol{H_2}$C(2’)), 3.50 (t, J_{HH} = 6.3 Hz, 2H, $\boldsymbol{H_2}$C(4’’)), 3.44 (t, J_{HH} = 6.8, 2H, $\boldsymbol{H_2}$C(4’’)), 3.18-3.11 (m, 1H, ***H***C(4’’’)), 2.90 (dd, J_{HH} = 5.0, 12.8 Hz, 1H, $\boldsymbol{H_a}$C(6’’’)), 2.73 (d, J_{HH} = 12.8 Hz, 1H, $\boldsymbol{H_b}$C(6'''')), 2.37 (t, J_{HH} = 7.6, 2H, $\boldsymbol{H_2}$C(2’)), 1.99-1.90 (m, 2H, $\boldsymbol{H_2}$C(3’’)), 1.78-1.62 (m, 6H, $\boldsymbol{H_2}$C(3) & $\boldsymbol{H_2}$C(4) & $\boldsymbol{H_2}$C(2’’)), 1.51-1.39 (m, 2H, $\boldsymbol{H_2}$C(5)) ppm. ^{13}C-NMR (100 MHz, DMSO-d_6, 25 °C): δ 173.8 ***C***(1), 163.6 ***C***(2’’’), 70.4 ***C***(1’’), 68.8 ***C***(2’), 63.5 ***C***(1’), 62.1 ***C***(3’’’a), 60.2 ***C***(6’’’a), 55.6 ***C***(4’’’), 40.7 ***C***(6’’’), 33.9 & 33.8 ***C***(4’’) & ***C***(2), 29.7 ***C***(3’’), 28.4 & 28.4 & 28.3 ***C***(3) & ***C***(5), ***C***(2’’) ppm. For NMR spectra see Supplementary Figures 13,14.

References and Notes

[1] Neuner, E., Frener, M., Lusser, A. & Micura, R. Superior cellular activities of azido- over amino-functionalized ligands for engineered preQ1 riboswitches in *E.coli*. *RNA Biol.* **15**, 1376–1383 (2018).

[2] Chen, X. *et al.* Visualizing RNA dynamics in live cells with bright and stable fluorescent RNAs. *Nat. Biotechnol.* **37**, 1287–1293 (2019).

[3] 2-(4-Bromobutoxy)ethan-1-ol was obtained by 4,4’-dimethoxytritylation of ethylene glycol, followed by alkylation using 1,4-dibromobutane, and subsequent detritylation.

Supplementary Tables

Supplementary Table 1. Comparison of preQ$_1$ and Pepper systems for covalent RNA labeling to literature data of other ribozymes.

Ribozyme	Characterization data (selection)				Michaelis-Menten			
	**Size**	**pH**	**Mg2+**	**k(obs)**	**k(cat)**	**Km**	**k(cat)/Km**	*)
	nt		[mM]	[min-1]	[min-1]	[µM]	[M-1 min-1]	
**This work - Self-alkylating *Tt* preQ1-I riboswitch aptamer** (Bromide)			in a typical experiment					
preQ1 aptamer wt C15 + Brc3DPQ1	33	6.0	2.0	0.00382	0.00337	0.162	**20782**	
preQ1 aptamer C15U + Brc3DPQ1	33	6.0	2.0	0.00270	0.00335	0.320	**10469**	
preQ1 aptamer wt C15 + Brc3preQ1	33	6.0	2.0	0.00225	0.00253	0.173	**14644**	
preQ1 aptamer C15U + Brc3preQ1	33	6.0	2.0	0.00076	n.d.	n.d.	n.d.	
**This work - Self-alkylating *co* FLAP** (Mesylate)								
Pepper aptamer + MsOc3HBC	45	6.0	2.0	0.00621	–	–	**1773810**	**)
***In vitro* selected self-biotinyating ribozyme** (Methylepoxide) – David Liu & coworkers								
McDonald, R. I. et al. Electrophilic activity-based RNA probes reveal a self-alkylating RNA for RNA labeling. *Nat. Chem. Biol*. 10, 1049–1054 (2014))								
"Liu" ribozyme	42	7.4	10.0		0.00160	12000	**0.1**	
***In vitro* selected self-biotinyating ribozyme "Liu"** (Epoxide) – J. Piccirilli & coworkers								
Krochmal, D. et al. Structural basis for substrate binding and catalysis by a self-alkylating ribozyme. *Nat. Chem. Biol*. 18, 376–384 (2022).								
"Liu" ribozyme + Methylepoxide-Biotin	42	7.4	10.0	0.00260	0.00410	2100	**2.0**	
"Liu" ribozyme + Epoxide-Biotin	58	7.4	10.0	0.00640	–	–	**3.0**	***)
**This work** "Liu" ribozyme + Epoxide-Biotin	58	7.4	5.0	0.00675	–	–	**3.2**	***)
**This work – "Liu" ribozyme** (Bromide)								
"Liu" ribozyme + Bromoalkyl-Biotin	58	7.4	5.0	0.12395	–	–	**59.0**	***)
***In vitro* selected self-alkylating ribozyme** (Chloroacetamide) – A. Jäschke & coworkers								
Ameta, S. & Jäschke, A. An RNA catalyst that reacts with a mechanistic inhibitor of serine proteases. *Chem. Sci.* 4, 957–964 (2012).								
Ribozyme + Biotin-PEG4-D-Phe-Pro-Arg-chloromethyl ketone	232	5.0	5.0	n.d.	0.02900	1450	**20.0**	
***In vitro* selected self-alkylating ribozyme** (Iodoacetamide) – J. Heemstra & coworkers								
Sharma, A. K. et al. Fluorescent RNA Labeling Using Self-Alkylating Ribozymes. *ACS Chem. Biol*. 9, 1680–1684 (2014).								
Ribozyme **1** + 5-(Iodacetamido)fluorescein	157	7.4	5.0	0.00760	0.00035	158	**2.2**	
Ribozyme **5FR1** + 5-(Iodacetamido)fluorescein	135	7.4	5.0	0.00730	0.00063	367	**1.7**	
***In vitro* selected self-biotinyating ribozyme** (Iodoacetamide) – J. Szostak & coworkers								
Wilson, C. & Szostak, J. W. In vitro evolution of a self-alkylatIng ribozyme. *Nature* 374, 777–782 (1995).								
Ribozyme **BL8-6** + *N*-biotinoyl-*N*-iodoacetyl-ethylenediamine (BIE)	155	7.4	5.0	0.00100	–	1000	**1.0**	
Ribozyme **BL2.8-7** + *N*-biotinoyl-*N*-iodoacetyl-ethylenediamine (BIE)	155	7.4	5.0	0.05000	0.62400	–	–	
***In vitro* selected methylating ribozyme** (O6-Methylguanine) – C. Höbartner & coworkers								
Scheitl, C. P. M. et al., Structure and mechanism of the methyltransferase ribozyme MTR1. *Nat. Chem. Biol.* 18, 547–555 (2022).								
Ribozyme **MTR1** + m6G	40	7.5	10.0	0.00788	0.00310	98	**31.6**	
Ribozyme **MTR1** + m6G	40	6.0	10.0	0.06373	0.21000	228	**921.1**	
***In vitro* selected propargylating ribozyme** (Propargyl-sulfonium) – C. Höbartner & coworkers								
Okuda, T. et al., A SAM analogue-utilizing ribozyme for site-specific RNA alkylation in living cells. *Nat. Chem*. 15, 1523–1531 (2023).								
Ribozyme **SAMURI** + *S*-Propargyl SAM-amide	29	7.5	10.0	0.12000	0.09300	33	**2853**	

*) **kcat/Km**: When kcat and Km are expressed together as kcat/Km, it represents the catalytic efficiency of the enzyme. Essentially, it indicates how efficiently an enzyme converts substrate into product at low substrate concentrations, taking into account both the enzyme's affinity for the substrate (Km) and its turnover rate (kcat).

**) estimated, using the reported Kd of 3.5 nM (Chen, X. et al. Visualizing RNA dynamics in live cells with bright and stable fluorescent RNAs. *Nat. Biotechnol*. 37, 1287–1293 (2019).

***) estimated, using the reported Kd of 2.1 µM (Krochmal, D. et al. Structural basis for substrate binding and catalysis by a self-alkylating ribozyme. *Nat. Chem. Biol*. 18, 376–384 (2022).

Supplementary Table 2. Synthetic and *in vitro* transcribed RNAs.

RNA	Sequence (5' → 3')	nt	Molecular weight	
			calc.	found
	Chemical synthesis			
Tt wt	CUGGGUCGCAGUAACCCCAGUUAACAAAACAAG	33	10582.50	10582.49
Tt C15U	CUGGGUCGCAGUAAUCCCAGUUAACAAAACAAG	33	10583.49	10583.59
Tt c^7G	CUGGc^7GUCGCAGUAACCCCAGUUAACAAAACAAG	33	10567.49	10567.78
Tt G5A, C16U	CUGGAUCGCAGUAACUCCAGUUAACAAAACAAG	33	10581.50	10581.55
B. subtilis	AGAGGUUCUAGCUACACCCUCUAUAAAAAACUAA	34	10818.62	10818.53
B. subtilis C17U	AGAGGUUCUAGCUACAUCCUCUAUAAAAAACUAA	34	10819.63	10619.54
C. antarcticus	UGUGGUUCGCAACCAUCCCACAUAAAAAAACUAG	34	10833.63	10833.43
C. antarcticus C17U	UGUGGUUCGCAACCAUUCCACAUAAAAAAACUAG	34	10834.62	10834.59
L. monocytogenes	ACGUGGUUCAUUCAUACCAUCCCACGUAAAAAAAC UAGGAG	41	13099.99	13099.55
S. pneumoniae	CUUGGUGCUUAGCUUCUUUCACCAAGCAUAUUAC ACGCGGAUAACCGCCAAAGGA	55	17537.58	17537.89
S. dysenteriae	AUUGGGUUCCCUCACCCCAAUGGUUAAUCAAAAAG GU	37	11784.14	11784.81
L. rhamnosus	ACGACGAUACUUAUUUCCUUUGAUCGUCGUUAUU ACUGGCAAAGCCACAAAGGAG	55	17563.59	17563.30
Pepper	GGCGCACUGGCGCUGCGCCUUCGGGCGCCAAUCGU AGCGUGUCGGCGCC	49	15756.47	15756.59
Pepper c^7G	GGCGCACUGGCGCUGCGCCUUCGGGCGCCAAUCGU AGCGUc^7GUCGGCGCC	49	15755.48	15754.69
58 nt (*Liu*)	GGCCGCUCCAGAAGAGGGCCCCCUUGCCCGUUAUC GGGGGCUAGGCUCGAUGUCGGCC	58	18657.22	18656.68
	In vitro transcription			
155 nt (*Szostak*)	GGAGGCACCACGGCUGGAUCCGGUUUAUUAUCAU GAGCCCGACUCGGGCAGCACUGUACAUAAGCUCGG AUGCCAUAGUUUAGACACUAUGGACGUAAAGCCCA UGCUAGGCAAAGACAUUGACUGCAUGAGCGCCGCC UUGGUCAUUAGGAUCG	155	-	-
232 nt (*Jäschke*)	GGAGCUCAGCCUUCACUGCUGGCCCCUCAUUCUCC GACAAUGUACGACCUUGCAUAUACCGCUAGCACGA ACGGUGUAGAUACCUGGAUCAUUACAACACCACGA UCUUCAAAUCGAAGAUGUUCGCAUGAUGUGCGCU AGCAAUAUAGUUUAGCGAGUAUAGCCGAACGCCG UGUUGAGUACCUAACGAUACCGGUGUGAGGUGCC UGUCUGGCACCACGGUCGGAUCCAC	232	-	-
	Commercial source: primers and DNA fragments			
Pepper_fwd	GGCCGCCGGCGCACTGGCGCTGCGCCTTCGGGCGCC AATCGTAGCGTGTCGGCGCCGTGGCCGC			
Pepper rev	GGCCACGGCGCCGACACGCTACGATTGGCGCCCGAA GGCGCAGCGCCAGTGCGCCGGC			

Extension primer preQ1	5Alexa647N/GTATCGGACCGATTACCTC			
F30-preQ1-Pepper (dsDNA)	CCGAGTGCGGCCGCTTGCCATGTGTATCGGTCCGTTC ACTGGGTCGCAGTAACCCCAGTTAACAAAACAAGGG AGGTAATCGGTCCGATACTCTGATGATGGGTCCCAAA AGGCGCACTGGCGCTGCGCCTTCGGGCGCCAATCGT AGCGTGTCGGCGCCAAAAAAGGGTCCCATCATTCATG GCAAGTGGCCGCGGTCGGC 3'			

Supplementary Figures

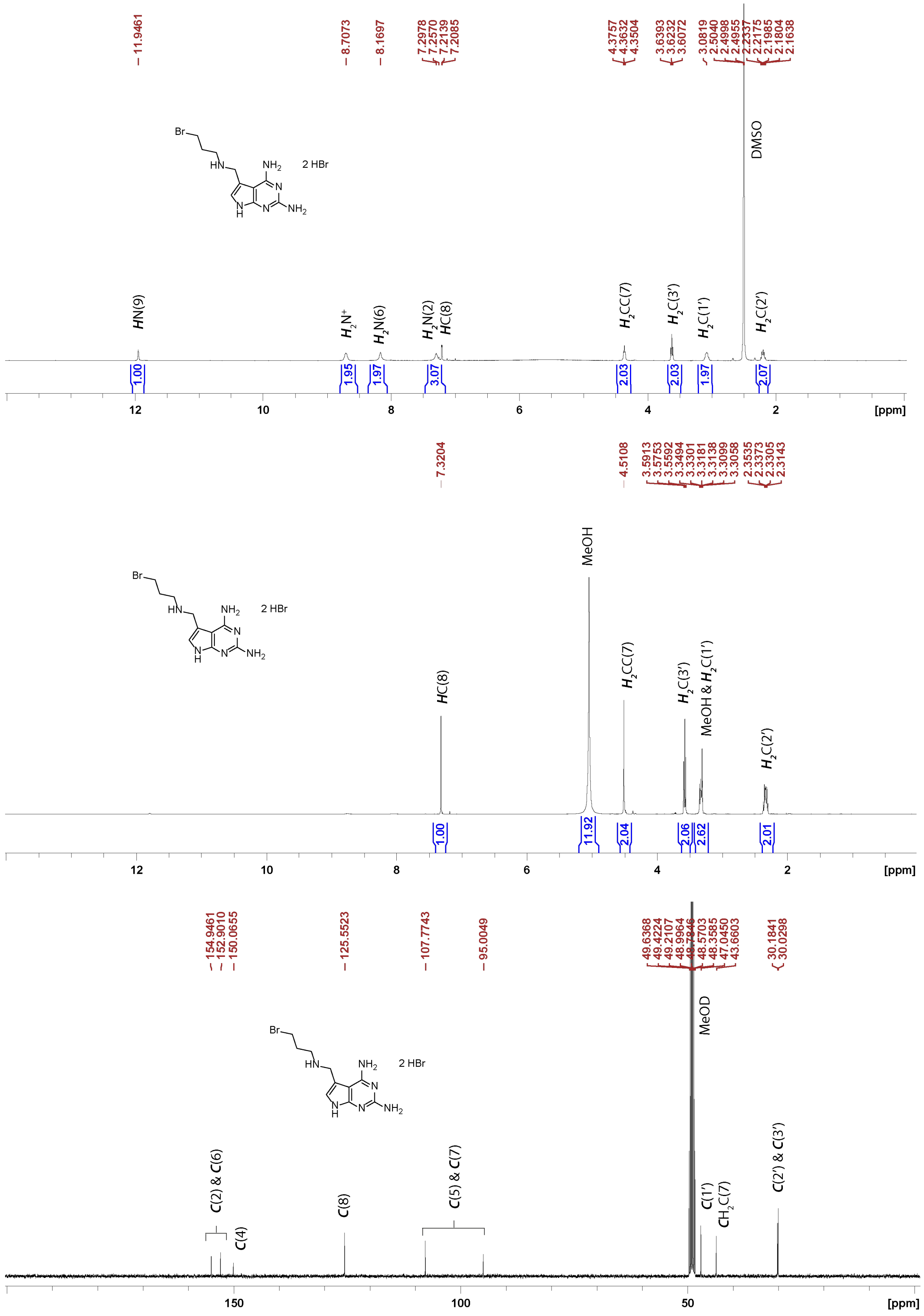

Supplementary Fig. 1 | NMR spectroscopic analysis of Brc$_3$DPQ$_1$ ligand. **a**) ^{1}H NMR spectrum (400 MHz, DMSO-*d$_6$*), top; ^{1}H NMR (400 MHz, CD$_3$OD) spectrum, middle; ^{13}C NMR (400 MHz, CD$_3$OD) spectrum (bottom).

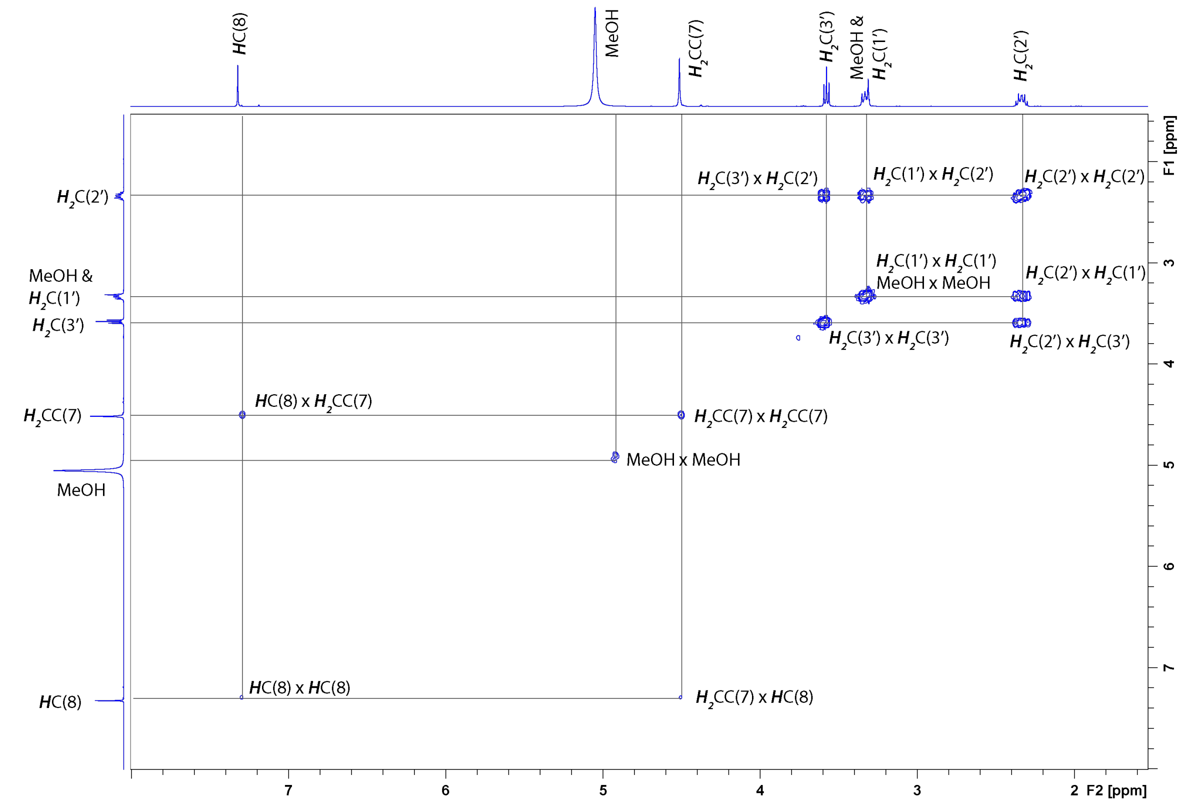

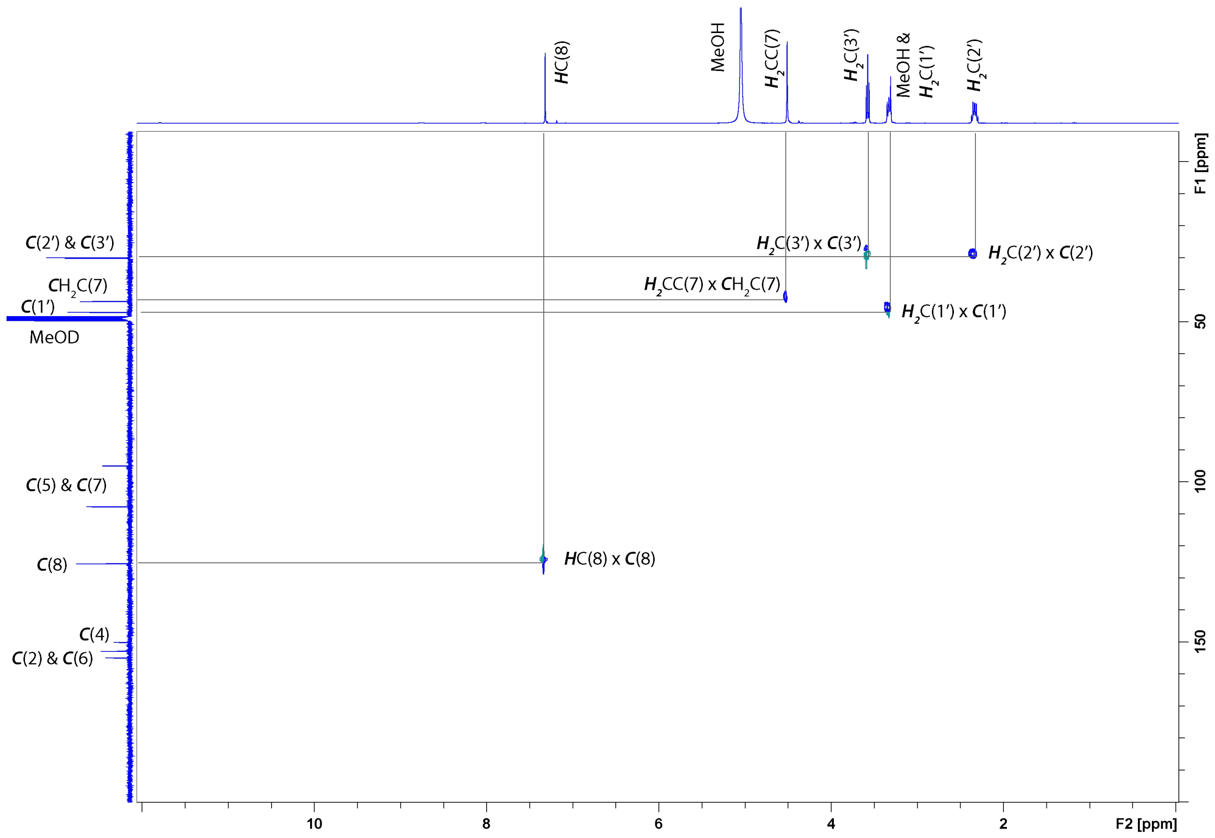

Supplementary Fig. 2 | NMR spectroscopic analysis of Brc$_3$DPQ$_1$ ligand. ^{1}H-^{1}H COSY (400 MHz, CD$_3$OD) spectrum, top; ^{1}H-^{13}C HSQC NMR (400 MHz, CD$_3$OD) spectrum, middle; ^{1}H-^{13}C HMBC (400 MHz, CD$_3$OD) NMR spectrum, bottom.

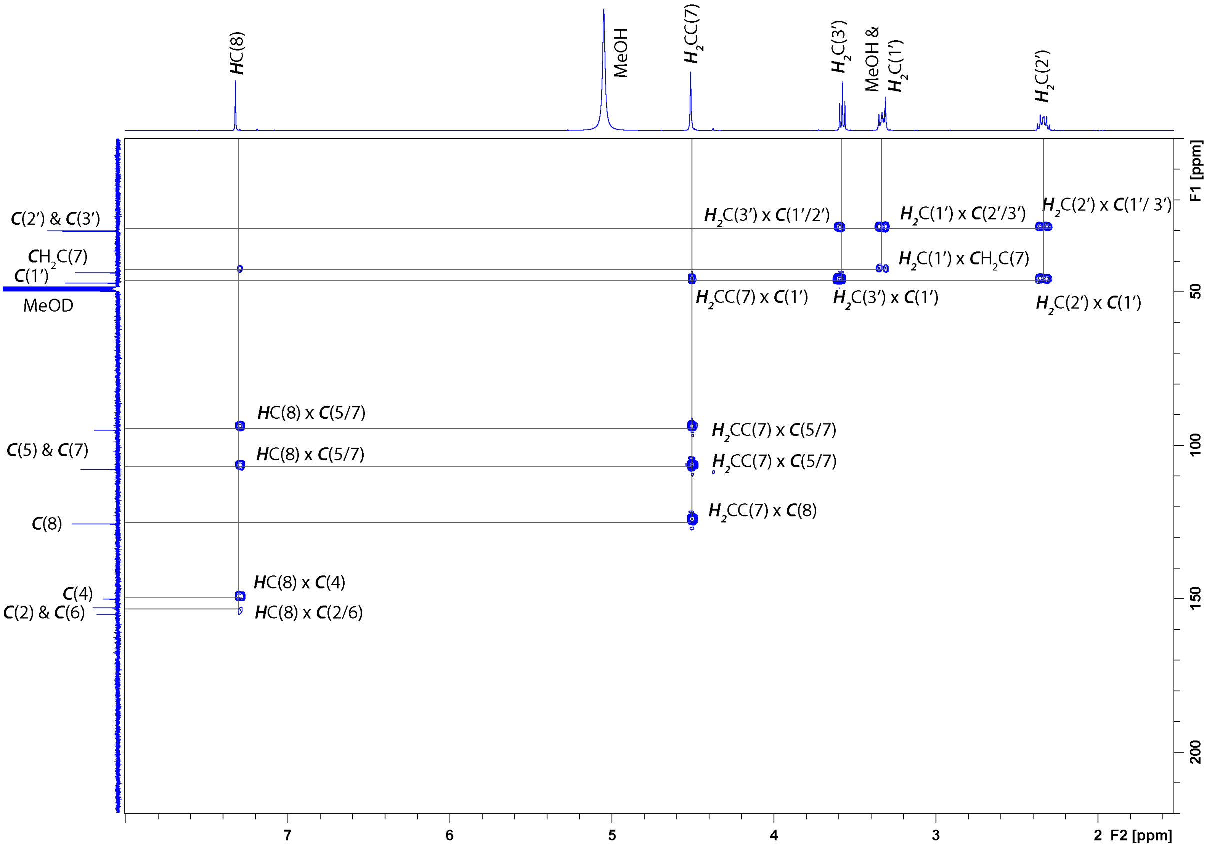

Supplementary Fig. 3 | NMR spectroscopic analysis of Brc$_3$DPQ$_1$ ligand. ^{1}H-^{13}C HMBC (400 MHz, CD$_3$OD) NMR spectrum.

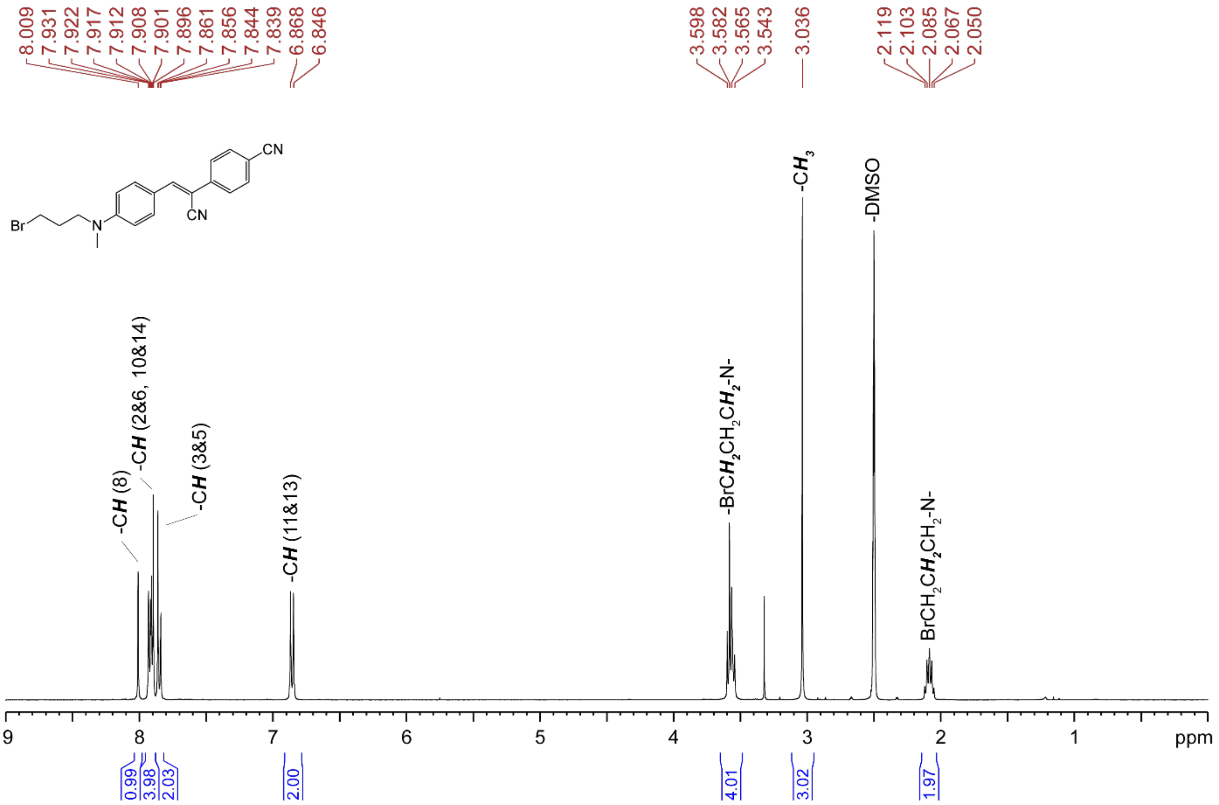

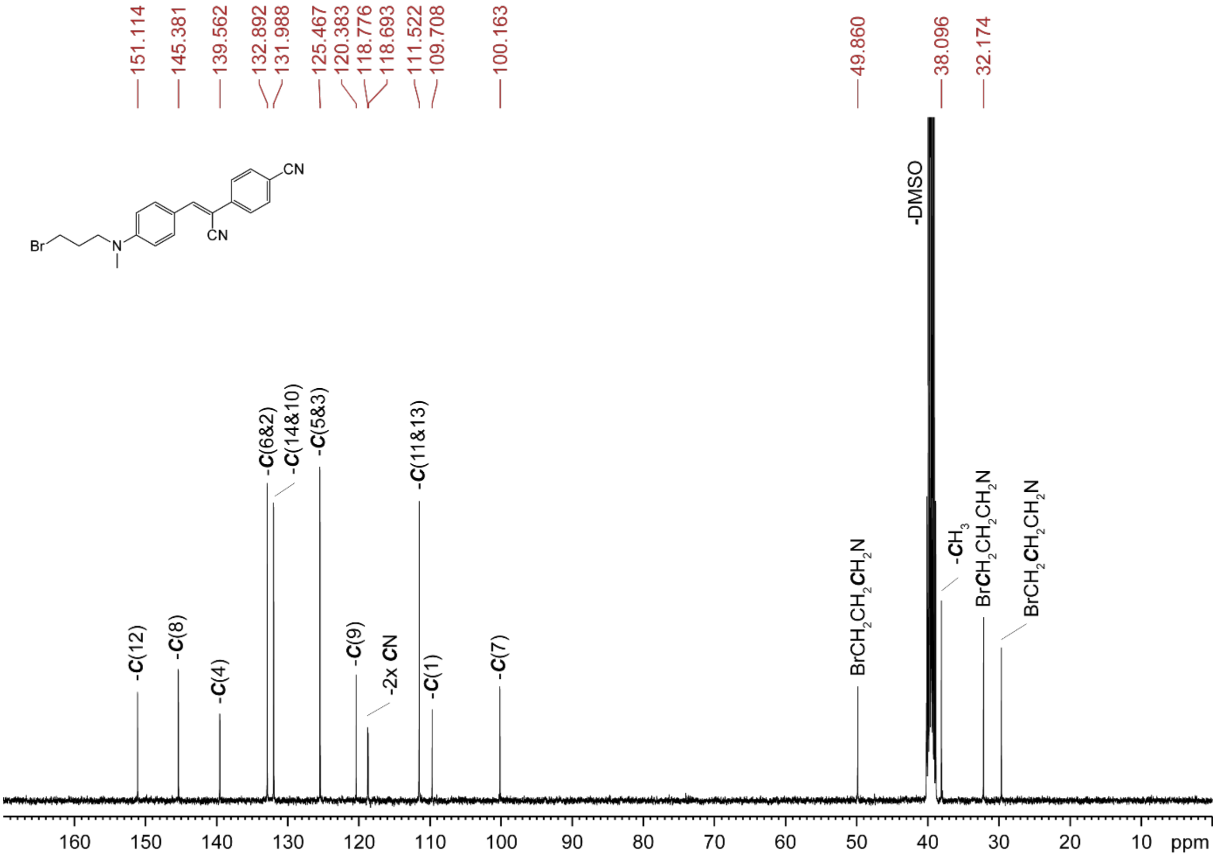

Supplementary Fig. 4 | NMR spectroscopic analysis of the Brc3HBC ligand. ^{1}H NMR (400 MHz) spectrum, top; ^{13}C NMR spectrum (100 MHz), bottom.

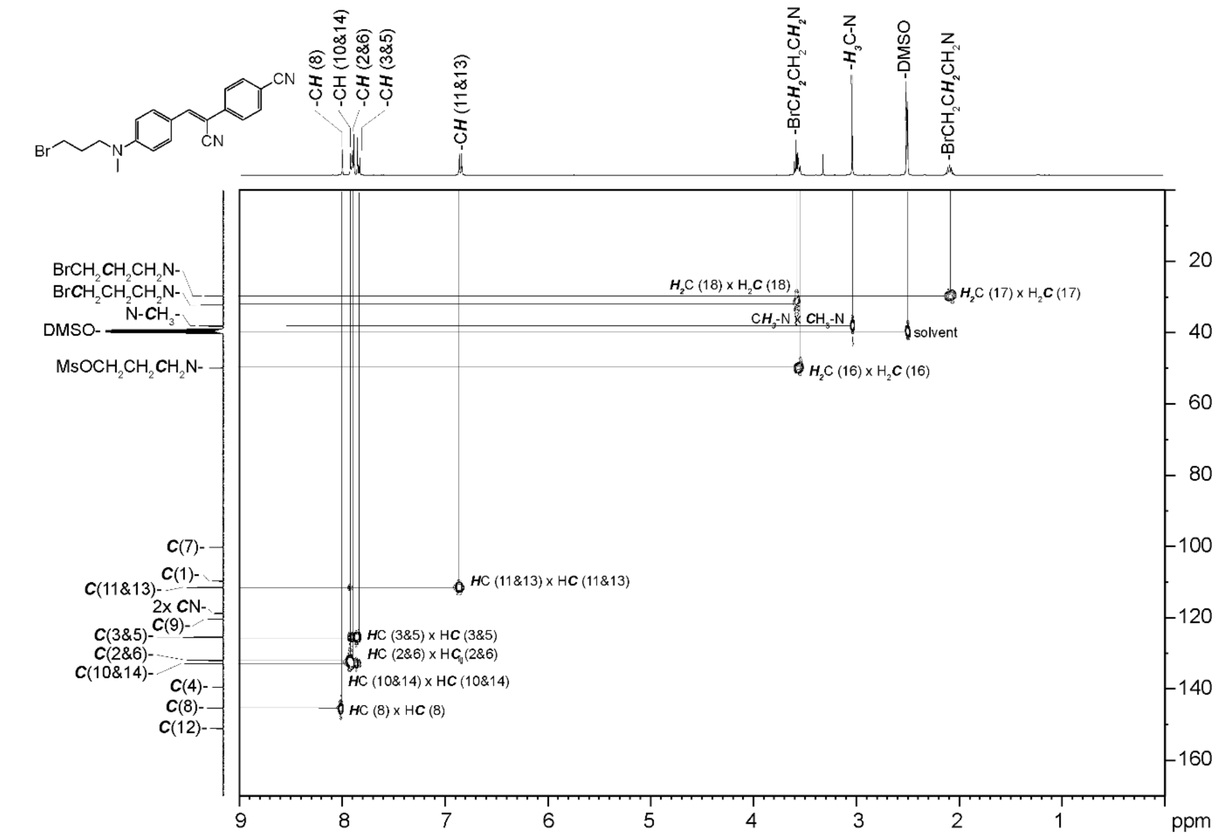

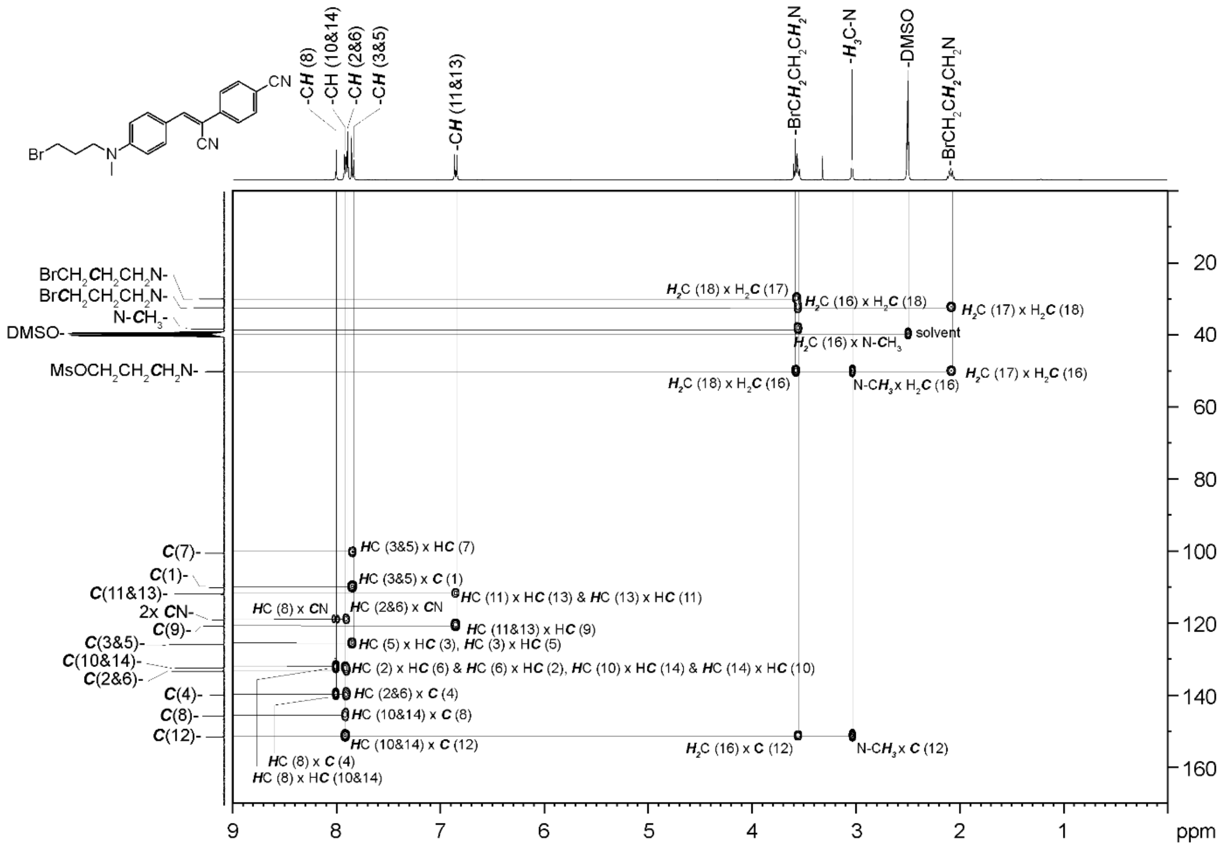

Supplementary Fig. 5 | NMR spectroscopic analysis of the Brc3HBC ligand. ^{1}H-^{13}C HSQC NMR spectrum, top; ^{1}H-^{13}C HMBC NMR spectrum, bottom.

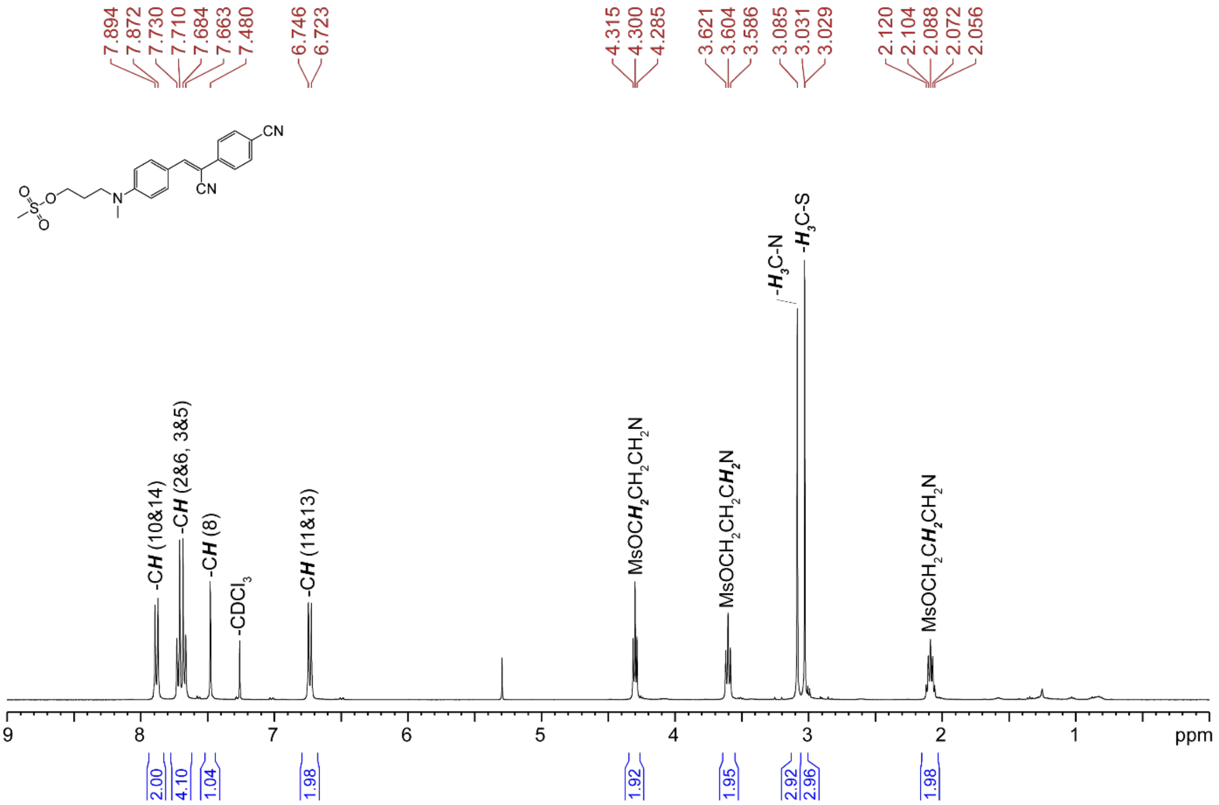

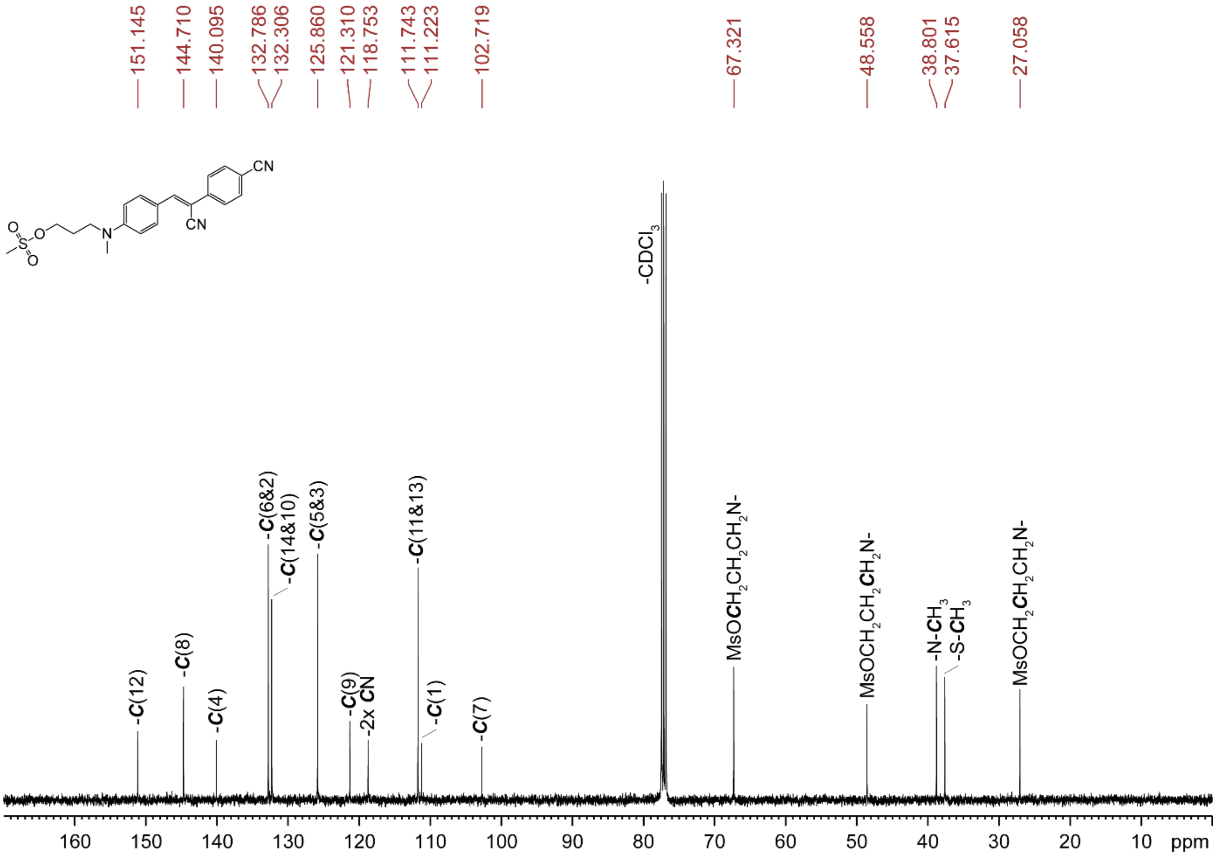

Supplementary Fig. 6 | NMR spectroscopic analysis of MsOc3HBC ligand. ^{1}H NMR (400 MHz) spectrum, top; ^{13}C NMR spectrum (100 MHz), bottom.

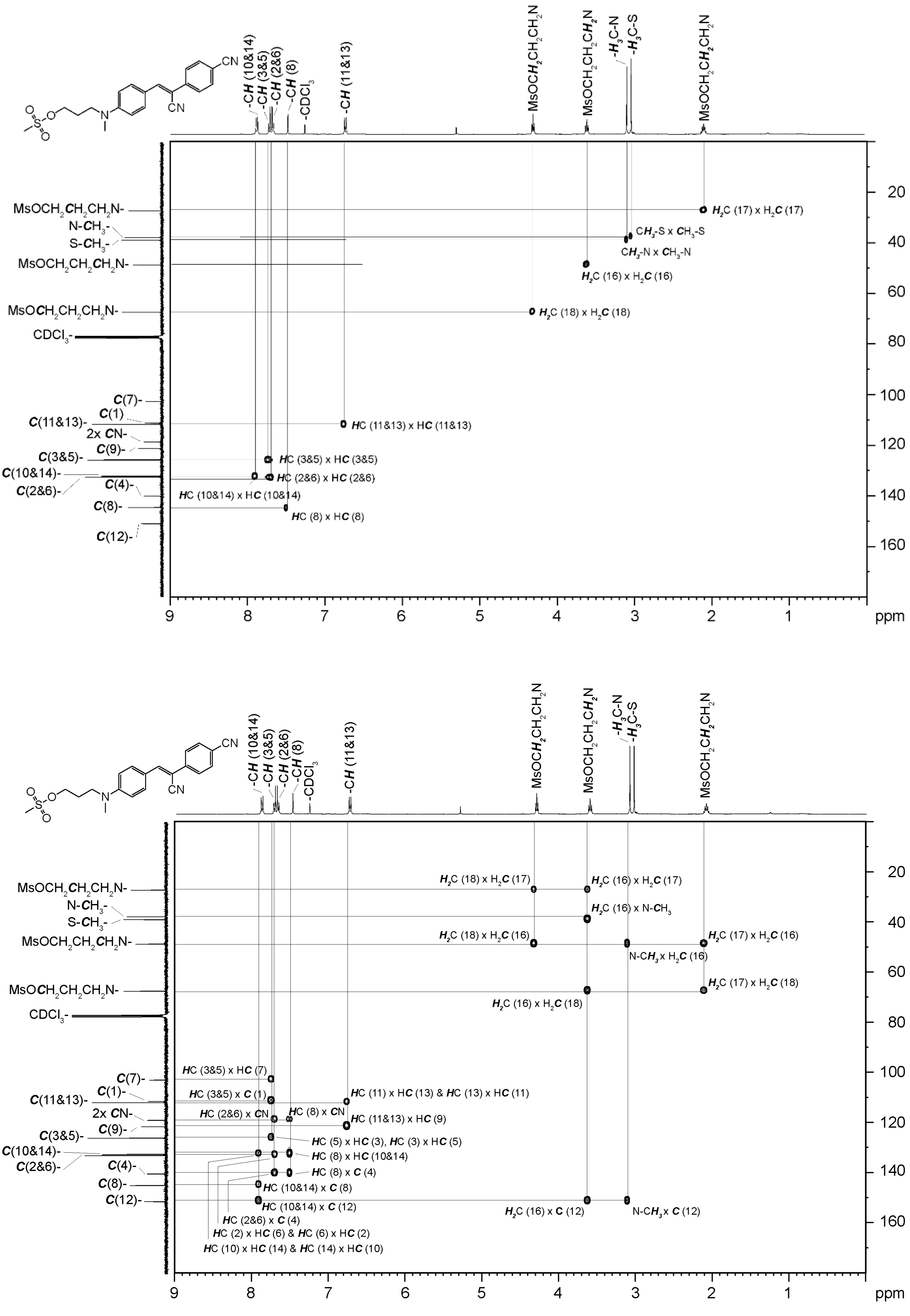

Supplementary Fig. 7 | NMR spectroscopic analysis of MsOc3HBC ligand. ^{1}H-^{13}C HSQC NMR spectrum, top; ^{1}H-^{13}C HMBC NMR spectrum, bottom.

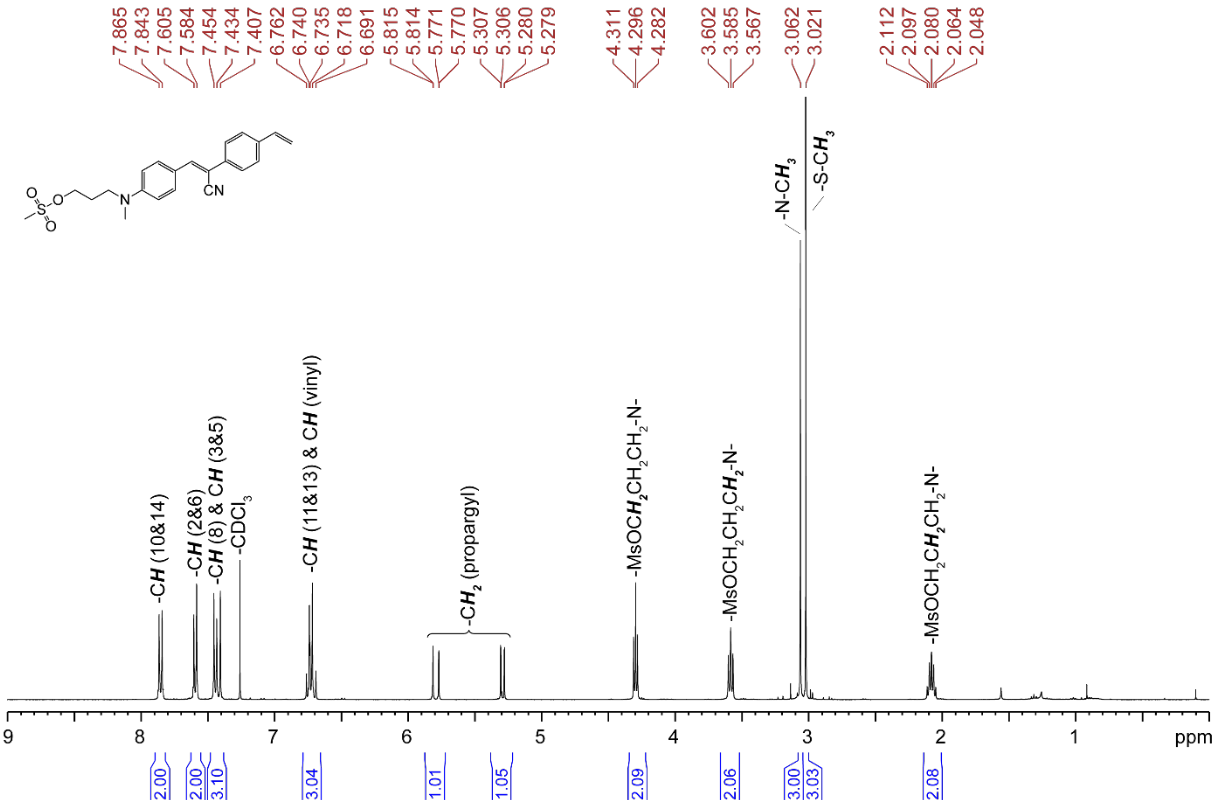

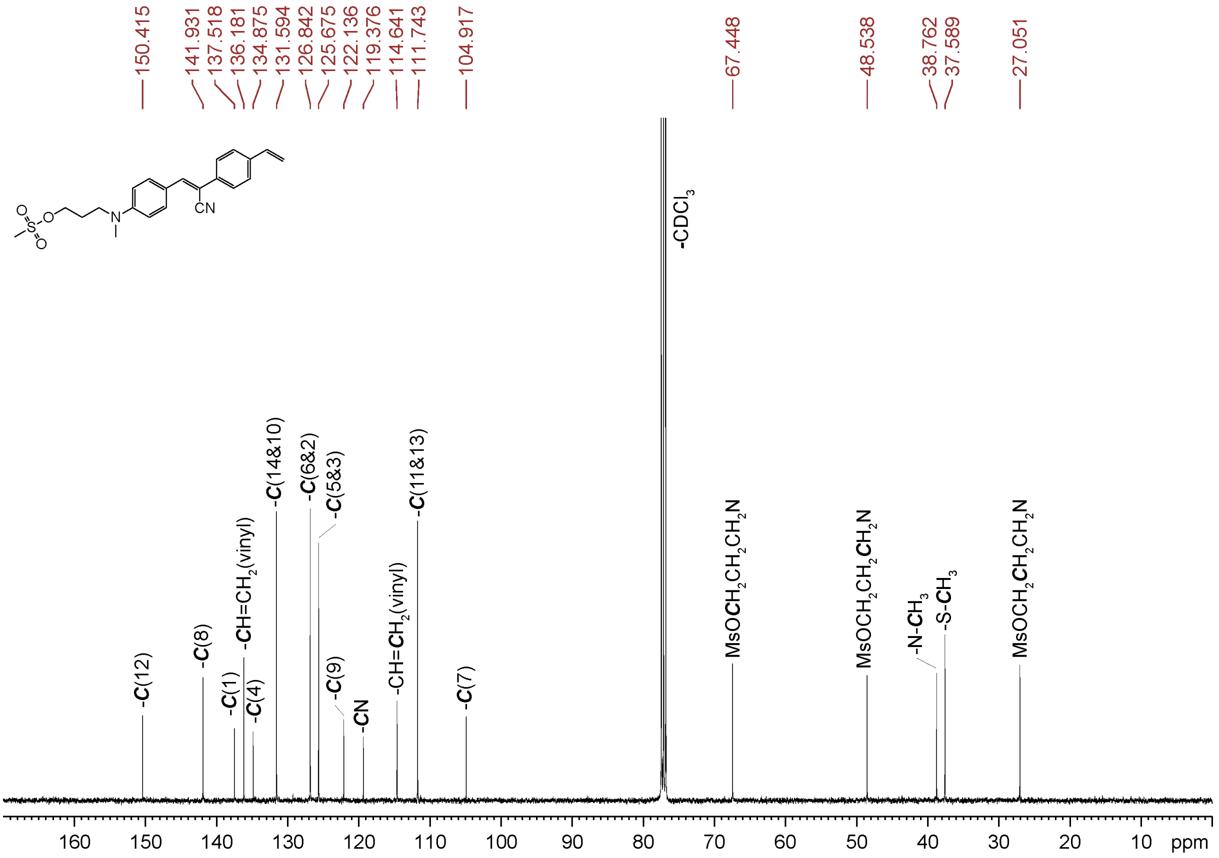

Supplementary Fig. 8 | NMR spectroscopic analysis of MsOc3HBC-vinyl ligand. ^{1}H NMR spectrum (400 MHz), top; ^{13}C NMR spectrum (100 MHz), bottom.

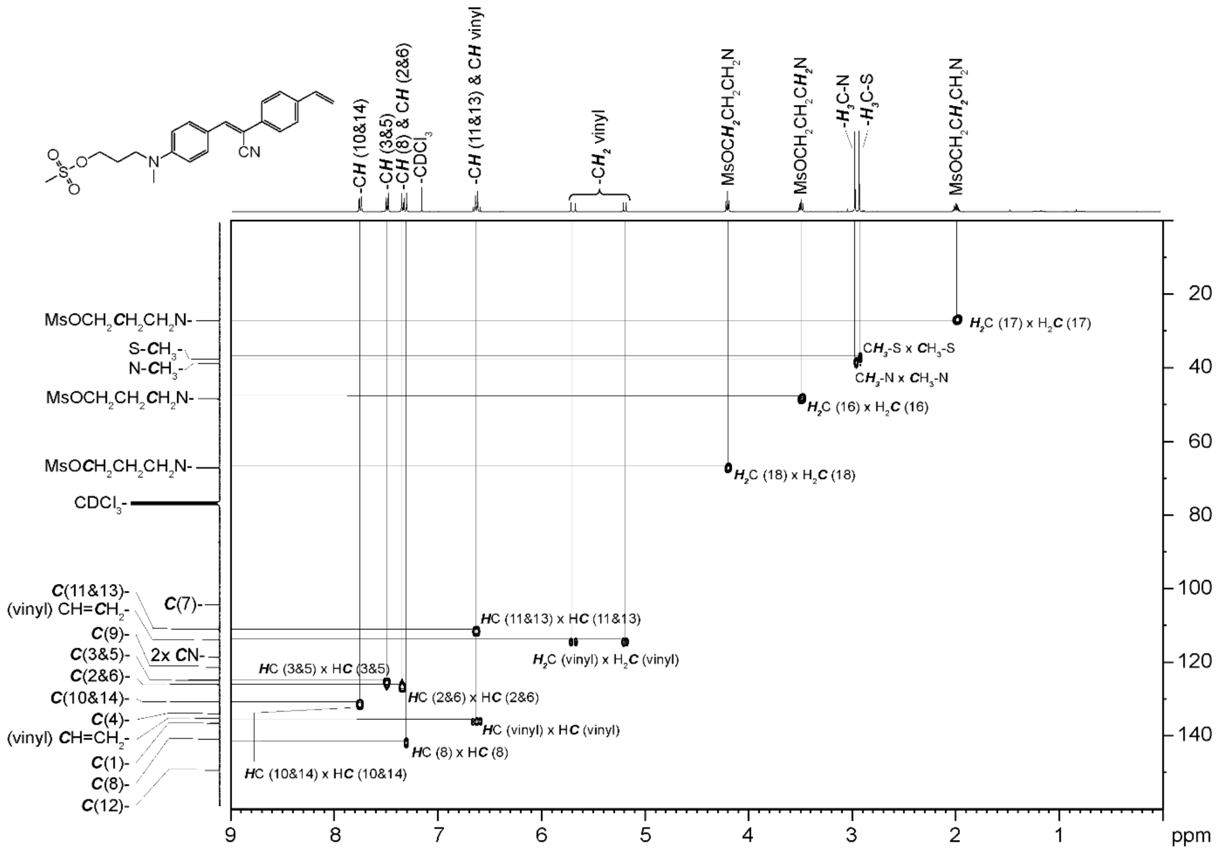

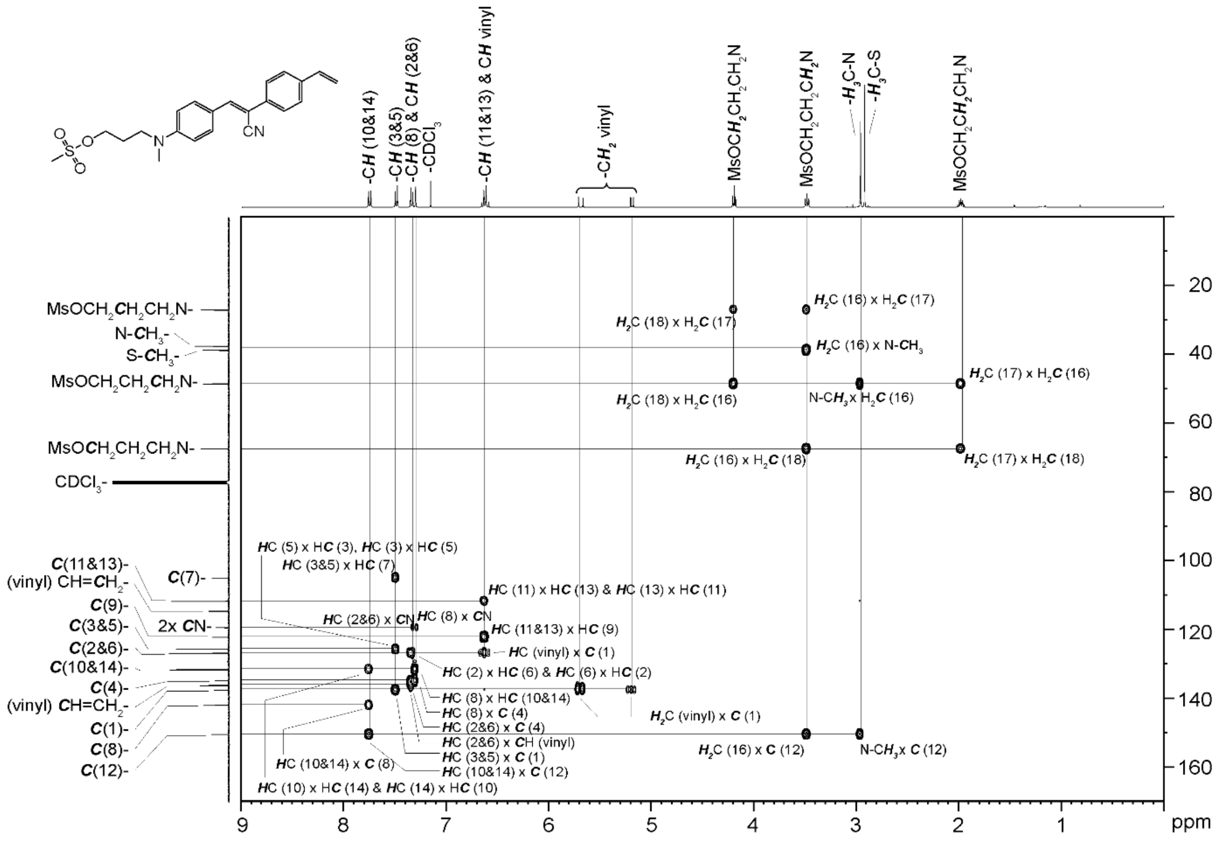

Supplementary Fig. 9 | NMR spectroscopic analysis of MsOc3HBC-vinyl ligand. ^{1}H-^{13}C HSQC NMR spectrum, top; ^{1}H-^{13}C HMBC NMR spectrum, bottom.

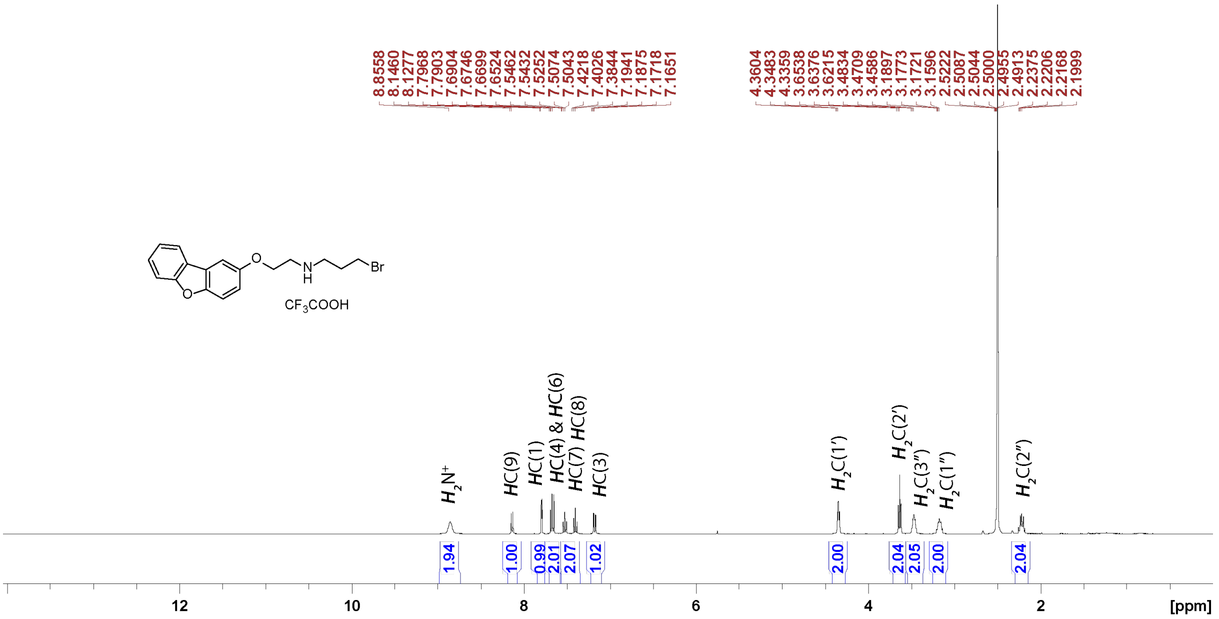

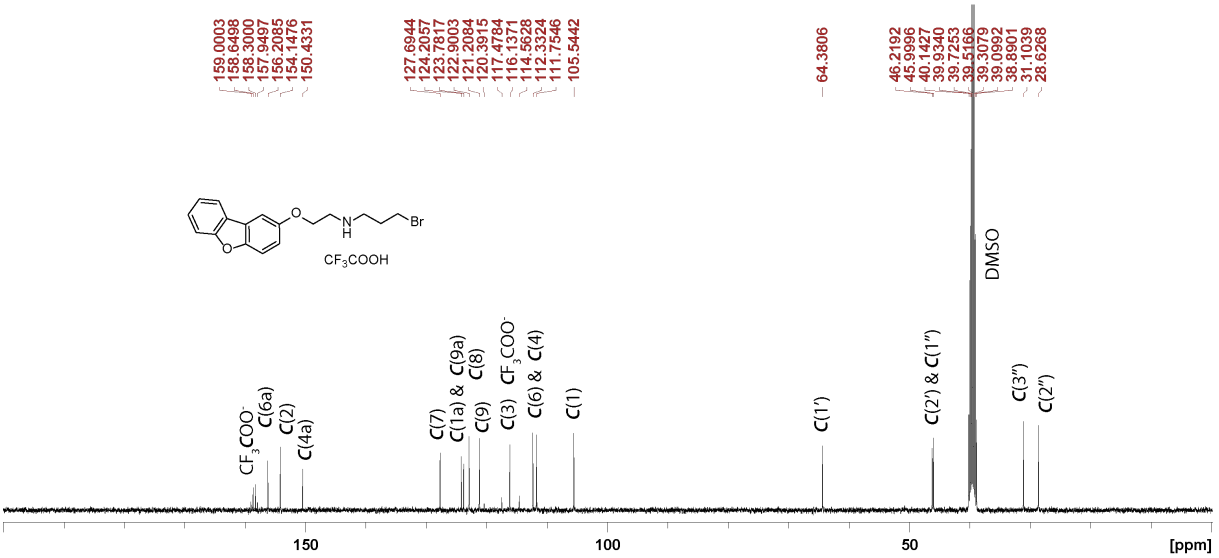

Supplementary Fig. 10 | NMR spectroscopic analysis of Brc3DBF. ^{1}H NMR spectrum (400 MHz, DMSO-d_6), top; ^{13}C NMR (400 MHz, CD_3OD) spectrum, bottom.

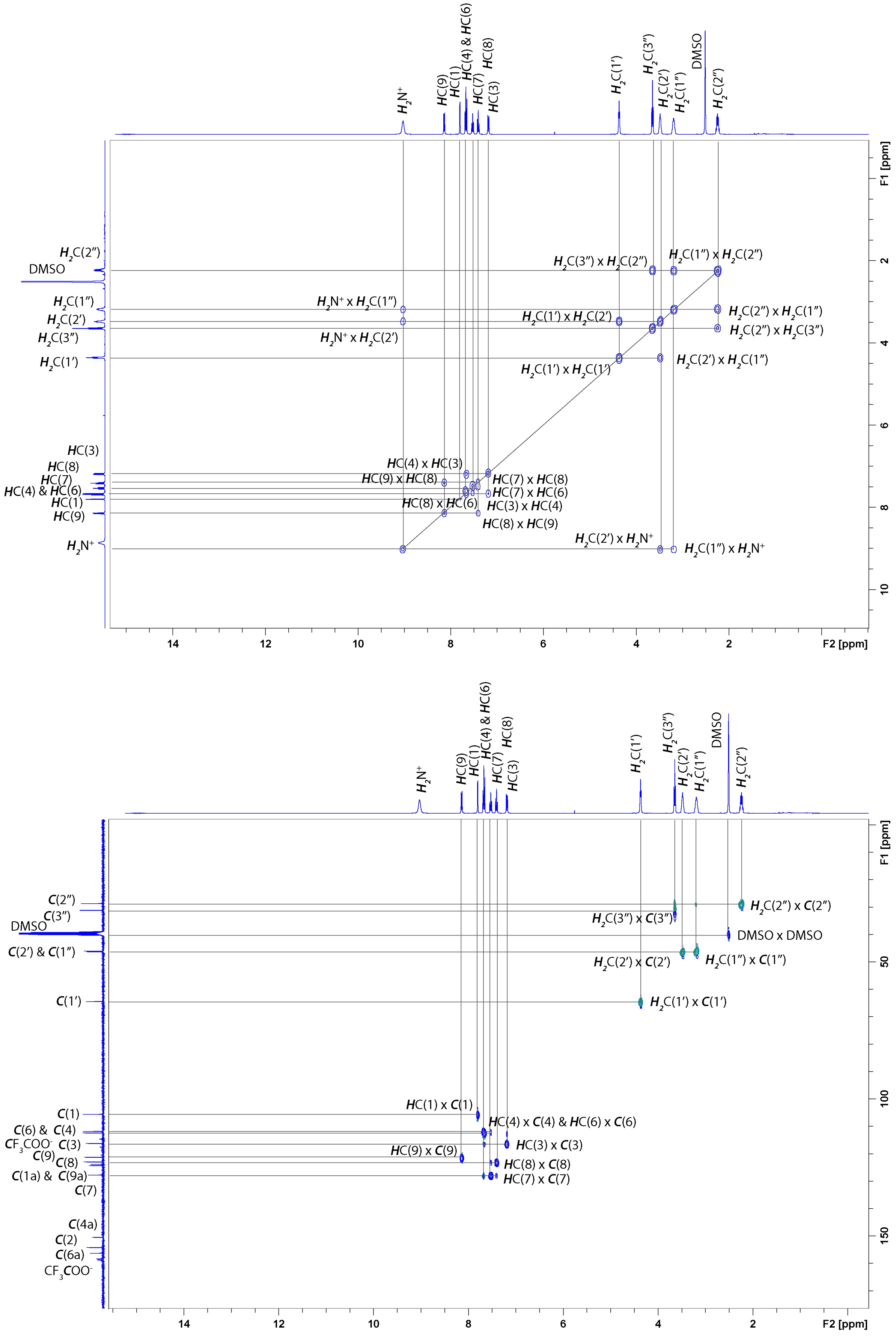

Supplementary Fig. 11 | NMR spectroscopic analysis of Brc3DBF. ^{1}H-^{1}H COSY (400 MHz, DMSO-d_6) spectrum, top; ^{1}H-^{13}C HSQC NMR (400 MHz, DMSO-d_6) spectrum, bottom.

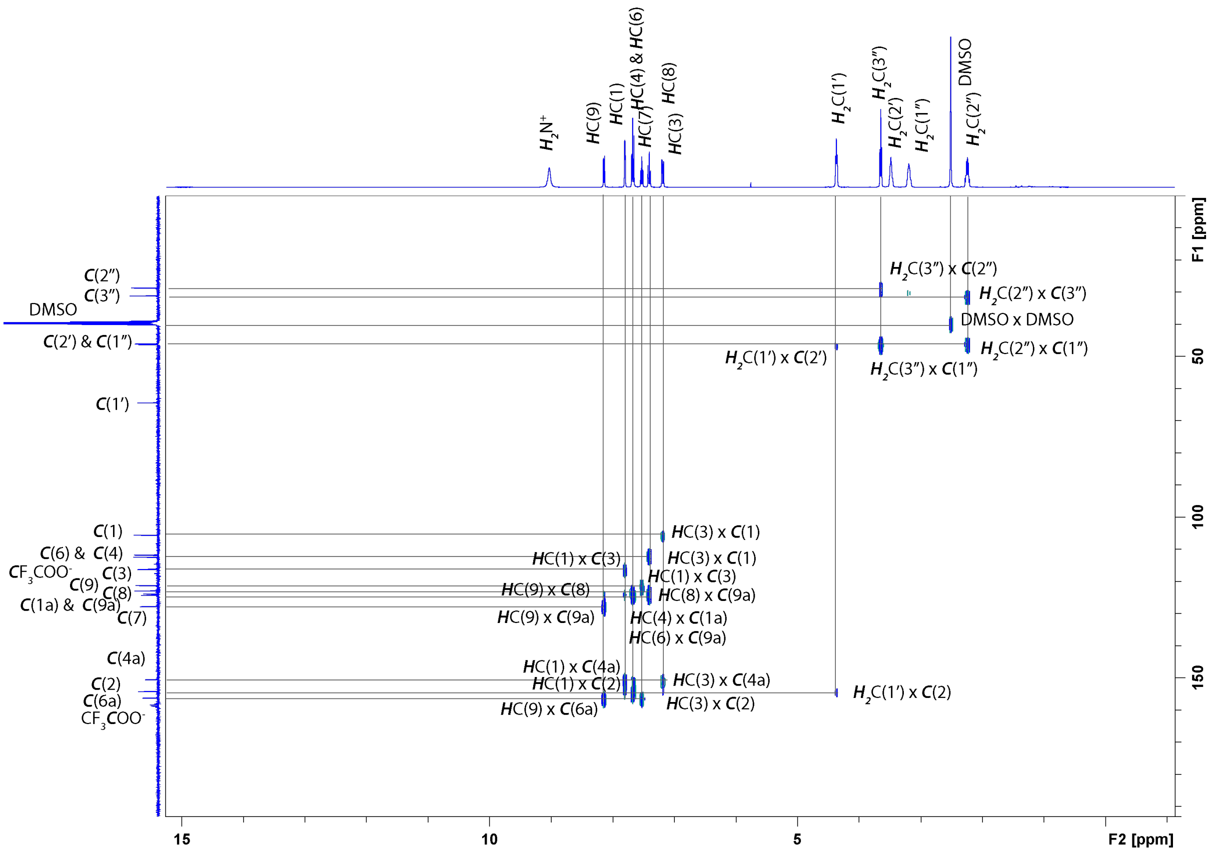

Supplementary Fig. 12 | NMR spectroscopic analysis of Brc3DBF. ^{1}H-^{13}C HMBC (400 MHz, DMSO-*d*6) NMR spectrum.

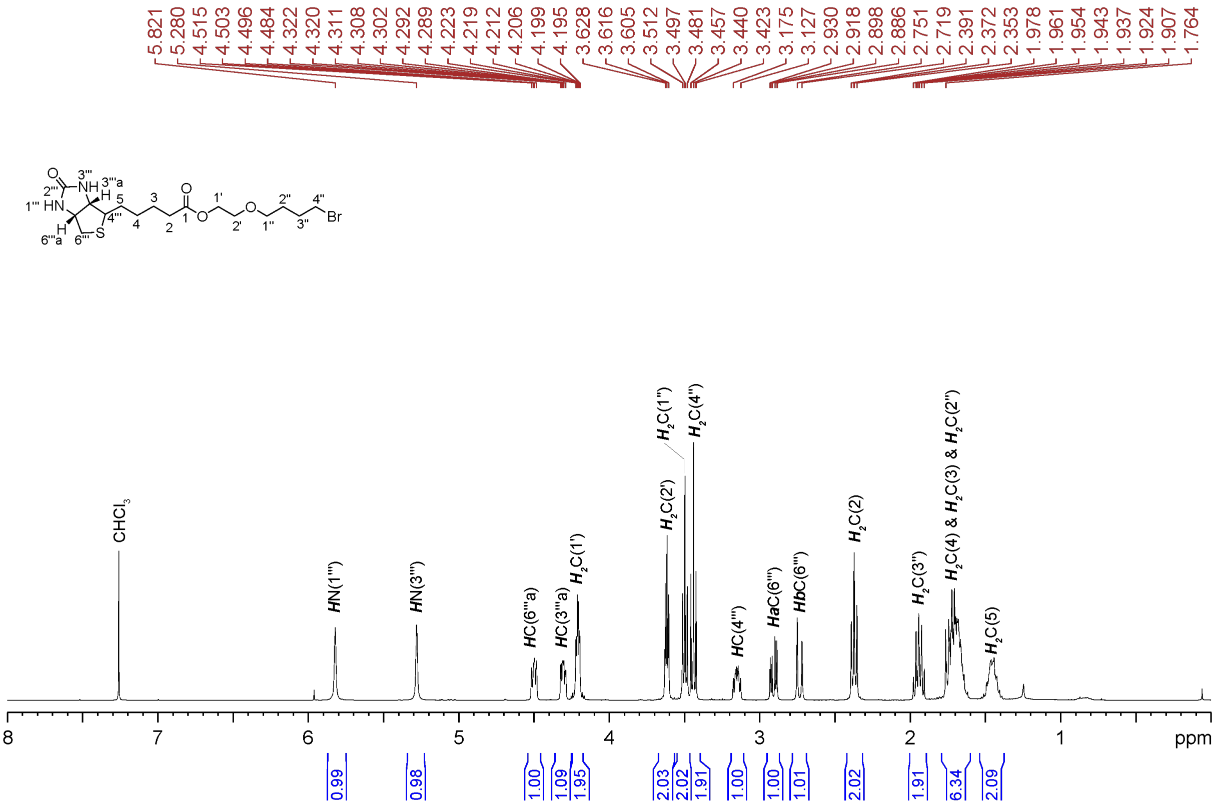

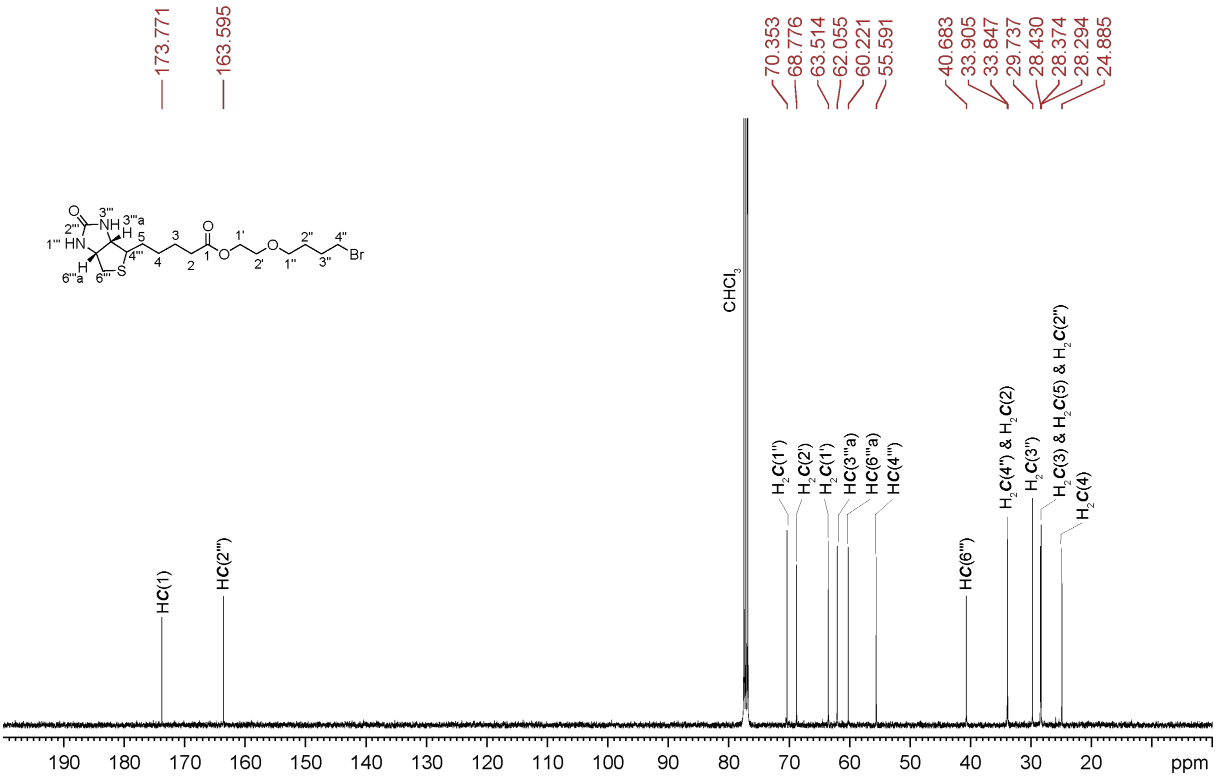

Supplementary Fig. 13 | NMR spectroscopic analysis of Br-C4-biotin. ^{1}H NMR spectrum (400 MHz, $CDCl_3$), top; ^{13}C NMR (100 MHz, $CDCl_3$) spectrum, bottom.

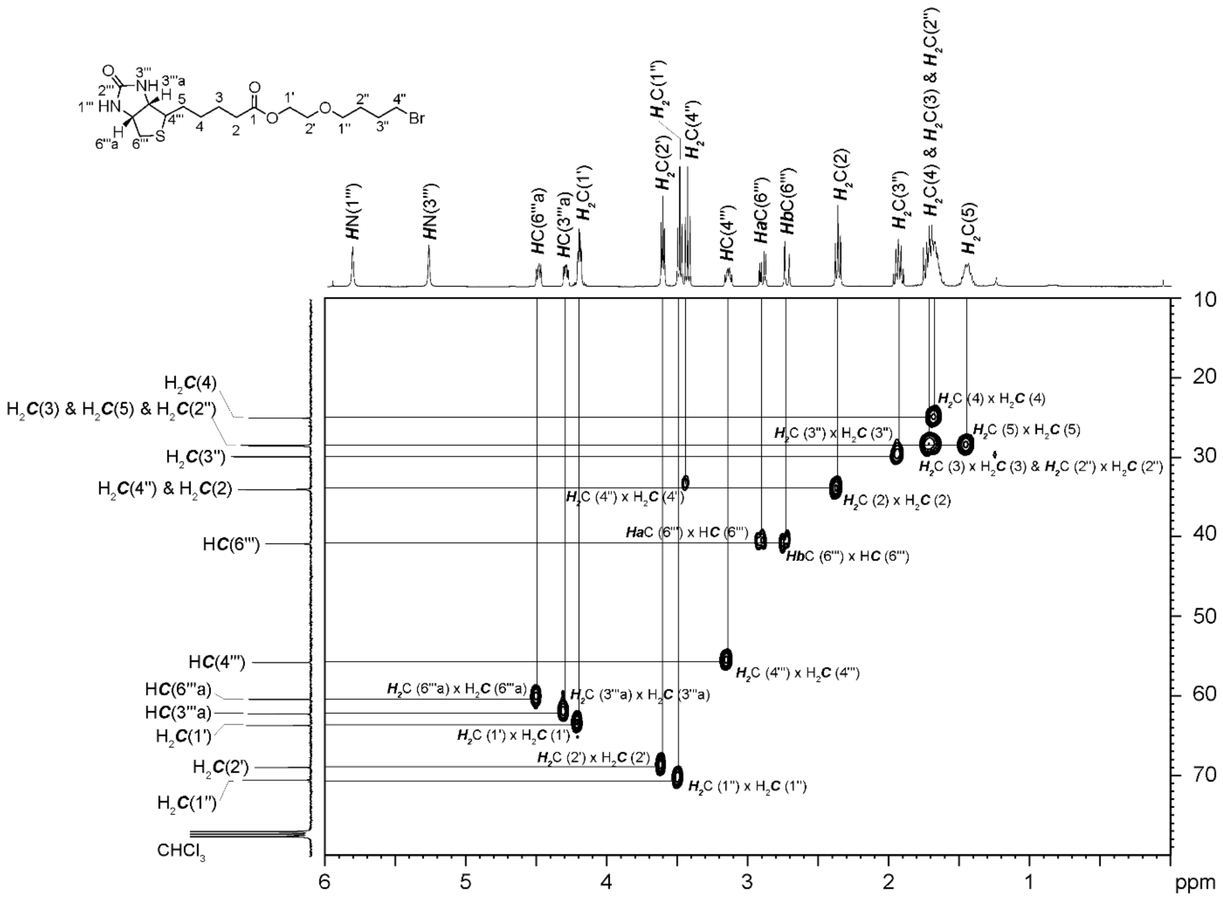

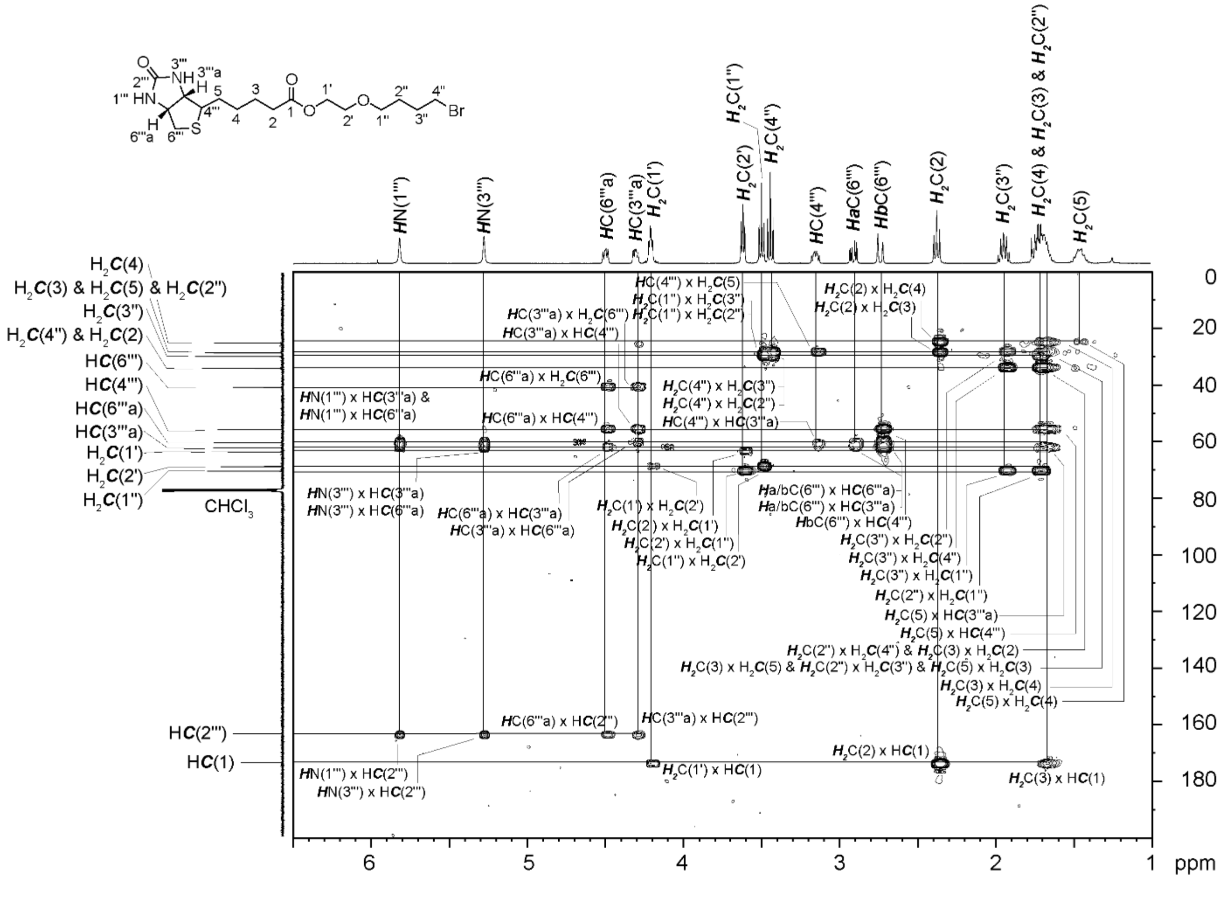

Supplementary Fig. 14 | NMR spectroscopic analysis of Br-C4-biotin. ^{1}H-^{13}C HSQC NMR (400 MHz, DMSO-d_6) spectrum, top; ^{1}H-^{13}C HMBC NMR (400 MHz, DMSO-d_6) spectrum, bottom.
