# Supplementary material for: Engineering covalent small molecule–RNA complexes in living cells
Source: Nat Chem Biol. 2025 Jan 6;21(6):843–54. doi: 10.1038/s41589-024-01801-3 (PMC12122380; doi:10.1038/s41589-024-01801-3)
Supplement: Supplementary file 5 — Source Data for Figs. 4 and 5 and Extended Data Figs. 5 and 6. [file 41589_2024_1801_MOESM5_ESM.pdf]

Source Data for Figures and Extended Figures

Source Data for Figure 4b

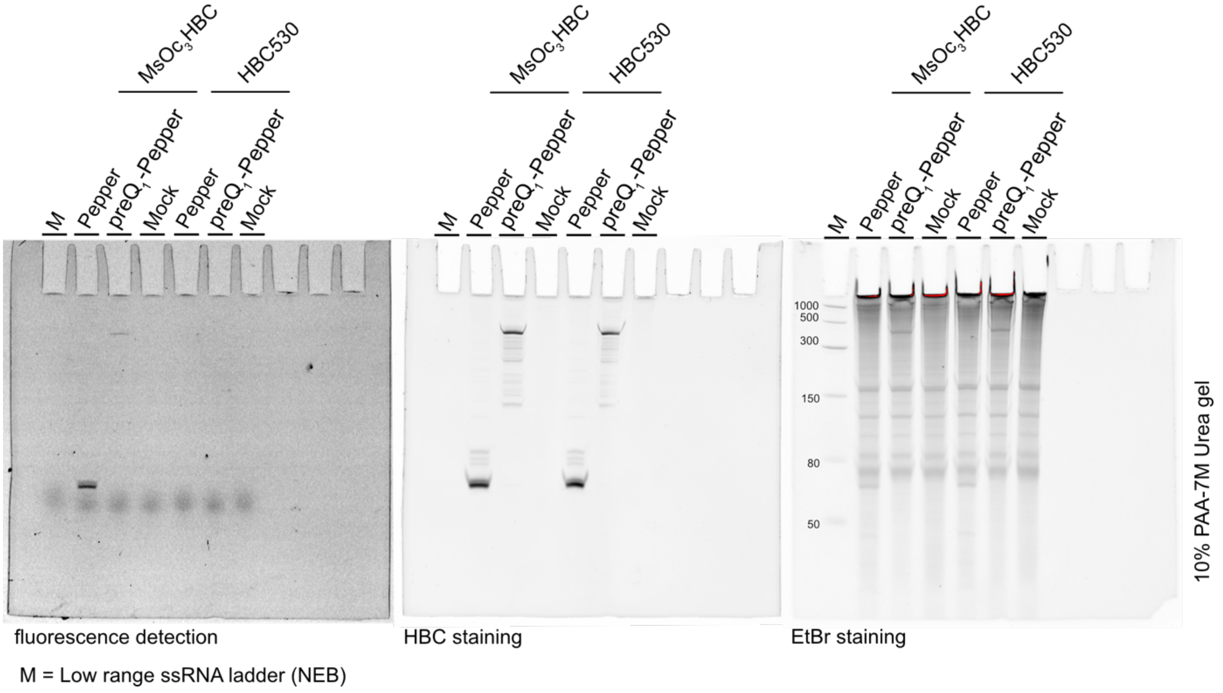

Source Data for Figure 4c

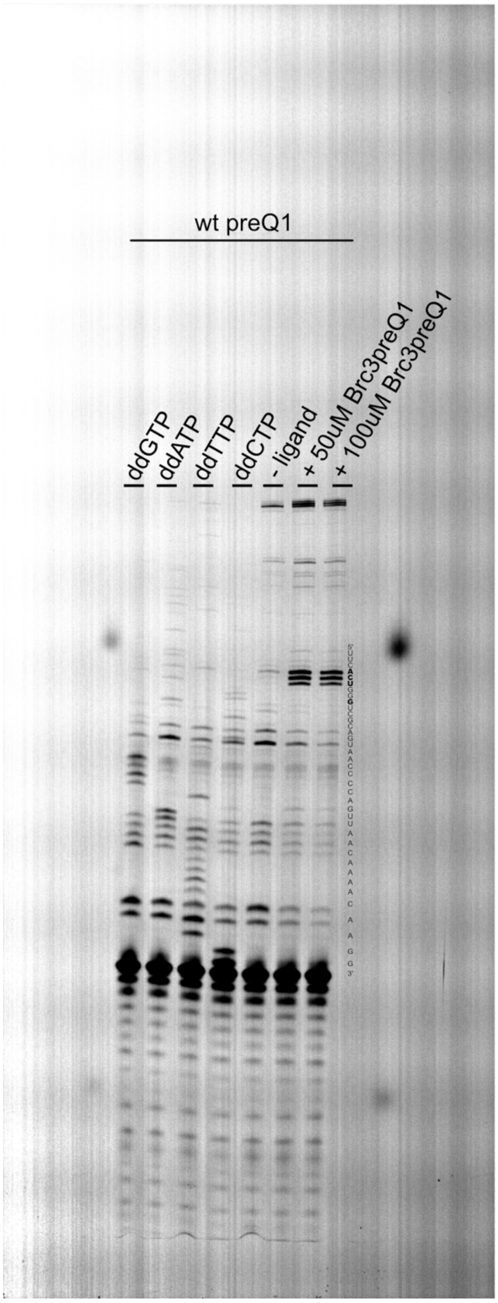

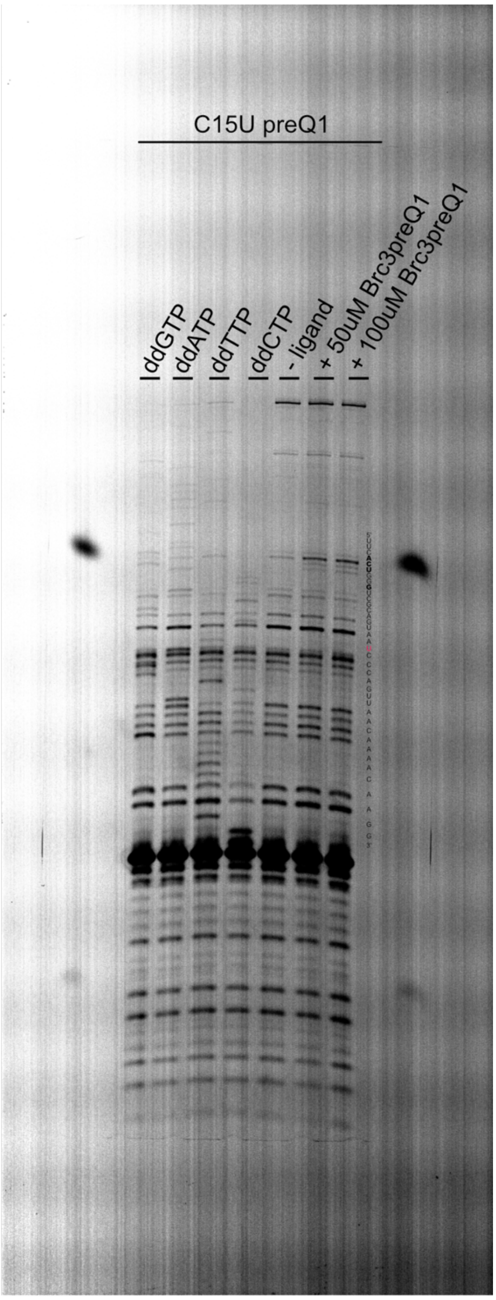

Source Data for Figure 5f

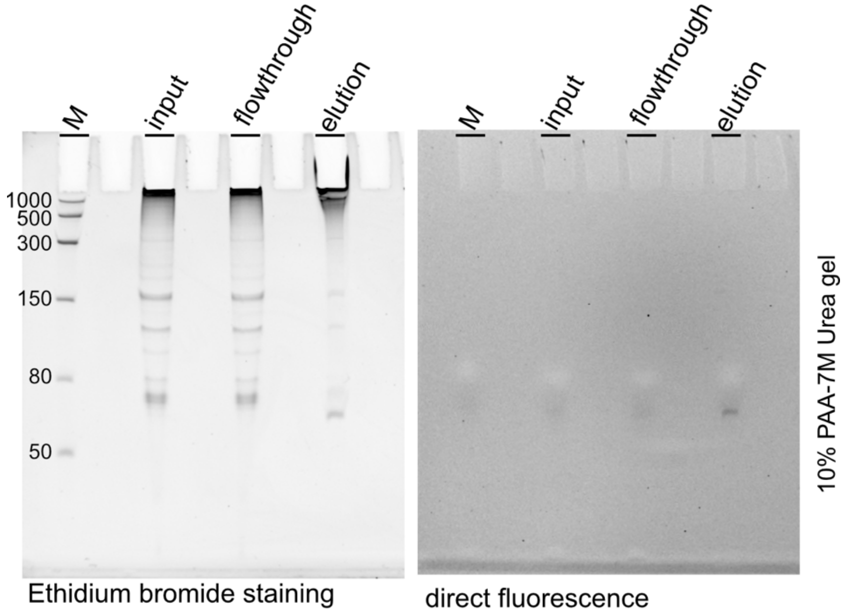

M = Low Range ssRNA ladder (NEB)

Source Data for Extended Figure 5

Experiment 1: 2.5 µM **155 nt** RNA, **50 µM** ligand, 3 h 37°C

Experiment 1: 2.5 µM **232 nt** RNA, 1 mM ligand, 2 h 37°C

Experiment 2: 2.5 µM **155 nt/232 nt** RNA, **50 µM**/1 mM ligand, 2 h 37°C

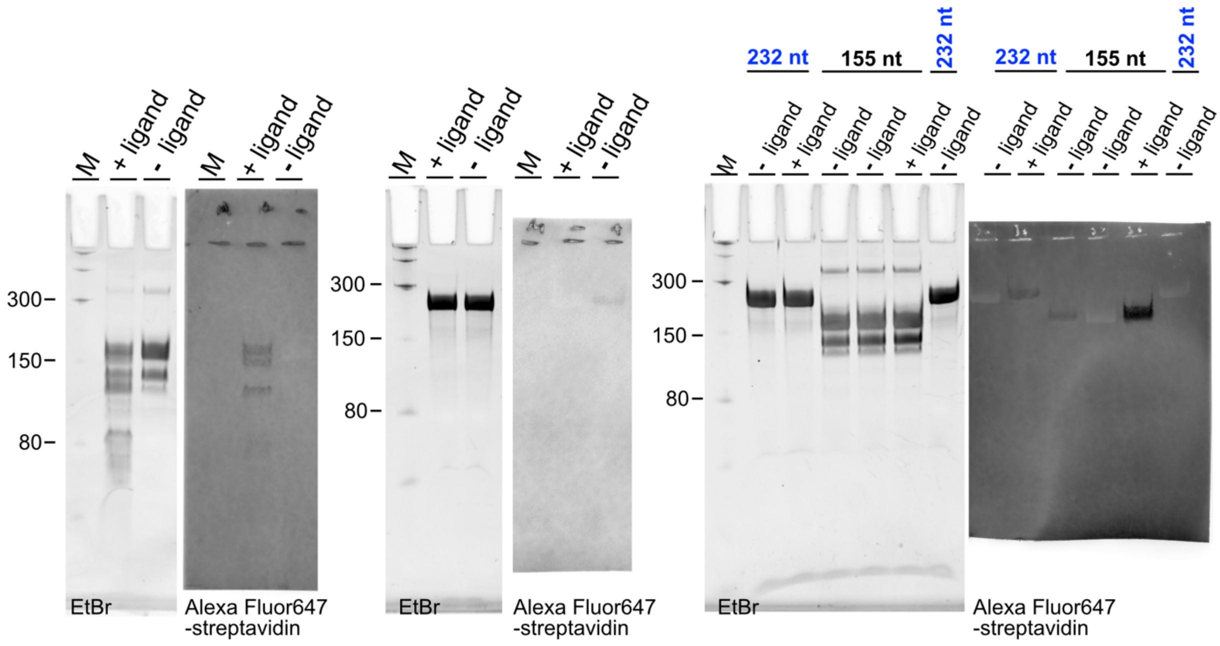

M = Low range ssRNA ladder (NEB)

Experiment 4: 2.5 µM RNA, **1 mM** ligand, 37°C

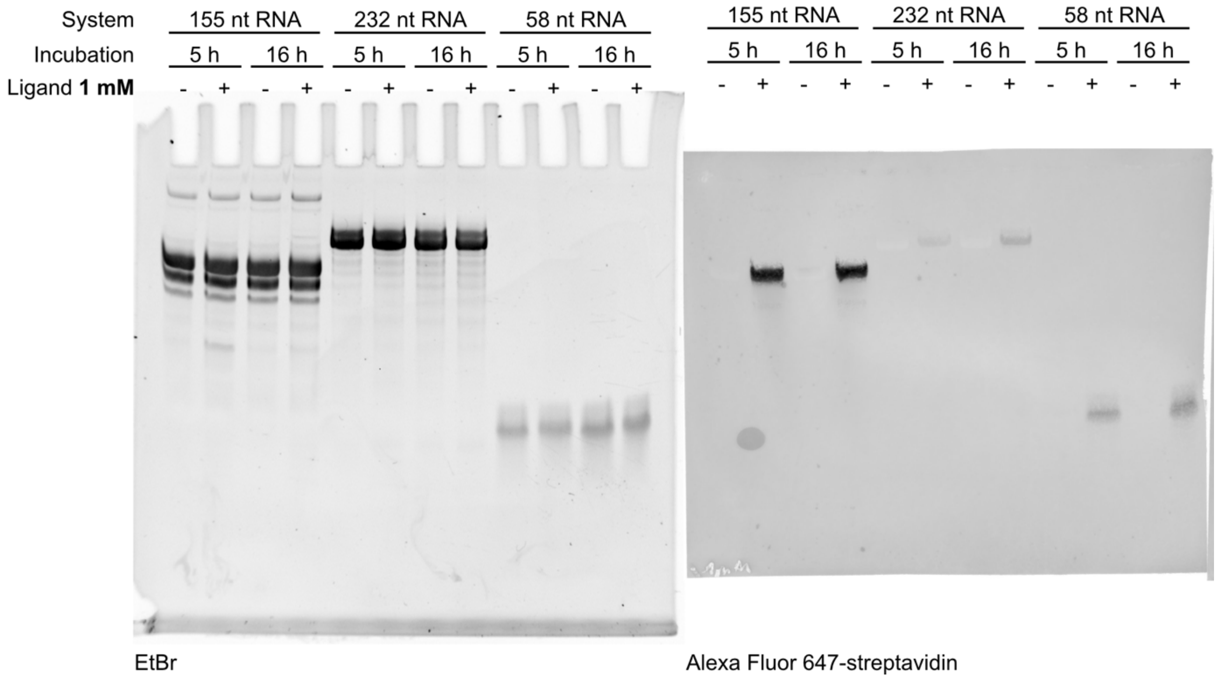

Experiment 3: 2.5 µM RNA, **50 µM** ligand, 37°C

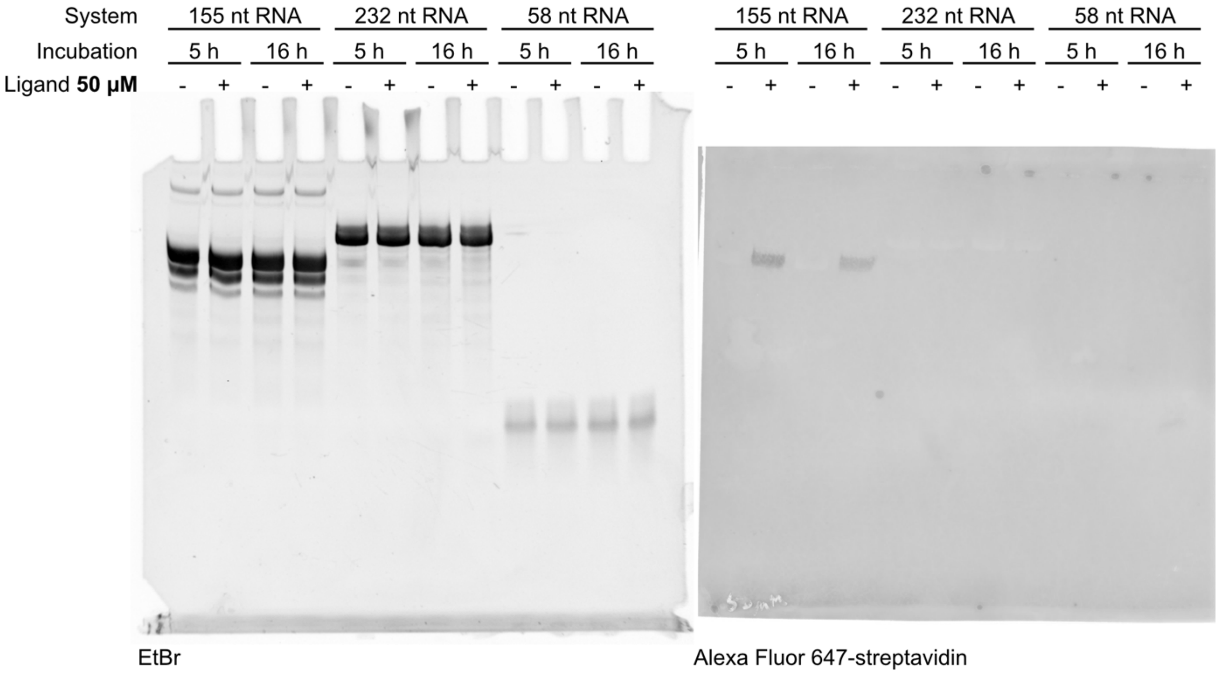

Source Data for Extended Figure 6

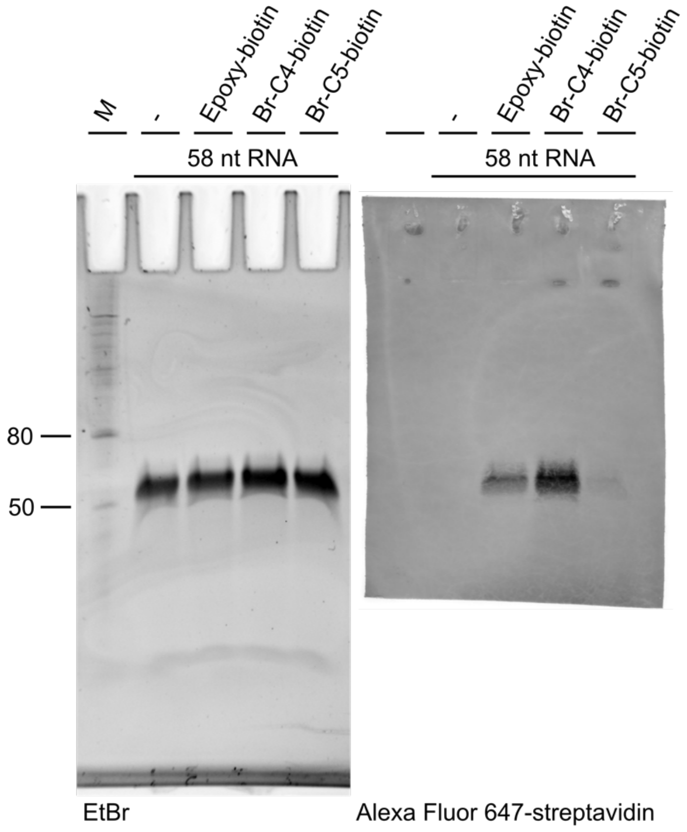

M = Low range ssRNA ladder (NEB)
